# Genomic characteristics and comparative genomics analysis of *Penicillium chrysogenum* KF-25

**DOI:** 10.1186/1471-2164-15-144

**Published:** 2014-02-21

**Authors:** Qin Peng, Yihui Yuan, Meiying Gao, Xupeng Chen, Biao Liu, Pengming Liu, Yan Wu, Dandan Wu

**Affiliations:** 1 Key Laboratory of Agricultural and Environmental Microbiology, Wuhan Institute of Virology, Chinese Academy of Sciences, Wuhan 430071, China; 2 Present address: Hubei University of Economics, Wuhan, Hubei, China

**Keywords:** *Penicillium chrysogenum*, Genome, Comparative genome

## Abstract

**Background:**

*Penicillium chrysogenum* has been used in producing penicillin and derived β-lactam antibiotics for many years. Although the genome of the mutant strain *P. chrysogenum* Wisconsin 54-1255 has already been sequenced, the versatility and genetic diversity of this species still needs to be intensively studied. In this study, the genome of the wild-type *P. chrysogenum* strain KF-25, which has high activity against *Ustilaginoidea virens*, was sequenced and characterized.

**Results:**

The genome of KF-25 was about 29.9 Mb in size and contained 9,804 putative open reading frames (*orfs*). Thirteen genes were predicted to encode two-component system proteins, of which six were putatively involved in osmolarity adaption. There were 33 putative secondary metabolism pathways and numerous genes that were essential in metabolite biosynthesis. Several *P. chrysogenum* virus untranslated region sequences were found in the KF-25 genome, suggesting that there might be a relationship between the virus and *P. chrysogenum* in evolution. Comparative genome analysis showed that the genomes of KF-25 and Wisconsin 54-1255 were highly similar, except that KF-25 was 2.3 Mb smaller. Three hundred and fifty-five KF-25 specific genes were found and the biological functions of the proteins encoded by these genes were mainly unknown (232, representing 65%), except for some *orfs* encoding proteins with predicted functions in transport, metabolism, and signal transduction. Numerous KF-25-specific genes were found to be associated with the pathogenicity and virulence of the strains, which were identical to those of wild-type *P. chrysogenum* NRRL 1951.

**Conclusion:**

Genome sequencing and comparative analysis are helpful in further understanding the biology, evolution, and environment adaption of *P. chrysogenum*, and provide a new tool for identifying further functional metabolites.

## Background

The filamentous fungus *Penicillium chrysogenum* has been widely used for producing penicillin and derived β-lactam antibiotics for more than 80 years [1]. The discovery of penicillin has greatly improved human health and promoted the development of the medical industry. In addition to producing penicillin, *P. chrysogenum* has exhibited abilities in others areas, including bioleaching, biological remediation, promoting plant growth, and producing non-β-lactam antibiotics and antifungal agents [2-6]. According to previous reports, several *P. chrysogenum* strains produce secreted proteins, such as PAF, PgAFP, and PgChP, which inhibit the growth of opportunistic zoopathogens, plant-pathogenic fungi, and toxigenic molds [7-9]. With their high stability, effective inhibitory activity, and broad inhibition spectra, these three proteins could be effective antifungal agents in medicine and agriculture [10,11].

In 2008, van den Berg et al. reported the first sequence of the *P. chrysogenum* genome and genes that were responsible for key steps in penicillin production were identified [12]. The genome not only led to a deeper understanding of penicillin synthesis, but also provided a new tool for identifying additional metabolites [13]. The sequenced *P. chrysogenum* strain Wisconsin 54-1255 was a model laboratory strain that was derived from wild-type NRRL 1951, which was isolated from infected cantaloupe [14,15]. As a mutant strain used in the laboratory, Wisconsin 54-1255 might be some genetic variations, such as reduced PahA activity, encoded by *pahA*, in the catabolism of phenylacetic acid (the side chain precursor for the synthesis of benzylpenicillin) [16]. Moreover, different *P. chrysogenum* isolates maintain diverse genetic backgrounds [17,18], and studying the genome sequences of other strains will providemore information on the genetic diversity of *P. chrysogenum*. Therefore, sequencing the genome of a wild-type *P. chrysogenum* strain is necessary.

*P. chrysogenum* KF-25 is a wild-type strain isolated from a soil sample by our laboratory. It shows high-anti-fungal activity against *Ustilaginoidea virens*, which causes false smut disease of rice and corn in humid areas [19], in contrast to the Wisconsin 54-1255 strain, which did not exhibit anti-fungal activity. This suggested that there might be differences in the genetic backgrounds of the two strains. To provide more genetic information on *P. chrysogenum* to identify additional active substances and to determine the critical genes involved in the biosynthesis of the active substances, we sequenced and analyzed the genome of KF-25. Comparative genome analysis of strain KF-25 with Wisconsin 54-1255 and the wild-type strain NRRL 1951 revealed significant genetic variance. We also analyzed the functions and distribution of the genes encoding several important proteins, including transporters, non-ribosomal peptide synthase, and two-component regulatory systems (TCRSs).

## Results and discussion

### Strain features

**Figure 1:**
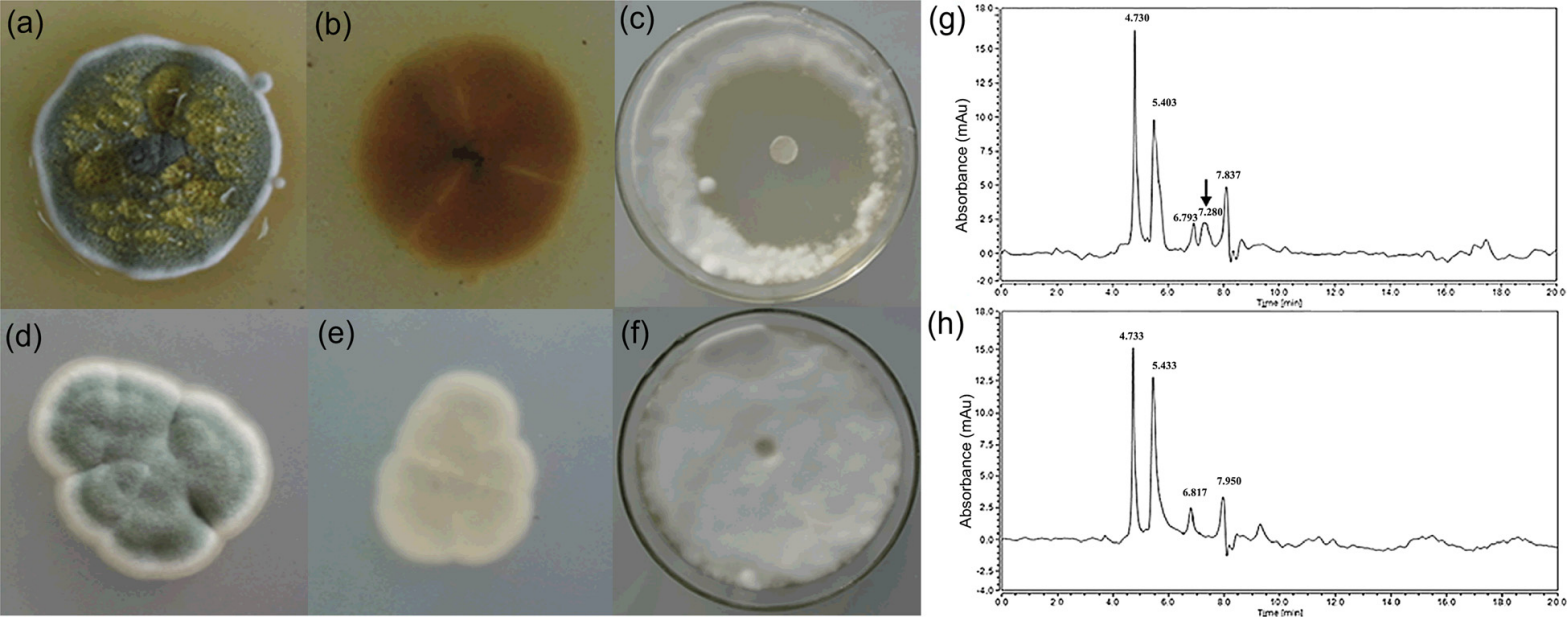
**Colony features and anti-fungal activity of KF-25 and Wisoconsin 54-1255. (a)** and **(d)**, the front colony morphology on PSA plates after 5 days of growth. **(b)** and **(e)**, the back of the colony on PSA plates after 5 days of growth. **(c)** and **(f)**, the inhibitory activity of the 4-day fermentation broths on *U. virens* strain UV-1. **(g)** and **(h)**, the HPLC chromatogram of the four day fermentation broths. The upper row indicated the features of KF-25 and the lower row indicated the feature of Wisconsin 54-1255.

The colony morphology and anti-fungal activity of strains KF-25 and Wisconsin 54-1255 were investigated. Following grown on potato-Sucrose-agar (PSA) plates for 5 days, flavescent water drops were observed on the surface of KF-25 colonies, but not on Wisconsin 54-1255 (Figure 1a,d). Strain KF-25 also produced more yellow pigment than Wisconsin 54-1255 (Figure 1b,e). The anti-fungal activities of the two strains against *U. virens* strain UV-1 were tested, and results showed that strain KF-25 had a strong inhibitory effect on UV-1 (Figure 1c), while no anti-fungal activity was observed for strain Wisconsin 54-1255 (Figure 1f). The fermentation broth of KF-25 and Wisconsin 54-1255 was analyzed by using HPLC-DAD and an additional peak was observed at time point 7.28 min in the HPLC chromatogram of KF-25 (Figure 1g,h). The component was collected from time point 7 to 8 min and the collected component showed a high activity against UV-1 (data not shown). As strain KF-25 is a wild isolate and Wisconsin 54-1255 is a mutant strain derived from NRRL 1951 [13], the different origins might cause the different physiological features.

### Genome sequence and annotation of *P. chrysogenum* KF-25

#### General genome features

**Figure 2:**
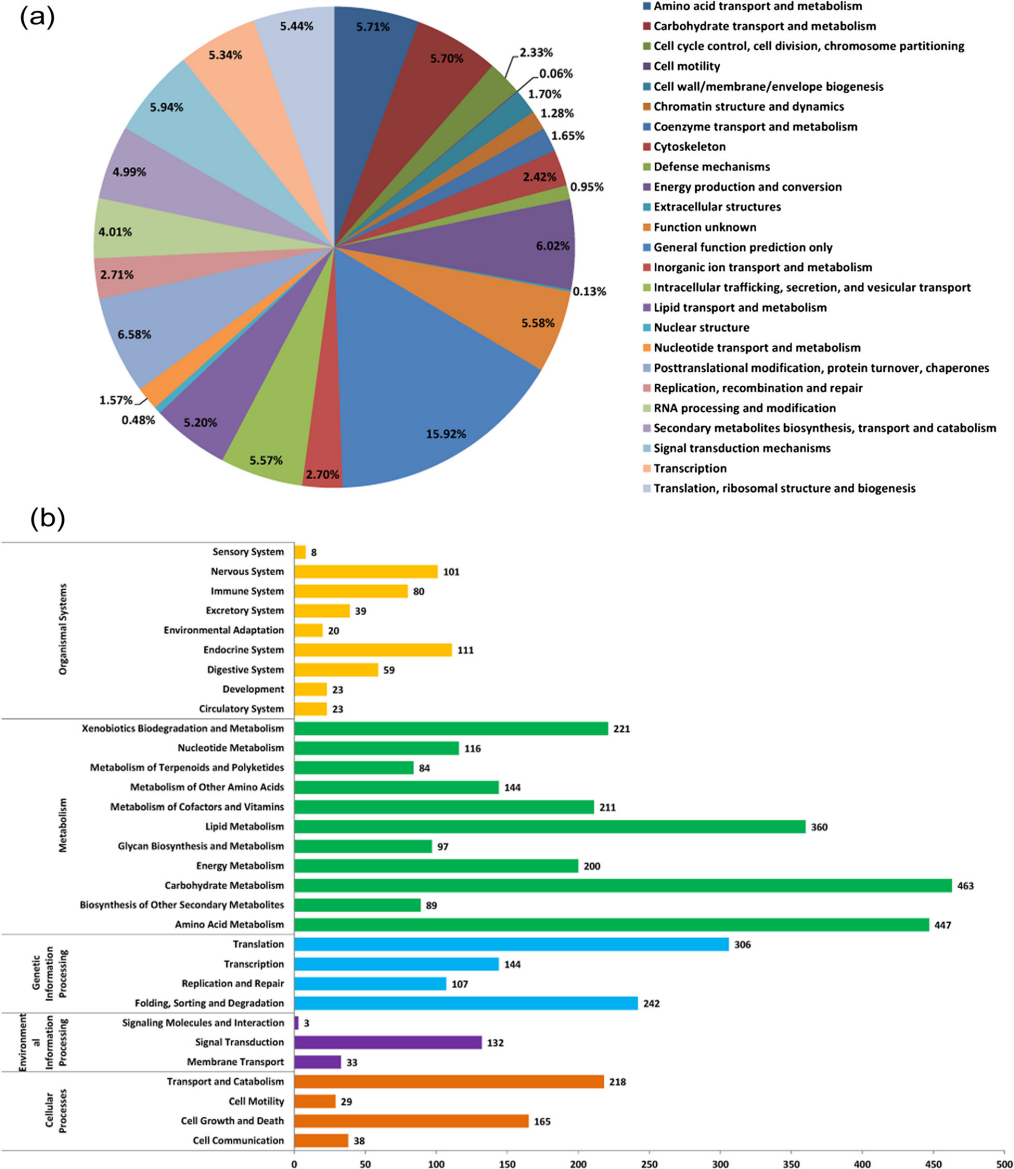
**Characteristics of the genome of *P. chrysogenum *KF-25. (a)** Functional classification of ORFs encoded by the genome of strain KF-25, based on the KOG (Eukaryotic Orthologous Groups of proteins) database. In total, 6,231 ORFs with orthologs in the KOG database were classified and the percentages indicate the frequencies of ORFs with assigned functions. **(b)** Functional classification of KF-25 genome ORFs based on the KEGG (Kyoto Encyclopedia of Genes and Genomes) database. In total, 4,158 ORFs had functional classifications assigned and the numbers with each classification are indicated.

The genome of *P. chrysogenum* KF-25 was sequenced by a shotgun approach using Hiseq 2000 (Illumina, California, USA) with a read length of 2 × 100 bp. The 29.9 Mb genome was covered by 194 scaffolds and composed of 1,459 contigs with 154× coverage. Among the 194 scaffolds, the average length was 154 kb, with the largest being 2.72 Mb. The general features of the KF-25 genome compared with the Wisconsin 54-1255 genome are shown in Table 1. Genome annotation revealed that the genome of strain KF-25 encoded 9,804 ORFs, and that the GC content of the predicted protein-coding region was 53.4%. Among the 9,804 ORFs, 7044 were similar to proteins in UniProt database, 4,158 proteins were similar to proteins in the KEGG database (Figure 2), and 9,727 showed similarity to proteins from the NCBI nr database. Analysis of the 9,804 ORFs by KOGnitor indicated that 6,231 predicted proteins matched members of the eukaryotic orthologous groups (KOG) (Figure 2). In the genome of strain KF-25, 112 genes encoding tRNA and 29 rDNA genes were predicted using tRNAscan and RNAmmer software. The 112 tRNA genes were mainly scattered between scaffolds 1, 9, 15, 18, 19, 24 and 51, although sometimes four or five tRNA genes formed clusters. The anticodon usage of KF-25 is listed in Additional file 1: Table S1. Among the 9,804 predicted ORFs, 39 and 91 were identified as translation and transcription factors, respectively. (Additional file 1: Table S2 and Table S3).

**Table 1:** **General genome features of *P. chrysogenum *** **KF-25 and *P. chrysogenum *** **Wisconsin 54-1255**

**Genome features**	***P. chrysogenum *** **KF-25**	***P. chrysogenum *** **Wisconsin 54-1255 [**[12]**]**
Assembly sizes (Mb)	29.9	32.2
GC content (%)	49.0	48.9
Gene number	9,804	12,943
Mean gene length (bp)	1,573	1,515
Exons per gene	3.2	3.0
Introns per gene	2.2	2.2
tRNA number	112	145
rRNA number	29	28

In total, 317 repetitive elements were found in the genome of strain KF-25 by RepeatScout, with a minimum length 50 bp and a maximum length of 1,296 bp. Repetitive sequence analysis by using CENSOR indicated that 648,249 bp of the KF-25 genome (2.17%) was repeat sequences, while the repeat content of Wisconsin 54-1255 was 1.04% [12].

Microsatellites (simple sequence repeats, SSRs) are one of the most popular genetic markers and exist widely in fungal genomes. Because of high mutation rate and changing in repeat numbers during DNA replication, SSRs exhibit high individual specificity [20-22]. In the genome of KF-25, 3,798 SSRs were found, with sizes ranging from 15to 167 bp, and these SSRs were homogenously distributed throughout the genome (Additional file 1: Figure S1).

#### The secretory system and transporter

Translocation of protein and molecule across the plasma membrane is essential for cell life and requires the help of secretory systems and transporters, such as signal recognition particle (SRP) and the Sec translocase [23,24]. SRP plays a critical role in targeting of secretory proteins to the cellular membrane [25], while the Sec secretion system is responsible for protein translocation across the cytoplasmic membrane [26]. *P. chrysogenum* has been widely used to produce penicillin and some other secondary metabolites with antimicrobial activity [2,7-9,27,28]. The secretory system and transporters are essential for secretion of these antimicrobial substances and for import of their substrates. In the KF-25 genome, 12 proteins were predicted to be components of the eukaryotic Sec-SRP secretion systems (Additional file 1: Table S4). These proteins might play important roles in protein secretion in *P. chrysogenum*. Several genes in the genome of KF-25 encoded transporters or components of the secretion system that involved in producing penicillin and other secondary metabolites. KF-25 genome contained 531 genes that encoded transporter proteins, which mainly belonged to the major facilitator superfamily (MFS, 231 genes), and the ABC transporter superfamily (52 genes). Several genes in the secondary metabolism gene cluster were predicted to encode MFS-type transporters by antiSMASH [29]. The MFS transporters in the penicillin synthesis pathway could regulate the production of penicillin and enhance the sensitivity of *P. chrysogenum* to phenylacetic acid [30]. Many ABC superfamily transporters in the KF-25 genome were predicted to be multidrug resistance proteins [31]. One ABC superfamily transporter was reported to be critical in the export of phenylacetic acid, which is the precursor of penicillin synthesis. There were also several other transporters in the KF-25 genome that are involved in sugar, amino acid, cation, and vitamin transport.

#### Two-component regulatory system

TCRSs (Two-component regulatory systems) are found in bacteria, yeast, fungi and plant, and enable organisms to rapidly sense and adapt to specific environments [32]. TCRSs consisted of a sensor kinase and a response regulator, and are involved in regulating diverse processes, such as chemotaxis, osmolarity and differentiation [33-35]. According to previous reports, osmotic pressure regulates the morphogenesis and the secondary metabolism pathways of filamentous fungi via TCRSs [33,36,37]. Increased osmotic pressure stimulated the vegetative growth and conidia formation of *P. chrysogenum*, and also influenced its respiration and organic acid production [38,39]. The TCRSs that senses osmotic pressure and regulates the life cycle of *P. chrysogenum* might induce *P. chrysogenum* produce secondary metabolites, such as penicillin and other bioactive agents. Thirteen predicted proteins based in KF-25 were involved in TCRSs. Among these proteins, four contained both the sensor kinase and response regulator domains, four contained only the sensor kinase domains, and the remaining five contained only the response regulator domains (Table 2). Six of the 13 predicted proteins were involved in TCRSs that sensed and adapted to the osmotic pressure. Similar proteins were also found in the genome of Wisconsin 54-1255 [12] . The existence of osmotic pressure-associated TCRSs in the *P. chrysogenum* genome might explain the ability of *P. chrysogenum* to adapt to high osmotic pressure. The other seven predicted TCRSs proteins were mainly involved in sensing or adapting to drugs, the cell cycle, or capsular synthesis.

**Table 2:** **The genes encoding the proteins involving in the two-component systems in the genome of *P. chrysogenum *** **KF-25 and corresponding genes in the genome of *P. chrysogenum *** **Wisconsin 54-1255**

**Genes in Genome of KF-25**	**Putative protein function**	**Corresponding genes in *P. chrysogenum *** **Wisconsisn 54-1255**
KF25_0355	osmolarity two-component system, response regulator SSK1	Pc20g02430 (98% identify)
KF25_1660	two-component system, NarL family, capsular synthesis sensor histidine kinase RcsC	Pc22g18780 (99% identify)
KF25_2492	osmolarity two-component system, response regulator SKN7	Pc22g04440 (99% identify)
KF25_4368	two-component system, chemotaxis family, sensor kinase Cph1	Pc06g00040 (99% identify)
KF25_4886	two-component system, unclassified family, sensor histidine kinase and response regulator	Pc16g03520 (99% identify)
KF25_7115	two-component system, cell cycle sensor kinase and response regulator	Pc12g07950 (96% identify)
KF25_7139	two-component system, NarL family, capsular synthesis sensor histidine kinase RcsC	Pc22g07510 (99% identify)
KF25_7934	osmolarity two-component system, phosphorelay intermediate protein YPD1	Pc22g12510 (100% identify)
KF25_8216	osmolarity two-component system, response regulator SSK1	Pc22g16340 (99% identify)
KF25_8339	osmolarity two-component system, response regulator SSK1	Pc13g13580 (88% identify)
KF25_8360	osmolarity two-component system, response regulator SSK1	Pc13g13880 (99% identify)
KF25_9319	two-component system, unclassified family, sensor histidine kinase and response regulator	Pc13g09080 (99% identify)
KF25_9723	two-component system, unclassified family, sensor histidine kinase and response regulator	Pc20g15550 (99% identify)

### Comparative genomics and phylogenetic analysis of *P. chrysogenum* KF-25

#### Comparative genome analysis of P. chrysogenum KF-25 and P. chrysogenum Wisconsin 54-1255

**Figure 3:**
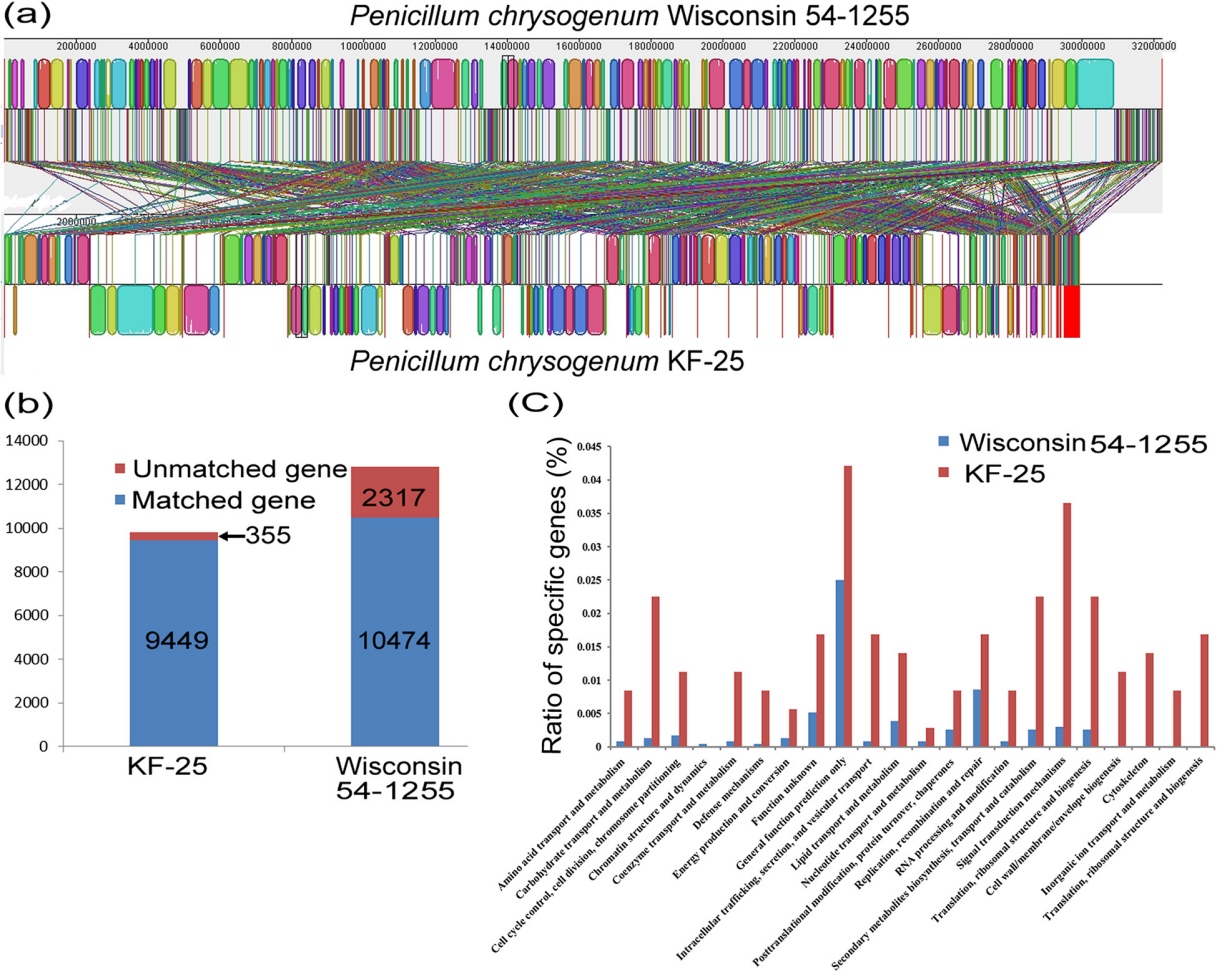
**Comparative genome analysis of *P. chrysogenum *KF-25 and Wisconsin 54-1255. (a)** Alignment of the KF-25 and Wisconsin 54-1255 genomes using Mauve 2.3.1. The line above indicates the position of the genome in Wisconsin 54-1255. The rectangles represent the genome fragments and the white gaps between the rectangles mean no similar fragments were found in the other genome. **(b)** Comparation of the orthologous genes between the genomes of *P. chrysogneom* KF-25 and Wisconsin 54-1255. Numbers of genes with orthologs found in the other genome are represented in blue and numbers of genes with no ortholog genes found were represented in red. The vertical axis indicates the number of genes. **(c)** KOG classification of the specific genes of KF-25 and Wisconsin 54-1255. The vertical axis indicates the percentage of genes among the specific genes (2,317 specific genes for Wisconsin 54-1255 and 355 specific genes for KF-25) and the horizontal axis indicates the classification of the gene in the KOG database.

**Figure 4:**
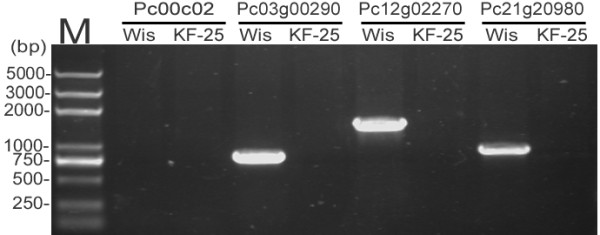
**PCR detection of the *P. chrysogenum *** **Wisconsin 54-1255-specific genes from the genome of KF-25 and Wisconsin 54-1255.**

The genome of *P. chrysogenum* KF-25 was 2.3 Mb smaller than that of *P. chrysogenum* Wisconsin 54-1255 (Table 1). The genome of KF-25 was composed of 194 scaffolds, while the genome of Wisconsin 54-1255 was composed of only 49 super-contigs [12]. We speculated that gaps between the scaffolds might be one of the reasons for the smaller genome size of KF-25. Genomic alignment showed that the genome of KF-25 covered 93% of the Wisconsin 54-1255 genome. The average protein similarity between the predicted proteomes of KF-25 and Wisconsin 54-1255 was 75.1% (Figure 3a; Additional file 1: Figure S2). Several genome fragments, with a total length of 2.3 Mb, were missing in the KF-25 genome. These fragments in the Wisconsin 54-1255 genome were mainly from the 5′- termini of contigs 13, 17, 23, 24 and the 3′-terminus of contig 22. According to a previous report, these fragments of the Wisconsin 54-1255 genome were not found in the genomes of other sequenced filamentous fungi, such as *Aspergillus nidulans, Aspergillus niger*, and *Aspergillus oxyaze*[12], and were proposed to contain the *P. chrysogenum*-specific genes [12]. Alignment of the proteomes of the two strains showed that 2, 317 genes in the genome of Wisconsin 54-1255 were not found in the genome of KF-25 (Figure 3b), while 1,043 (representing 45%) of these genes were located in the 2.3 Mb of missing fragments. Based on these results, we inferred that these genes were not the *P. chrysogenum*-specific genes, but of Wisconsin 54-1255 strain-specific genes. The biological functions of most proteins encoded by these strain-specific genes are unknown (2183, representing 94.2%) [12], though some genes were involved in transport, metabolism, and transcription regulation (Figure 3c; Additional file 1: Table S5 and Figure S3A). The 2.3 Mb of missing fragments contained numerous repeat and transposable elements, and the introns in these regions were typically small and few compared with other regions of Wisconsin 54-1255 genome. Because the two sequenced *P. chrysogenum* strains were isolated from different geographical regions, and because Wisconsin 54-1255 is a laboratory strain tht has undergone several rounds of mutation, the strain-specific sequences in the Wisconsin 54-1255 genome might have evolved by transposition and horizontal gene transfer. Furthermore, there were 355 strain-specific genes in KF-25 genome that were not found in the genome of Wisconsin 54-1255 (Figure 3b). These KF-25 strain-specific genes mainly exhibited high levels of similarity to genes from *Aspergillus* species and *Neosartorya fischeri* (Additional file 1: Figure S4), which are evolutionarily closely related to *P. chrysogenum*[12]. The biological functions of the 355 KF-25-specific genes are mainly unknown (232, representing 65%), except for some ORFs that were predicted to be involved in transport, metabolism, and signal transduction (Figure 3c; Additional file 1: Table S6 and Figure S3B). Among the 355 ORFs, none were found to be involved in cell mobility, extracellular structures, chromatin structure, and metabolism (Figures 2 and 3c), but ORFs with functions in intracellular trafficking, secretion, vesicular transport, signal transduction, and transcription were frequently found. To confirm that the 2.3 Mb of DNA fragments were truly missing from the KF-25 genome, three randomly chosen Wisconsin 54-1255 strain-specific genes (Pc03g00290, Pc12g02270 and Pc21g20980) from these fragments of were investigated using PCR amplification. The results showed that these genes were detected in the Wisconsin 54-1255 genome but not in the KF-25 genome (Figure 4). Another one Wisconsin 54-1255-specific gene, Pc00c02 [GenBank:AM920417.1], which is annotated as a 16S ribosomal RNA was not detected in either genome. The 16S ribosomal RNA gene is widely used to classify bacteria and is reported to only exist in bacterial genomes [40]. BLAST analysis of Pc00c02 indicated that it was highly similar to the 16S rDNA region of bacteria *Rugosimonospora sp.* 260305 (100% identify) and *Micromonospora sp.* HBUM80369 (99% identify). The 16S rDNA found in the Wisconsin 54-1255 genome sequence might be caused by bacterial contamination during sequencing.

#### Comparative analysis of P. chrysogenum KF-25 and other P. chrysogenum strains

According to previous proteomic studies, the improvement process of penicillin production enhanced the expression of some genes, while decreasing [15,41,42]. *P. chrysogenum* Wisconsin 54-1255 is a moderately improved penicillin producer derived from the wild-type *P. chrysogenum* NRRL 1951, which exhibited more secondary metabolism pathways (such as pigments), pathogenicity proteins and virulence proteins compared with Wisconsin 54-1255 and another high penicillin producer *P. chrysogenum* AS-P-78 [15,42]. *P. chrysogenum* KF-25 is a wild-type strain that had a stronger yellow pigment production than Wisconsin 54-1255 (Figure 1). The ability to produce more pigments is representative of a greater number of secondary metabolic pathways, and was a common feature of both KF-25 and NRRL 1951. Several KF-25-specific genes were found to be associated with pathogenicity and virulence. One such gene, KF25_6369, which encodes glucose oxidase, is thought to be involved in virulence because gluconic acid and glucose oxidase are related to pathogenicity of *Penicillium espansum* in apples [43]. Glucose oxidase also showed reduced expression in Wisconsin 54-1255, compared with NRRL 1951 [42]. The penicillin synthesis genes were clustered in one group in the genomes of NRRL 1951 and Wisconsin 54-1255, while several such clusters were found in the AS-P-78 genome [44]. Similar to wild-type NRRL 1951, KF-25 contained only one penicillin synthesis gene cluster. Wild-type *P. chrysogenum* KF-25 and NRRL 1951 have more secondary metabolism pathways and more pathogenicity and virulence associated genes, which are fitness mechanisms for the wild-type strains to survive in natural environment.

#### Phylogenetic analysis of P. chrysogenum KF-25 and the other sequenced filamentous fungi

**Figure 5:**
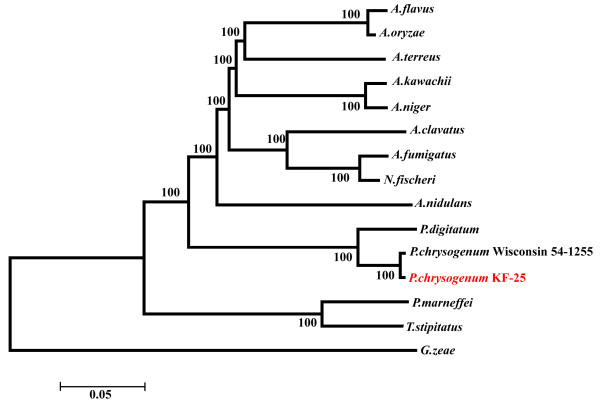
**Phylogenetic analysis of *P. chrysogenum *and other sequenced filamentous fungi.** The phylogenetic tree was constructed by concatenating the amino acid sequences of 90 conserved proteins from all strains using the neighbor-joining method and bootstrap analysis (1,000 replicates) of the ClustalW alignment. Gibberella zeae was used as an outgroup strain. The support rates are indicated at the node of each branch and the scale bar represents 0.05 substitutions per amino acid position.

A concatenated set of the amino acid sequences of 90 conserved proteins was used to construct a phylogenetic tree [12]. The phylogenetic analysis (Figure 5) showed a close relationship between KF-25 and *Aspergillus* species, and a more distant evolutionary relationship between KF-25 and *Penicillium marneffei* and *Talaromyces stipitatus. P. chrysogenum* KF-25 was in the same evolutionary branch as Wisconsin 54-1255 and showed a close relationship with *Penicillium digitatum*. This result was consistent with previous reports [12,45]. A phylogenetic tree constructed based on the amino acid sequences of the β-tubulin also supported the evolutionary relationship of strains from the *Penicillium* genus (Additional file 1: Figure S5).

### Secondary metabolism analysis of *P. chrysogenum* KF-25

#### Putative secondary metabolism pathways

*P. chrysogenum* has been known as a penicillin producer for many years [1]. Recently, studies have mainly focused on the pathways of penicillin synthesis, and the key genes involving involved in penicillin production have been determined [13,27,46]. In additional to penicillin, *P. chrysogenum* can produce many other secondary metabolites, such as mycotoxin and drugs [27,28,47,48]. In a previous report, SMURF analysis predicted that the genome of Wisconsin 54-1255 contains 33 secondary metabolism gene clusters [49]. In this study, secondary metabolism gene clusters was predicted using antiSMASH [29], and 33 and 41 gene clusters were identified in the genomes of KF-25 and Wisconsin 54-1255 (Additional file 1: Table S7 and Figure S6). The predicted products of 23 secondary metabolism gene clusters in KF-25 were: eight nonribosomal peptides, 10 polyketides, two hybrid non-ribosomal peptide synthase (NRPS)-polyketide synthases (PKS), one hybrid NRPS-terpene, one terpene and one siderophore, while the remainding 10 gene clusters produced other secondary metabolites (Additional file 1: Table S7). Among the 33 gene clusters, five were predicted to produce stigmatellin, chalcomycin, epothilone, fumitremorgin and penicillin. The production of penicillin by KF-25 and Wisconsin 54-1255 were verified by HPLC (Additional file 1: Figure S7). The data showed that Wisconsin 54-1255 exhibited greater ability of producing penicillin than KF-25.

#### Non-ribosomal peptide synthetase

NRPSs play important roles in the synthesis of non-ribosomal peptides, which include antibiotics and other important pharmaceuticals [50]. In the *P. chrysogenum* KF-25 genome, 20 NRPS genes were found and the domain compositions of these predicted NRPSs are shown in Additional file 1: Figure S8. Among the 20 predicted NRPSs, 14 were involved in putative secondary metabolism pathways, while the other six were not. Eleven of the 20 predicted NRPSs, encoded by gene KF25_6155, KF25_1342, KF25_6525, KF25_1526, KF25_9456, KF25_5703, KF25_8966, KF25_6509, KF25_8398, KF25_9347, and KF25_4993, had similar amino acid sequences to HC-toxin synthase. In addition, 15 of the predicted MFS transporters encoded by the KF-25 genome were identified as HC-toxin efflux carriers. The HC-toxins determine the specificity and virulence of pathogenic fungi toward host plants [51]. The existence of the HC-toxin synthases and HC-toxin efflux carriers suggested that *P. chrysogenum* KF-25 might produce HC-toxin.

#### Polyketide synthase

**Figure 6:**
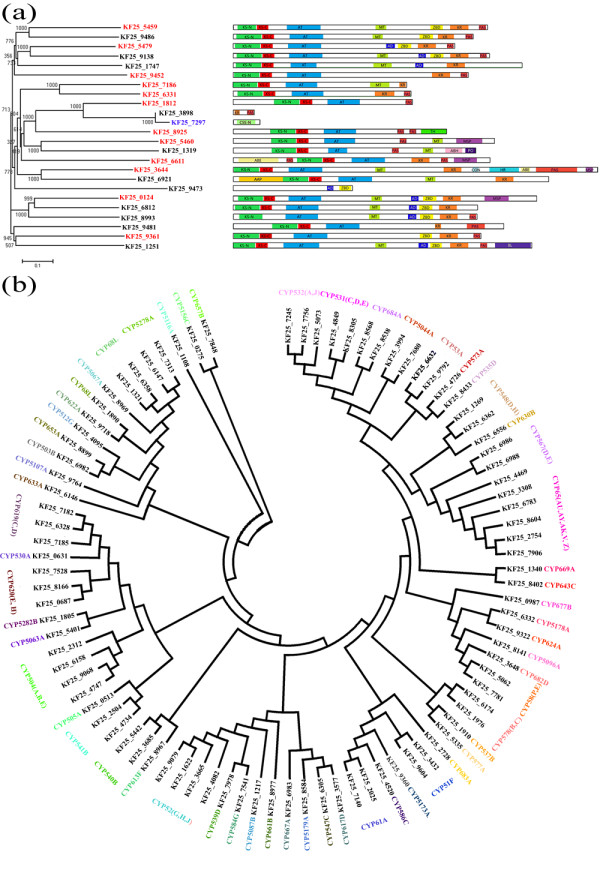
**Neighbor-joining phylogenetic tree of polyketide synthases (PKSs) and cytochrome P450 from the KF-25 genome.** The amino acid sequences of the proteins were used to construct the phylogenetic tree using ClustalX2.0 with the neighbor-joining method. The branch length scale bar below the phylogenetic tree indicates the number of substitutions per amino acid site. **(a)** The functional domain architecture of proteins was predicted using Pfam and AMSPKS [89,90] and is shown on the right. Protein domain names were as follows: KS_N, β-ketoacyl synthase, N-terminal domain; KS_C, β-ketoacyl synthase, N-terminal domain; AT, acyl transferase; KR, β-keto reductase; PAS, phosphopantetheine attachment site; MT, methyltransferase; MSP, male sterility protein; TH, thioesterase; ZBD, zinc-binding dehydrogenase; ER, ER domain; AD, alcohol dehydrogenase GroES-like domain; CSS_N, chalcone and stilbene synthases, N-terminal domain; ABH, α/βhydrolase; PO, prolyl oligopeptidase; CON, condensation domain; HR, HxxPF-repeated domain; ABE, AMP-binding enzyme; BL, β-lactamase; AAP, amino acid permease. The ORFs indicated in red (type I PKS) and blue (type II PKS) are the members of the putative secondary metabolism pathways. **(b)** The cytochrome P450 (CYPs) identified in the KF-25 genome and CYPs of different families are indicated in different colors. The families of the corresponding CYPs are indicated beside the name of the proteins.

Polyketides, including pigments, antibiotics, and mycotoxins, are a diverse group of secondary metabolites produced by microorganisms and plants. PKSs are complex enzymatic systems for producing polyketides [52-55]. Type I and type II PKSs are modular in structure and contain multiple catalytic activity enzymes individually [56], while the type III PKSs have simple structures [57,58]. There were 10 predicted polyketide synthesis pathways and two predicted hybrid NRPS-PKS synthesis pathways in the KF-25 genome sequence. Twenty-four polyketide synthase genes were extracted from the KF-25 genome and 23 of them were predicted to encode type I PKSs. The remaining gene (KF25_7297) encoded a type III PKS (Figure 6a). Thirteen of 24 predicted PKSs were identified as members of putative secondary metabolism pathways. One such pathway, containing a type I PKS was predicted to produce epothilone (Additional file 1: Table S7). According to previous reports, epothilone is produced by myxobacteria and exhibits anticancer activity by targeting the microtubule of the cancer cell [59]. Because the KF-25 genome contains an epothilone synthesis gene cluster, it is possible that KF-25 might be useful in producing this potential anticancer agent. We will further investigate whether KF-25 produces epothilone and whether the strain has anticancer activity. Type I PKSs have similarity to the type-I fatty acid synthases (FAS), which are essential in lipid metabolism [55,56]. The existence of diverse PKS genes in the *P. chrysogenum* KF-25 genome suggests that KF-25 might produce diverse lipids and polyketides, and that these metabolic products might influence the life cycle of *P. chrysogenum*.

#### Cytochrome P450

Cytochrome P450s (CYPs) are hemoproteins that are ubiquitously distributed throughout all domains of life and play important and diverse roles in metabolic processes and adaptation to different environmental niches by fungi [60]. CYPs participating in numerous primary, secondary, and xenobiotic metabolic reactions have been reported [61,62], and several CYPs predicted from sequenced microorganism genomes were found to be members of secondary metabolism pathways [63,64]. CYPs can be classified into different families based on the amino acid sequences [65,66]. Ninety CYPs were predicted in the KF-25 genome (about 0.9% of total ORFs) and many of them were members of putative secondary metabolism pathways, including the pathways of PKSs, NRPSs, andNRPS-terpenes. These CYPs belonged to 60 different families. There were usually one or two CYPs per family but some families contained three to six CYPs (Figure 6b). The classifications of the CYPs from the Wisconsin 54-1255 genome were almost the same as those from KF-25 genome (Additional file 1: Figure S9). As a multicomponent electron transport chain system, CYPs are critical in degradation, detoxification, and syntheses of life-critical compounds in organisms [67]. Besides their functions in secondary metabolism, CYPs also play critical roles in the adaption of organisms to specific ecological niches and the biosynthesis of physiologically important compounds [68,69]. The existence of so many CYPs might be essential for the life cycle *P. chrysogenum* and the synthesis of the metabolic products, such as penicillin [70].

#### *P. chrysogenum* virus terminal fragment-similar sequences

**Figure 7:**
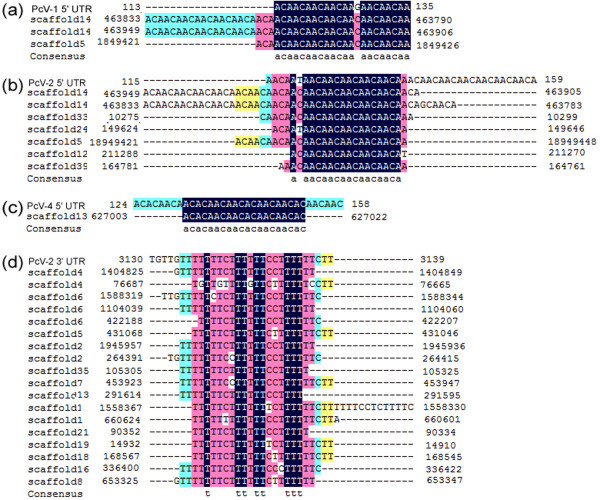
**Sequence alignments of segments from the *P.chrysogenum *KF-25 genome with the 3′-and 5′-UTRs of *P. chrysogenum *virus.** The sequences of three segments from *P. chrysogenum* dsRNA virus are aligned with the genome of KF-25, showing the sequence similarity of the segments from the KF-25 genome to the 3′-and 5′-UTR of the three dsRNA virus segments. Sequences exhibiting similarity to the PcV-1 5′-UTR **(a)**, the PcV-2 5′-UTR **(b)**, the PcV-4 5′-UTR **(c)** and the PcV-2 3′-UTR **(d)** were indicated.

To date, the genome of only one virus originating from *P. chrysogenum* has been sequenced, which showed it was a dsRNA virus of the *Chrysovirus* genus [71,72]. DNA alignment analysis (Figure 7) showed that numerous sequences in the KF-25 genome were similar to the 5′- and 3′-UTR of four *P. chrysogenum* virus DNA sequence segments [72]. These sequences were also found in the genome of Wisconsin 54-1255 (data not shown). The sequences matching the 5′-UTR of the virus were mainly composed of (CAA)_*n*_ repeats, which are similar to the translational enhancer elements in the 5′-UTR of tobacco viruses [73]. Some sequence fragments of KF-25 genome matched the 5′-UTRs and 3′-UTR of virus segment 2, but did not contain regions encoding virus structural proteins. According to previous reports, eukaryotic gneomes contain many sequence of viral origin that have played diverse roles, such as horizontal gene transfer mediated by dsRNA viruses, providing resistance to the virus, and promoting the evolution of host organisms [74-77]. We speculate that *P. chrysogenum* genome might have obtained the UTRs by integrating the viral genome. During the evolutionary process, genes encoding virus structural proteins were eliminated but the UTR regions remained. The functions of these sequences in *P. chrysogenum* genomes are still unknown, but they might provide insertion sites for the virus, or a potential mechanism of viral resistance for *P. chrysogenum*.

## Conclusions

In this study, we reported the genome sequence of wild-type *P. chrysogenum* KF-25*.* This is the second report of a *P. chrysogenum* genome, but the first of wild-type strain*.* Comparative genome analysis showed that KF-25 genome lacked regions of the genome, totaling 2.3 Mb, that were found in Wisconsin 54-1255 genome, which were previously considered to be *P. chrysogenum* species-specific regions [12]*.* However, our results showed that the missing regions were only specific to Wisconsin 54-1255. These regions contained numerous repeat elements and transposable elements, indicating that these segments might have been obtained by Wisconsin 54-1255 through transposition and horizontal gene transfer during evolution. Comparative analysis of KF-25 with another wild-type strain, NRRL 1951, revealed that they had numerous features in common, such as pigments production, and a greater number of pathogenicity- and virulence-associated genes. Based on the phylogenetic tree of 90 conserved orthologous proteins, strains KF-25 and Wisconsin 54-1255 maintained a close evolutionary distance. Analysis of the TCRSs indicated that many proteins were osmolarity TCRSs, which may be an adaptive strategy of *P. chrysogenum* to high osmotic pressure. Several gene clusters involved in putative secondary metabolism pathways, and many genes encoding essential enzymes for the biosynthesis of diverse biologically-active agents were found, which could provide foundation for using *P. chrysogenum* to produce antibiotics including penicillin and other β-lactam antibiotics. The identification of *P. chrysogenum* virus UTR sequences in the two sequenced *P. chrysogenum* genomes is helpful for studying the relationship between the virus and its fungal host in evolution. The results of this study can help us to further understand the genetic diversity of *P. chrysogenum* and shed light on its evolution, biology, environmental adaption and application.

## Methods

### Strains and culture conditions

*P. chrysogenum* strain KF-25 and *U. virens* strain UV-1 were isolated and identified by our lab. Strain Wisconsin 54-1255 [12] was provided by MA van den Berg at DSM Anti-Infectives. Fungal strains were grown in potato-sucrose (PS) medium [20% (w/v) potato lixivium, 2% (w/v) sucrose], and 1.5% (w/v) agar was used in solid potato-sucrose medium (PSA). To assay the antifungal activity, *P. chrysogenum* strains KF-25 and Wisconsin 54-1255 were grown in 500-ml flasks containing 100 ml of PS medium at 28°C for 96 h with shaking (180 rpm). The culture supernatants were filtered through four layers of cheesecloth and centrifugated at 16000 × g for 20 min at 4°C. The culture supernatants were sterilized by filtering through a 0.22 μm membrane (Millipore) and were used to assay the antifungal activity against *U. virens* using the disk diffusion test [78]. The conidia of pathogen *U. virens* were spread on a PSA plate at a density of 10^8^ spores/ml and 100 μl spore suspension was used for each plate, then 20 μl of the sterilized culture supernatant above was added to a piece of sterile filter paper with a 6 mm diameter, placed in the center of the plate. The plate was incubated for 5 days at 28°C. Assays were performed in triplicate.

### HPLC-DAD analysis

Conidiospores of *P. chrysogenum* KF-25 and Wisconsin 54-1255 were inoculated at 10^5^ to 10^6^ conidia/ml in a production medium containing (g/l): glucose · H_2_O, 5; lactose · H_2_O, 80; (NH_2_)_2_CO, 4.5; (NH_4_)_2_SO_4_, 1.1; Na_2_SO_4_, 2.9; KH_2_PO_4_, 5.2; K_2_HPO_4_ · 3H_2_O ,4.8; trace elements solution (citric acid · H_2_O, 150; FeSO_4_ · 7H_2_O,15; MgSO_4_ · 7H_2_O, 150; H_3_BO_3_,0.0075; CuSO_4_ · 5H_2_O, 0.24; CoSO_4_ · 7H_2_O, 0.375; ZnSO_4_ · 7H_2_O, 1.5; MnSO_4_ · H_2_O, 2.28; CaCl_2_ · 2H_2_O, 0.99), 10 (ml/l); 10% phenylacetic acid, pH 7, 75 (ml/l) and the pH was adjusted to pH 6.5 before inoculation [79]. The culture was incubated at 25°C in an orbital shaker at 280 rpm for 4 days. The mycelium was removed by centrifugation and filtration, and the fermentation broth was assayed for penicillin by HPLC-DAD (High Performance Liquid Chromatography-Diode Array Detector). The assay was performed on a Dionex UltiMate 3000 RS HPLC system with autosampler and a DAD detector (Thermo Fisher Scientific, Waltham, MA) and an Agilent ZORBAX 300SB-C18 column (250 × 4.6 mm, 5 μm particle size, Agilent Technologies, Palo Alto, CA). The mobile phase was consisted of solvents A [0.5 mol/L KH_2_PO_4_ (pH3.5): methanol: water, 1:3:6] and B [0.5 mol/L KH_2_PO_4_ (pH3.5): methanol: water, 1:5:4]. The gradient program started with 30% of B, followed by increasing to 100% B from 0 to 20 min, held at 100% B from 20 to 35 min, decreasing to 30% of B from 35 to 50 min. The flow rate was 1.0 ml/min with a column temperature of 25°C. The injection volume was 20 μl, and the detection wavelength was 210 nm. Penicillin G (0.5 mg/ml) was used as a positive control.

The cultures of *P. chrysogenum* KF-25 and Wisconsin 54-1255 in potato-sucrose (PS) medium for 4 days were analysed by HPLC on a Dionex UltiMate 3000 RS HPLC system with autosampler and a DAD detector and a Sepax Polar-Silica column (250 × 10.0 mm, 5 μm particle size, Sepax Technologies, Newark, DE). The mobile phase consisted of solvents A (10 mM ammonium acetate) and B (methanol). The program held at 80% B from 0 to 20 min. The flow rate was 2.0 ml/min and the column temperature was 25°C. The injection volume was 5 μl, and the detection wavelength was 210 nm.

### Genome sequencing, assembly, and annotation

Whole-genome sequencing of KF-25 was performed by the National Center for Gene Research, Shanghai, China. KF-25 genomic DNA was extracted as described previously [80], then was randomly sheared and purifiedto construct three libraries with insert sizes of 170 bp, 500 bp and 2–3 kb. DNA was amplified from the libraries and sequenced by HiSeq2000 (Illumina, California, USA). The reads were assembled into contigs by Velvet (Version 1.2.03) [81] and then scaffolds were constructed based on the contigs using SSPACE [82].

AUGUSTUSugustus ( http://bioinf.uni-greifswald.de/augustus/) [83] was used to predict the genes in the KF-25 genome, and the putative proteins were aligned against the NCBI nr, UniProt ( http://www.uniprot.org/) and KEGG ( http://www.genome.jp/kegg/) database using BLASTP tool ( http://blast.ncbi.nlm.nih.gov/Blast.cgi). The predicted genes were then aligned against the CDD database ( http://www.ncbi.nlm.nih.gov/Structure/cdd/wrpsb.cgi) using rpsBLAST. To identify the KOG classification of each gene, we searched for each amino acid sequence in the KOG database in NCBI using KOGnitor ( http://www.ncbi.nlm.nih.gov/COG/grace/kognitor.html). Metabolic pathways of the KF-25 genome were constructed based on the annotation results against the KEGG database. Repeat sequences were analyzed using CENSOR [84] ( http://www.girinst.org/censor/index.php). The genes encoding tRNA were predicted using tRNAScan [85], and RNAmmer [40] was used to find rDNA sequences. *P. chrysogenum* virus terminal UTR sequences in the KF-25 genome were identified using local BLAST and the sequences were aligned using ClustalX 2.0 [86].

### Comparative genome analysis

Mauve software [87] was used to compare the genome of KF-25 with Wisconsin 54-1255 [GenBank:NS_000201.1]. Dot plot analysis of the two genomes was performed with Gepard [88]. The orthologous genes between KF-25 and Wisconsin54-1255 were by compared the proteomes of the two genomes and proteins that exhibited similarity higher than 25% were thought orthologous. Proteins encoded by all of the strain-specific genes were classified by searching the eukaryotic orthologous groups (KOG) database in NCBI using KOGnitor.

### Detection of strain-specific genes from *P. chrysogenum*

The genomic DNA of KF-25 and Wisconsin 54-1255 was extracted as described previously [80]. Four pairs of primers based on specific gene sequences of Wisconsin 54-1255 (Additional file 1: Table S8) were used to amplify specific genes by PCR (primers used were listed in Additional file 1: Table S8). The products were detected on an agarose gel.

### Secondary metabolism-related gene analysis

The secondary metabolism pathways in the KF-25 and Wisconsin 54-1255 genomes were predicted using antiSMASH ( http://antismash.secondarymetabolites.org/) [29]. Modular polyketide synthases in the genome were predicted and the domain compositions were analyzed using AMSPKS [89] and Pfam [90]. Genome-encoded cytochrome P450s were classified by searching against the fungal cytochrome P450 database [65].

### Phylogenetic analysis

Phylogenetic trees were constructed in MEGA 5.05 [91], using the neighbor-joining method and bootstrap analysis (1,000 replicates), of MUSCLE [92] or ClustalW [86] alignments. Phylogenetic trees of filamentous fungi were constructed as described previously [12] using the aligned amino acid sequences of 90 orthologous genes from *P. chrysogenum* KF-25, *P. chrysogenum* Wisconsin 54-1255 [GenBank:NS_000201.1], *P. marneffei* [GenBank:ABAR00000000], *P. digitatum*[45], *T. stipitatus* [GenBank:ABAS00000000], *A. niger*[93], *A. nidulans* [GenBank:AACD00000000], *A. oryzae*[94], *Aspergillus fumigatus*[95], *Aspergillus clavatus* [GenBank:AAKD00000000], *Aspergillus terreus* [GenBank:AAJN00000000], *Aspergillus flavus* [GenBank:AAIH00000000], *Aspergillus kawachii*[96], *Neosartorya fischeri* [GenBank:AAKE00000000], and *Gibberella zeae* [GenBank:AACM00000000] (Additional file 1: Table S9).

### Data access

The complete genome sequence of *P. chrysogenum* KF-25 has been submitted to SRA ( http://www.ncbi.nlm.nih.gov/sra/) under the accession number SRP022930.

## Competing interests

The authors have no competing interests to declare.

## Authors’ contributions

MYG, QP and YHY designed the study. QP, YHY, XPC and BL performed the experiments and analyzed the data. QP and YHY drafted the manuscript. MYG revised the manuscript. PML, YW and DDW provided reagents. All authors reviewed and approved the final version of the manuscript.

## Supplementary Material

Additional file 1: Table S1 Anticodon usage of Penicillium chrysogenum KF-25 genome. **Figure S1.** Number of occurrences of simple sequence repeats in P. chrysogenum KF-25 genome. **Table S2.** Putative transcription factors in the genome of P. chrysogenum KF-25. **Table S3.** Putative translation factors in the P. chrysogenum KF-25 genome. **Table S4.** List of the ORFs with the predicted function as the compositions of the secretion system. **Figure S2.** Dot plot analysis of P. chrysogenum KF-25 (horizontal) and P. chrysogenum Wisconsin 54–1255 (vertical) genomes. **Table S5.** KOG annotation of the P. chrysogenum Wisconsin 54–1255 specific ORFs. **Figure S3.** Functional classification of the P. chrysogenum Wisconsin 54–1255 and KF-25 specific ORFs based on the KOG database. **Table S6.** KOG annotation of the P. chrysogenum KF-25 specific ORFs. **Figure S4.** Classifications of the origin of the most similar genes in GenBank of the 355 KF-25 specific genes. **Figure S5.** Neighor-Joining phylogenetic tree of P. chrysogenum KF-25 and other species of the genus of penicillium based on the benA gene. **Table S7.** Detail information of the predicted secondary metabolism gene clusters. **Figure S6.** Putative structures of the predicted secondary metabolism gene clusters products. **Figure S7.** Detection of penicillin G by HPLC-DAD. **Figure S8.** The domain compositions and the phylogenetic tree of the non-ribosomal synthetases from KF-25 genome. **Figure S9.** Neighor-Joining (NJ) phylogenetic tree of the cytochrome P450 (CYPs) from the genomes of P. chrysogenum KF-25 and P. chrysogenum Wisconsin 54–1255. **Table S8.** Primers used to amplify the P. chrysogenum Wisconsin 54–1255 specific genes from both the genomes of P. chrysogenum Wisconsin 54–1255 and P. chrysogenum KF-25. **Table S9.** Orthologous genes used in phylogenetic analysis of various filamentous fungi. Click here for file
