# Supplementary material for: Genomic characteristics and comparative genomics analysis of Penicillium chrysogenum KF-25
Source: BMC Genomics. 2014 Feb 21;15:144. doi: 10.1186/1471-2164-15-144 (PMC3938070; doi:10.1186/1471-2164-15-144)
Supplement: Additional file 1: Table S1 — Anticodon usage of Penicillium chrysogenum KF-25 genome. Figure S1. Number of occurrences of simple sequence repeats in P. chrysogenum KF-25 genome. Table S2. Putative transcription factors in the genome of P. chrysogenum KF-25. Table S3. Putative translation factors in the P. chrysogenum KF-25 genome. Table S4. List of the ORFs with the predicted function as the compositions of the secretion system. Figure S2. Dot plot analysis of P. chrysogenum KF-25 (horizontal) and P. chrysogenum Wisconsin 54–1255 (vertical) genomes. Table S5. KOG annotation of the P. chrysogenum Wisconsin 54–1255 specific ORFs. Figure S3. Functional classification of the P. chrysogenum Wisconsin 54–1255 and KF-25 specific ORFs based on the KOG database. Table S6. KOG annotation of the P. chrysogenum KF-25 specific ORFs. Figure S4. Classifications of the origin of the most similar genes in GenBank of the 355 KF-25 specific genes. Figure S5. Neighor-Joining phylogenetic tree of P. chrysogenum KF-25 and other species of the genus of penicillium based on the benA gene. Table S7. Detail information of the predicted secondary metabolism gene clusters. Figure S6. Putative structures of the predicted secondary metabolism gene clusters products. Figure S7. Detection of penicillin G by HPLC-DAD. Figure S8. The domain compositions and the phylogenetic tree of the non-ribosomal synthetases from KF-25 genome. Figure S9. Neighor-Joining (NJ) phylogenetic tree of the cytochrome P450 (CYPs) from the genomes of P. chrysogenum KF-25 and P. chrysogenum Wisconsin 54–1255. Table S8. Primers used to amplify the P. chrysogenum Wisconsin 54–1255 specific genes from both the genomes of P. chrysogenum Wisconsin 54–1255 and P. chrysogenum KF-25. Table S9. Orthologous genes used in phylogenetic analysis of various filamentous fungi. [file 1471-2164-15-144-S1.docx]

**Supplemental Table 1.** **Anticodon usage of** ***Penicillium chrysogenum* KF-25 genome**.

|  | .T. | | .C. | | .A. | | .G. | |  |
| --- | --- | --- | --- | --- | --- | --- | --- | --- | --- |
| T.. | Phe | 0 | Ser | 3 | Tyr | 0 | Cys | 0 | ..T |
|  | Phe | 5 | Ser | 0 | Tyr | 1 | Cys | 3 | ..C |
|  | Leu | 0 | Ser | 0 | Stop | 1 (Sup) | Stop | 2 (Sec) | ..A |
|  | Leu | 0 | Ser | 4 | Stop | 0 | Trp | 0 | ..G |
| C.. | Leu | 5 | Pro | 0 | His | 0 | Arg | 7 | ..T |
|  | Leu | 0 | Pro | 0 | His | 4 | Arg | 0 | ..C |
|  | Leu | 0 | Pro | 0 | Gln | 2 | Arg | 1 | ..A |
|  | Leu | 1 | Pro | 1 | Gln | 3 | Arg | 0 | ..G |
| A.. | Ile | 11 | Thr | 2 | Asn | 0 | Ser | 0 | ..T |
|  | Ile | 1 | Thr | 0 | Asn | 2 | Ser | 3 | ..C |
|  | Ile | 1 | Thr | 3 | Lys | 1 | Arg | 1 | ..A |
|  | Met | 4 | Thr | 1 | Lys | 4 | Arg | 1 | ..G |
| G.. | Val | 5 | Ala | 3 | Asp | 0 | Gly | 0 | ..T |
|  | Val | 0 | Ala | 0 | Asp | 4 | Gly | 7 | ..C |
|  | Val | 1 | Ala | 1 | Glu | 1 | Gly | 2 | ..A |
|  | Val | 0 | Ala | 2 | Glu | 6 | Gly | 0 | ..G |

Totally, 112 genes encoded tRNA were identified. The number in the table indicated the number of tRNAs identified. No tRNA for Trp was identified and two additional tRNA genes have been identified for the specificity of which is unknown. Two genes encoded selenocysteine (Sec) were identified and one gene encoded Sup was identified.


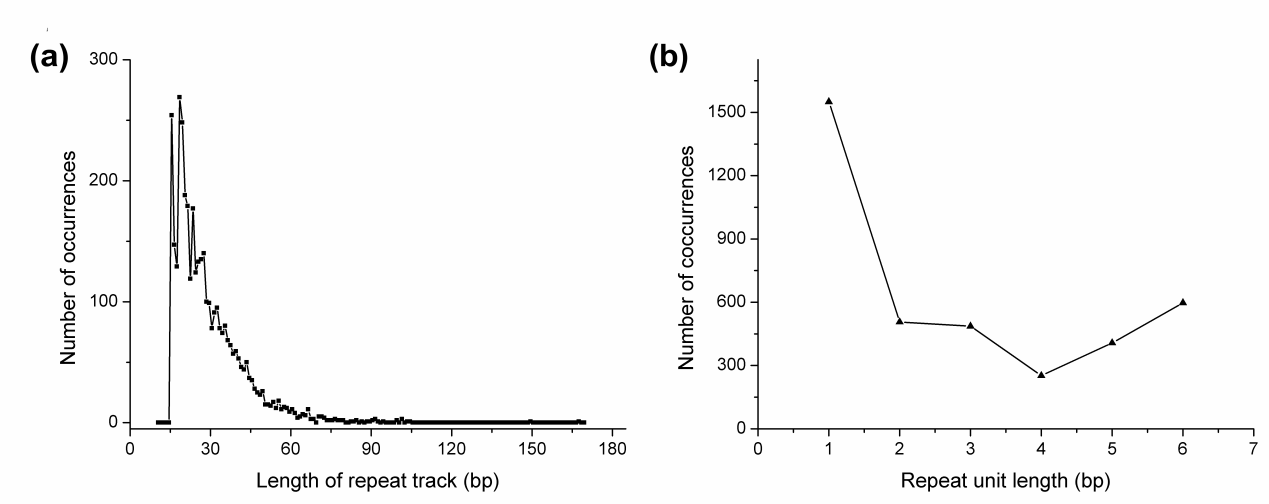


**Supplemental Figure 1. Number of occurrences of simple sequence repeats in *P. chrysogenum* KF-25 genome.** The numbers of the repeats were classified by the length of tracts and the length repeat units. Totally, 3798 repeats were found in the genome of *P. chrysogenum* KF-25 and the lengths of them were mainly from 15bp to 60bp. Most of these repeats were repeats for mononucleotides or repeats for six nucleotides.

**Supplemental Table 2. Putative transcription factors in the genome of *P. chrysogenum* KF-25.**

| **ORFs** | **Putative protein function** |
| --- | --- |
| KF25_0055 KF25_0199 | bZIP factor, other  Forkhead transcription factor HCM1 |
| KF25_0292 | regulatory factor X, other |
| KF25_0404 | transcriptional regulatory protein GAL4 |
| KF25_0430 | MADS-box transcription enhancer factor 2D |
| KF25_0575 | krueppel-like factor 10/11 |
| KF25_0625 | sterol regulatory element-binding transcription factor 1 |
| KF25_0748 | transcriptional regulatory protein LEU3 |
| KF25_1074 | arginine metabolism regulation protein II |
| KF25_1089 | myb proto-oncogene protein |
| KF25_1100 | krueppel-like factor 10/11 |
| KF25_1185 | zinc finger and BTB domain-containing protein 7 |
| KF25_1190 | GATA-binding protein, other eukaryote |
| KF25_1204 | minichromosome maintenance protein 1 |
| KF25_1223 | transcription factor YY |
| KF25_1323 | early growth response protein 3 |
| KF25_1618 | arginine metabolism regulation protein II |
| KF25_1764 | proline utilization trans-activator |
| KF25_1825 | arginine metabolism regulation protein II |
| KF25_1915 | TEA domain family member 1/3/4 |
| KF25_2158 | proline utilization trans-activator |
| KF25_2437 | krueppel-like factor 6/7 |
| KF25_2591 | general control protein GCN4 |
| KF25_2613 | zinc finger protein GLI |
| KF25_2690 | HMG box factor, other |
| KF25_2921 | COMPASS component SPP1 |
| KF25_2932 | homeobox protein YOX1/YHP1 |
| KF25_2961 | Myb-like DNA-binding protein REB1 |
| KF25_3017 | transcription factor Sp4 |
| KF25_3168 | early growth response protein 1 |
| KF25_3197 | arginine metabolism regulation protein II |
| KF25_3322 | early growth response protein 1 |
| KF25_3335 | transcription factor Sp1 |
| KF25_3371 | GATA-binding protein, other eukaryote |
| KF25_3446 | proline utilization trans-activator |
| KF25_3777 | nuclear transcription Y subunit beta |
| KF25_3955 | proline utilization trans-activator |
| KF25_4095 | transcriptional regulatory protein LEU3 |
| KF25_4116 | transcriptional regulatory protein UME6 |
| KF25_4139 | forkhead box protein G |
| KF25_4186 | transcription factor Sp4 |
| KF25_4242 | transcriptional regulator |
| KF25_4326 | transcriptional regulator |
| KF25_4396 | zinc finger protein GLI |
| KF25_4404 | arginine metabolism regulation protein II |
| KF25_4674 | homeobox protein cut-like |
| KF25_4908 | KRAB domain-containing zinc finger protein |
| KF25_5042 | nuclear transcription factor Y, gamma |
| KF25_5189 | activating transcription factor 2 |
| KF25_5282 | early growth response protein 3 |
| KF25_5332 | krueppel-like factor 5 |
| KF25_5337 | arginine metabolism regulation protein II |
| KF25_5411 | GATA-binding protein, other eukaryote |
| KF25_5466 | transcription factor Sp3 |
| KF25_5517 | heme activator protein 1 |
| KF25_6015 | transcriptional regulatory protein CAT8 |
| KF25_6416 | replication factor A1 |
| KF25_6455 | cellular nucleic acid-binding protein |
| KF25_6584 | transcriptional regulatory protein GAL4 |
| KF25_6718 | nuclear transcription factor Y, alpha |
| KF25_6793 | proline utilization trans-activator |
| KF25_6886 | cellular nucleic acid-binding protein |
| KF25_6951 | structure-specific recognition protein 1 |
| KF25_7015 | regulatory protein SWI6 |
| KF25_7044 | transcription factor STE12 |
| KF25_7360 | transcription factor CP2 and related proteins |
| KF25_7365 | metallothionein expression activator |
| KF25_7373 | activating transcription factor 7 |
| KF25_7460 | transcriptional regulatory protein GAL4 |
| KF25_7467 | transcription initiation factor TFIID TATA-box-binding protein |
| KF25_7478 | forkhead box protein K |
| KF25_7548 | zinc-finger protein CreA/MIG |
| KF25_7681 | proline utilization trans-activator |
| KF25_7868 | phosphate system cyclin PHO80 |
| KF25_7941 | regulatory protein PHO2 |
| KF25_7958 | KRAB and SCAN domains-containing zinc finger protein |
| KF25_8176 | bHLH factor, other |
| KF25_8448 | transcriptional regulatory protein CAT8 |
| KF25_8572 | transcription factor CP2 and related proteins |
| KF25_8632 | phosphate system cyclin PHO80 |
| KF25_8780 | heat shock transcription factor 4 |
| KF25_8846 | early growth response protein 1 |
| KF25_9049 | heat shock transcription factor, other eukaryote |
| KF25_9116 | transcriptional regulatory protein CAT8 |
| KF25_9266 | zinc finger and BTB domain-containing protein 7 |
| KF25_9357 | zinc finger protein GLI |
| KF25_9386 | early growth response protein 1 |
| KF25_9420 | AraC family transcriptional regulator, regulatory protein of adaptative response / methylphosphotriester-DNA alkyltransferase methyltransferase |
| KF25_9504 | fungal AP-1-like factor |
| KF25_9622 | transcriptional regulatory protein GAL4 |
| KF25_9657 | nuclear transcription factor Y, gamma |

**Supplemtental Table 3.** **Putative translation factors in the *P. chrysogenum* KF-25 genome.**

| **ORFs Putative gene function** | **Putative protein function** |
| --- | --- |
| KF25_0157 | ATP-binding cassette, subfamily F, member 3 |
| KF25_0241 | translation initiation factor 6 |
| KF25_0447 | translation initiation factor 2 subunit 2 |
| KF25_0452 | translation initiation factor 3 subunit I |
| KF25_0693 | translation initiation factor 4A |
| KF25_0745 | translation initiation factor 1 |
| KF25_0793 | translation initiation factor 2A |
| KF25_1050 | translation initiation factor 5 |
| KF25_1241 | translation initiation factor 1A |
| KF25_1371 | translation initiation factor 3 subunit B |
| KF25_1508 | translation initiation factor 3 subunit L |
| KF25_1566 | translation initiation factor 3 subunit G |
| KF25_1627 | translation initiation factor 4G |
| KF25_1643 | translation initiation factor 2 subunit 3 |
| KF25_1851 | translation initiation factor 3 subunit A |
| KF25_2018 | translation initiation factor 5A |
| KF25_2023 | translation initiation factor 5B |
| KF25_2479 | translation initiation factor eIF-2B subunit epsilon |
| KF25_2885 | protein TIF31 |
| KF25_2981 | translation initiation factor 3 subunit K |
| KF25_3868 | translation initiation factor IF-2 |
| KF25_4399 | translation initiation factor eIF-2B subunit gamma |
| KF25_4697 | diphthine synthase |
| KF25_4806 | translation initiation factor 3 subunit F |
| KF25_4819 | translation initiation factor eIF-2B subunit beta |
| KF25_4976 | translation initiation factor 3 subunit H |
| KF25_5210 | translation initiation factor eIF-2B subunit delta |
| KF25_5373 | translation initiation factor eIF-2B subunit alpha |
| KF25_5475 | translation initiation factor 3 subunit M |
| KF25_5709 | translation initiation factor eIF-2B subunit gamma |
| KF25_5717 | translation initiation factor 4B |
| KF25_5965 | translation initiation factor 2D |
| KF25_6570 | translation initiation factor 4E |
| KF25_6723 | translation initiation factor 3 subunit D |
| KF25_7098 | translation initiation factor eIF-2B subunit gamma |
| KF25_7376 | translation initiation factor 3 subunit E |
| KF25_8753 | translation initiation factor 3 subunit C |
| KF25_8905 | translation initiation factor 4E |
| KF25_8991 | ATP-dependent RNA helicase |

**Supplemental Table 4. List of the ORFs with the predicted function as the compositions of the secretion system.** The proteins encoded by KF-25 genome that were predicted as the compositions of the eukaryotic Sec-SRP secretion systems were list and the function of the corresponding gene functions were indicated.

| **ORFs** | **Putative gene function** |
| --- | --- |
| KF25_0388 | protein transport protein SEC61 subunit gamma and related proteins |
| KF25_0907 | signal recognition particle receptor subunit beta |
| KF25_2026 | signal recognition particle subunit SRP68 |
| KF25_4540 | translocation protein SEC63 |
| KF25_6227 | signal recognition particle subunit SRP19 |
| KF25_6387 | translocation protein SEC66 |
| KF25_7604 | signal recognition particle receptor subunit alpha |
| KF25_7956 | signal recognition particle subunit SRP72 |
| KF25_8024 | signal recognition particle subunit SRP54 |
| KF25_8100 | translocation protein SEC72 |
| KF25_8186 | protein transport protein SEC61 subunit alpha |
| KF25_9163 | translocation protein SEC62 |


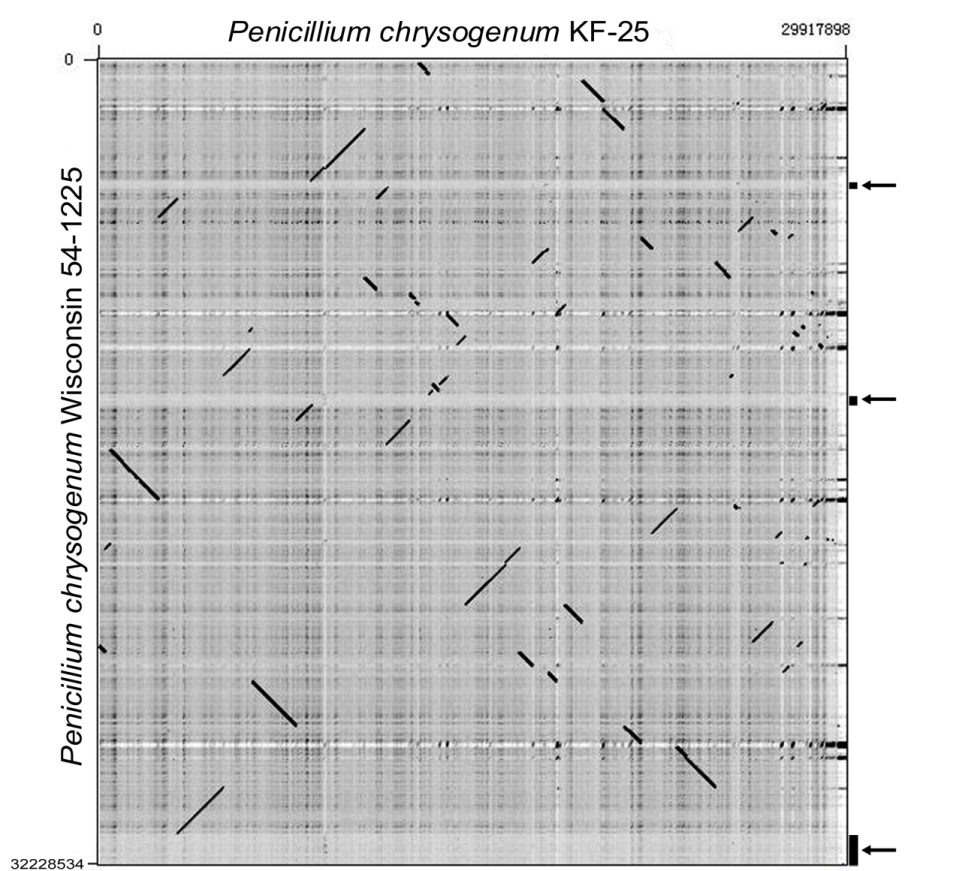


**Supplemental Figure 2. Dot plot analysis of *P. chrysogenum* KF-25 (horizontal) and *P. chrysogenum* Wisconsin 54-1255 (vertical) genomes.** Axes represented the concatenation of all chromosomes for corresponding genome. The three black rectangle on the right with the arrow pointed at were the main deletion DNA regions of KF-25 genome compared with the Wisconsin 54-1255 genome.

**Supplemental Table 5. KOG annotation of the *P. chrysogenum* Wisconsin 54-1255 specific ORFs.** Totally, 2317 *P. chrysogenum* Wisconsin 54-1255 specific ORFs were found and 146 ORFs with the orthologs proteins in KOG database were listed.

| orfs | Genbank number | KOG number | KOG annotation |
| --- | --- | --- | --- |
| Pc22g18120 | XP_002565720.1 | KOG1572 | Predicted protein tyrosine phosphatase |
| Pc22g03350 | XP_002564378.1 | KOG3342 | Signal peptidase I |
| Pc20g05650 | XP_002563099.1 | KOG2233 | Alpha-N-acetylglucosaminidase |
| Pc15g01520 | XP_002560372.1 | KOG4076 | Regulator of ATP-sensitive K+ channels Alpha-endosulfine/ARPP-19 and related cAMP-regulated phosphoproteins |
| hypothetical protein | XP_002556686.1 | KOG2268 | Serine/threonine protein kinase |
| Pc13g01040 | XP_002558551.1 | KOG1290 | Serine/threonine protein kinase |
| Pc20g01000 | XP_002562661.1 | KOG2064 | Poly(ADP-ribose) glycohydrolase |
| Pc24g01050 | XP_002566761.1 | KOG0578 | p21-activated serine/threonine protein kinase |
| Pc12g01390 | XP_002557037.1 | KOG0665 | Jun-N-terminal kinase (JNK) |
| Pc24g01590 | XP_002566808.1 | KOG0665 | Jun-N-terminal kinase (JNK) |
| Pc21g19500 | XP_002568941.1 | KOG1705 | Uncharacterized conserved protein, contains CXXC motifs |
| Pc22g11520 | XP_002565099.1 | KOG4489 | Uncharacterized conserved protein BC10 (implicated in bladder cancer in humans) |
| Pc09g00080 | XP_002556875.1 | KOG2029 | Uncharacterized conserved protein |
| Pc13g00100 | XP_002558464.1 | KOG4781 | Uncharacterized conserved protein |
| Pc13g00190 | XP_002558473.1 | KOG2029 | Uncharacterized conserved protein |
| Pc13g00980 | XP_002558545.1 | KOG2029 | Uncharacterized conserved protein |
| Pc13g11270 | XP_002559545.1 | KOG4781 | Uncharacterized conserved protein |
| Pc13g15200 | XP_002559916.1 | KOG4533 | Uncharacterized conserved protein |
| Pc14g01280 | XP_002560122.1 | KOG4620 | Uncharacterized conserved protein |
| Pc16g02520 | XP_002560624.1 | KOG3808 | Uncharacterized conserved protein |
| Pc20g00400 | XP_002562603.1 | KOG3444 | Uncharacterized conserved protein |
| Pc22g15130 | XP_002565431.1 | KOG2029 | Uncharacterized conserved protein |
| Pc22g16980 | XP_002565611.1 | KOG4488 | Small EDRK-rich protein H4F5 |
| Pc16g09500 | XP_002561264.1 | KOG2816 | Predicted transporter ADD1 (major facilitator superfamily) |
| Pc16g13820 | XP_002561681.1 | KOG1611 | Predicted short chain-type dehydrogenase |
| Pc06g02340 | XP_002556836.1 | KOG4300 | Predicted methyltransferase |
| Pc22g26680 | XP_002566534.1 | KOG2968 | Predicted esterase of the alpha-beta hydrolase superfamily (Neuropathy target esterase), contains cAMP-binding domains |
| Pc42g00010 | XP_002566961.1 | KOG2968 | Predicted esterase of the alpha-beta hydrolase superfamily (Neuropathy target esterase), contains cAMP-binding domains |
| Pc43g00010 | XP_002566962.1 | KOG2968 | Predicted esterase of the alpha-beta hydrolase superfamily (Neuropathy target esterase), contains cAMP-binding domains |
| Pc16g02550 | XP_002560627.1 | KOG3116 | Predicted C3H1-type Zn-finger protein |
| Pc06g02240 | XP_002556826.1 | KOG0370 | Multifunctional pyrimidine synthesis protein CAD (includes carbamoyl-phophate synthetase, aspartate transcarbamylase, and glutamine amidotransferase) |
| Pc16g14920 | XP_002561789.1 | KOG1721 | FOG: Zn-finger |
| Pc04g00010 | XP_002556598.1 | KOG1721 | FOG: Zn-finger |
| Pc06g01210 | XP_002556728.1 | KOG1721 | FOG: Zn-finger |
| hypothetical protein | XP_002556922.1 | KOG1721 | FOG: Zn-finger |
| Pc12g08150 | XP_002557647.1 | KOG1721 | FOG: Zn-finger |
| Pc12g09540 | XP_002557781.1 | KOG1721 | FOG: Zn-finger |
| hypothetical protein | XP_002558555.1 | KOG1721 | FOG: Zn-finger |
| Pc22g15230 | XP_002565441.1 | KOG1721 | FOG: Zn-finger |
| Pc22g26080 | XP_002566482.1 | KOG1721 | FOG: Zn-finger |
| Pc23g00400 | XP_002566603.1 | KOG1721 | FOG: Zn-finger |
| Pc24g00420 | XP_002566702.1 | KOG1721 | FOG: Zn-finger |
| Pc24g00840 | XP_002566740.1 | KOG1721 | FOG: Zn-finger |
| Pc24g01490 | XP_002566799.1 | KOG1721 | FOG: Zn-finger |
| Pc24g01720 | XP_002566820.1 | KOG1721 | FOG: Zn-finger |
| Pc24g02600 | XP_002566893.1 | KOG1721 | FOG: Zn-finger |
| Pc17g00170 | XP_002566980.1 | KOG1721 | FOG: Zn-finger |
| Pc21g22480 | XP_002569217.1 | KOG1721 | FOG: Zn-finger |
| Pc06g00940 | XP_002556703.1 | KOG0017 | FOG: Transposon-encoded proteins with TYA, reverse transcriptase, integrase domains in various combinations |
| Pc12g03600 | XP_002557243.1 | KOG0017 | FOG: Transposon-encoded proteins with TYA, reverse transcriptase, integrase domains in various combinations |
| Pc12g07890 | XP_002557623.1 | KOG0017 | FOG: Transposon-encoded proteins with TYA, reverse transcriptase, integrase domains in various combinations |
| Pc13g02140 | XP_002558657.1 | KOG0017 | FOG: Transposon-encoded proteins with TYA, reverse transcriptase, integrase domains in various combinations |
| Pc13g03100 | XP_002558748.1 | KOG0017 | FOG: Transposon-encoded proteins with TYA, reverse transcriptase, integrase domains in various combinations |
| Pc16g06790 | XP_002561013.1 | KOG0017 | FOG: Transposon-encoded proteins with TYA, reverse transcriptase, integrase domains in various combinations |
| Pc16g06850 | XP_002561019.1 | KOG0017 | FOG: Transposon-encoded proteins with TYA, reverse transcriptase, integrase domains in various combinations |
| Pc16g15070 | XP_002561801.1 | KOG0017 | FOG: Transposon-encoded proteins with TYA, reverse transcriptase, integrase domains in various combinations |
| Pc18g00670 | XP_002561914.1 | KOG0017 | FOG: Transposon-encoded proteins with TYA, reverse transcriptase, integrase domains in various combinations |
| Pc21g03060 | XP_002567370.1 | KOG0017 | FOG: Transposon-encoded proteins with TYA, reverse transcriptase, integrase domains in various combinations |
| Pc21g08820 | XP_002567921.1 | KOG0017 | FOG: Transposon-encoded proteins with TYA, reverse transcriptase, integrase domains in various combinations |
| Pc21g09890 | XP_002568025.1 | KOG0017 | FOG: Transposon-encoded proteins with TYA, reverse transcriptase, integrase domains in various combinations |
| Pc21g10180 | XP_002568053.1 | KOG0017 | FOG: Transposon-encoded proteins with TYA, reverse transcriptase, integrase domains in various combinations |
| Pc21g17160 | XP_002568714.1 | KOG0017 | FOG: Transposon-encoded proteins with TYA, reverse transcriptase, integrase domains in various combinations |
| Pc21g17210 | XP_002568719.1 | KOG0017 | FOG: Transposon-encoded proteins with TYA, reverse transcriptase, integrase domains in various combinations |
| Pc21g20610 | XP_002569048.1 | KOG0017 | FOG: Transposon-encoded proteins with TYA, reverse transcriptase, integrase domains in various combinations |
| Pc21g23020 | XP_002569269.1 | KOG0017 | FOG: Transposon-encoded proteins with TYA, reverse transcriptase, integrase domains in various combinations |
| Pc13g00050 | XP_002558460.1 | KOG1075 | FOG: Reverse transcriptase |
| Pc16g09090 | XP_002561228.1 | KOG1075 | FOG: Reverse transcriptase |
| Pc20g14870 | XP_002563962.1 | KOG1075 | FOG: Reverse transcriptase |
| Pc22g26460 | XP_002566512.1 | KOG0504 | FOG: Ankyrin repeat |
| Pc23g00230 | XP_002566591.1 | KOG0504 | FOG: Ankyrin repeat |
| Pc24g01800 | XP_002566828.1 | KOG0504 | FOG: Ankyrin repeat |
| Pc24g02440 | XP_002566881.1 | KOG0504 | FOG: Ankyrin repeat |
| Pc24g02810 | XP_002566909.1 | KOG0504 | FOG: Ankyrin repeat |
| Pc17g01160 | XP_002567064.1 | KOG0504 | FOG: Ankyrin repeat |
| Pc21g22430 | XP_002569213.1 | KOG1516 | Carboxylesterase and related proteins |
| Pc22g26470 | XP_002566513.1 | KOG0509 | Ankyrin repeat and DHHC-type Zn-finger domain containing proteins |
| Pc24g01190 | XP_002566772.1 | KOG0509 | Ankyrin repeat and DHHC-type Zn-finger domain containing proteins |
| Pc24g02340 | XP_002566871.1 | KOG0509 | Ankyrin repeat and DHHC-type Zn-finger domain containing proteins |
| Pc24g02430 | XP_002566880.1 | KOG0509 | Ankyrin repeat and DHHC-type Zn-finger domain containing proteins |
| Pc21g08520 | XP_002567891.1 | KOG0054 | Multidrug resistance-associated protein/mitoxantrone resistance protein, ABC superfamily |
| Pc21g12370 | XP_002568267.1 | KOG0054 | Multidrug resistance-associated protein/mitoxantrone resistance protein, ABC superfamily |
| Pc13g00880 | XP_002558535.1 | KOG1263 | Multicopper oxidases |
| Pc06g01120 | XP_002556721.1 | KOG0022 | Alcohol dehydrogenase, class III |
| Pc16g00480 | XP_002560470.1 | KOG0022 | Alcohol dehydrogenase, class III |
| Pc13g00960 | XP_002558543.1 | KOG0039 | Ferric reductase, NADH/NADPH oxidase and related proteins |
| Pc22g00640 | XP_002564109.1 | KOG0803 | Predicted E3 ubiquitin ligase |
| Pc21g21630 | XP_002569135.1 | KOG4628 | Predicted E3 ubiquitin ligase |
| Pc23g00270 | XP_002566594.1 | KOG0019 | Molecular chaperone (HSP90 family) |
| hypothetical protein | XP_002558497.1 | KOG0800 | FOG: Predicted E3 ubiquitin ligase |
| Pc18g00180 | XP_002561865.1 | KOG0800 | FOG: Predicted E3 ubiquitin ligase |
| Pc21g17330 | XP_002568731.1 | KOG4114 | Cytochrome c oxidase assembly protein PET191 |
| Pc04g00030 | XP_002556600.1 | KOG1121 | Tam3-transposase (Ac family) |
| Pc24g00890 | XP_002566745.1 | KOG1121 | Tam3-transposase (Ac family) |
| Pc24g01370 | XP_002566787.1 | KOG1121 | Tam3-transposase (Ac family) |
| Pc24g02380 | XP_002566875.1 | KOG1121 | Tam3-transposase (Ac family) |
| Pc24g02930 | XP_002566919.1 | KOG1121 | Tam3-transposase (Ac family) |
| Pc21g00480 | XP_002567122.1 | KOG1121 | Tam3-transposase (Ac family) |
| Pc09g00030 | XP_002556870.1 | KOG2248 | 3'-5' exonuclease |
| Pc22g26790 | XP_002566545.1 | KOG1414 | Transcriptional activator FOSB/c-Fos and related bZIP transcription factors |
| Pc23g00970 | XP_002566656.1 | KOG0260 | RNA polymerase II, large subunit |
| Pc22g04170 | XP_002564456.1 | KOG2907 | RNA polymerase I transcription factor TFIIS, subunit A12.2/RPA12 |
| Pc22g09130 | XP_002564925.1 | KOG2907 | RNA polymerase I transcription factor TFIIS, subunit A12.2/RPA12 |
| Pc06g02230 | XP_002556825.1 | KOG0014 | MADS box transcription factor |
| Pc13g00970 | XP_002558544.1 | KOG0014 | MADS box transcription factor |
| Pc23g00060 | XP_002566576.1 | KOG0014 | MADS box transcription factor |
| Pc17g00020 | XP_002566970.1 | KOG0014 | MADS box transcription factor |
| Pc22g26380 | XP_002566506.1 | KOG0627 | Heat shock transcription factor |
| Pc24g00580 | XP_002566715.1 | KOG0627 | Heat shock transcription factor |
| Pc17g00880 | XP_002567037.1 | KOG0627 | Heat shock transcription factor |
| Pc12g09220 | XP_002557749.1 | KOG1601 | GATA-4/5/6 transcription factors |
| transcription factor rfeH | XP_002567427.1 | KOG1601 | GATA-4/5/6 transcription factors |
| Pc13g03870 | XP_002558823.1 | KOG0009 | Ubiquitin-like/40S ribosomal S30 protein fusion |
| Pc22g19050 | XP_002565808.1 | KOG3311 | Ribosomal protein S18 |
| Pc12g15770 | XP_002558376.1 | KOG3429 | Predicted peptidyl-tRNA hydrolase |
| Pc16g07630 | XP_002561088.1 | KOG1147 | Glutamyl-tRNA synthetase |
| Pc22g25660 | XP_002566451.1 | KOG3504 | 60S ribosomal protein L29 |
| Pc22g01560 | XP_002564200.1 | KOG3506 | 40S ribosomal protein S29 |
| Pc21g22160 | XP_002569186.1 | KOG1269 | SAM-dependent methyltransferases |
| Pc21g22400 | XP_002569210.1 | KOG4178 | Soluble epoxide hydrolase |
| Pc14g02390 | XP_002560230.1 | KOG4231 | Intracellular membrane-bound Ca2+-independent phospholipase A2 |
| Pc22g26670 | XP_002566533.1 | KOG4231 | Intracellular membrane-bound Ca2+-independent phospholipase A2 |
| Pc23g00010 | XP_002566571.1 | KOG4231 | Intracellular membrane-bound Ca2+-independent phospholipase A2 |
| Pc24g03120 | XP_002566928.1 | KOG4231 | Intracellular membrane-bound Ca2+-independent phospholipase A2 |
| Pc27g00010 | XP_002566946.1 | KOG4231 | Intracellular membrane-bound Ca2+-independent phospholipase A2 |
| Pc17g01040 | XP_002567053.1 | KOG4231 | Intracellular membrane-bound Ca2+-independent phospholipase A2 |
| Pc21g00340 | XP_002567109.1 | KOG4231 | Intracellular membrane-bound Ca2+-independent phospholipase A2 |
| Pc21g15720 | XP_002568582.1 | KOG2329 | Alkaline ceramidase |
| Pc21g02400 | XP_002567304.1 | KOG3855 | Monooxygenase involved in coenzyme Q (ubiquinone) biosynthesis |
| Pc20g03380 | XP_002562889.1 | KOG2371 | Molybdopterin biosynthesis protein |
| Pc17g00810 | XP_002567030.1 | KOG1460 | GDP-mannose pyrophosphorylase |
| Pc22g05000 | XP_002564537.1 | KOG2499 | Beta-N-acetylhexosaminidase |
| Pc20g03670 | XP_002562918.1 | KOG0223 | Aquaporin (major intrinsic protein family) |
| Pc12g14470 | XP_002558252.1 | KOG2466 | Uridine permease/thiamine transporter/allantoin transport |
| Pc16g06760 | XP_002561010.1 | KOG1018 | Cytosine deaminase FCY1 and related enzymes |
| Pc23g00640 | XP_002566623.1 | KOG1250 | Threonine/serine dehydratases |
| Pc22g01010 | XP_002564146.1 | KOG3124 | Pyrroline-5-carboxylate reductase |
| Pc21g03420 | XP_002567406.1 | KOG0588 | Serine/threonine protein kinase |
| Pc13g00530 | XP_002558502.1 | KOG0987 | DNA helicase PIF1/RRM3 |
| Pc17g00330 | XP_002566995.1 | KOG0987 | DNA helicase PIF1/RRM3 |
| Pc21g22460 | XP_002569215.1 | KOG2614 | Kynurenine 3-monooxygenase and related flavoprotein monooxygenases |
| Pc13g01940 | XP_002558638.1 | KOG0535 | Sulfite oxidase, molybdopterin-binding component |
| Pc20g03360 | XP_002562888.1 | KOG2282 | NADH-ubiquinone oxidoreductase, NDUFS1/75 kDa subunit |
| Pc24g00480 | XP_002566708.1 | KOG2132 | Uncharacterized conserved protein, contains JmjC domain |
| Pc13g00650 | XP_002558512.1 | KOG3105 | DNA-binding centromere protein B (CENP-B) |
| Pc14g00870 | XP_002560081.1 | KOG1783 | Small nuclear ribonucleoprotein F |
| Pc22g13990 | XP_002565323.1 | KOG3503 | H/ACA snoRNP complex, subunit NOP10 |


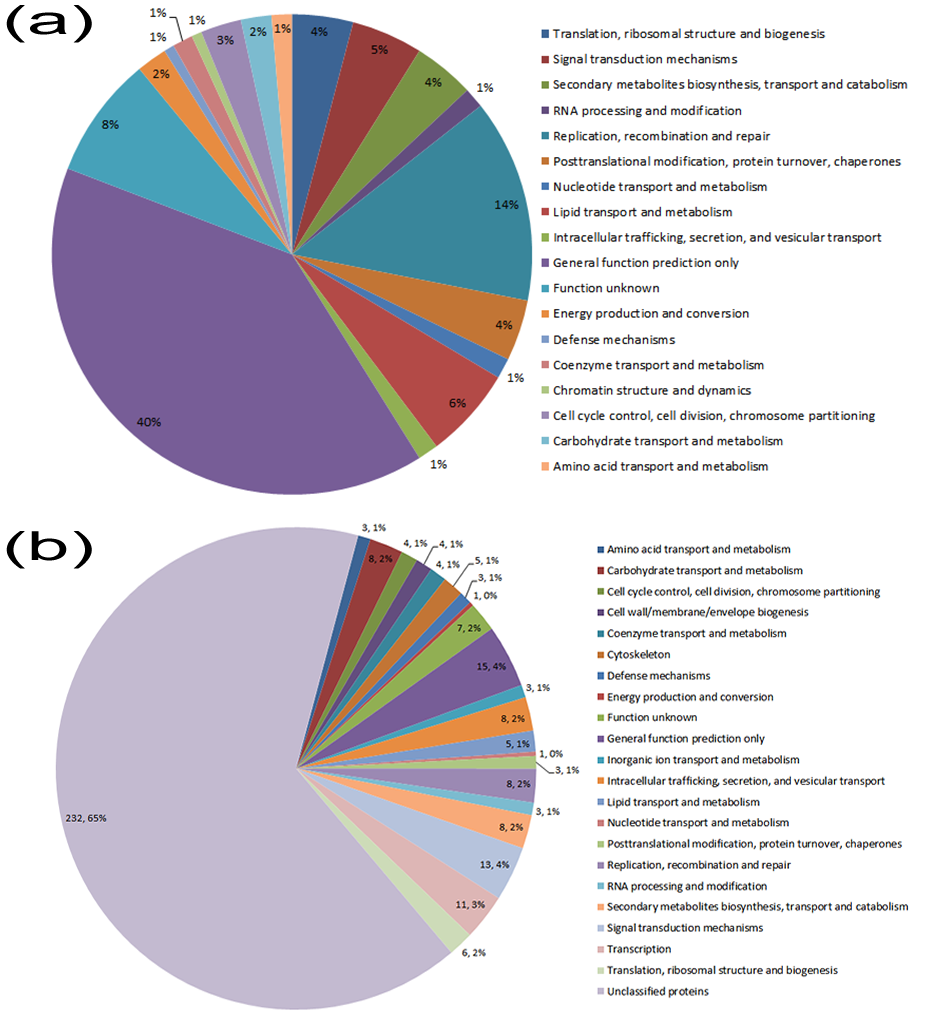


**Supplemental Figure 3. Functional classification of the *P. chrysogenum* Wisconsin 54-1255 and KF-25 specific ORFs based on the KOG (Eukaryotic Orthologous Groups of proteins) database.** (a)Totally, 2,317 *P. chrysogenum* Wisconsin 54-1255 specific ORFs searched against the KOG database and 146 ORFs with the orthologs proteins in KOG database were classified. (b) Totally, 355 *P. chrysogenum* KF-25 specific ORFs searched against the KOG database and 123 ORFs with the orthologs proteins in KOG database were classified.

**Supplemental Table 6.** **KOG annotation of the *P. chrysogenum* KF-25 specific ORFs.** Totally, 355 *P. chrysogenum* KF-25 specific ORFs were found and 123 ORFs with the orthologs proteins in KOG database were listed.

| ORFs | KOG number | KOG annotation | KOG classification | Best hits | Genebank number of best hits |
| --- | --- | --- | --- | --- | --- |
| KF25_0009 | KOG3276 | Uncharacterized conserved protein, contains YggU domain | Function unknown | *Aspergillus fumigatus* A1163 | XP_001481499.1 |
| KF25_0047 | KOG0704 | ADP-ribosylation factor GTPase activator | Signal transduction mechanisms | *Aspergillus niger* CBS 513.88 | XP_001398269.2 |
| KF25_0096 | KOG1239 | Inner membrane protein translocase involved in respiratory chain assembly | Posttranslational modification, protein turnover, chaperones | *Aspergillus clavatus* NRRL 1 | XP_001276169.1 |
| KF25_0123 | KOG3271 | Translation initiation factor 5A (eIF-5A) | Translation, ribosomal structure and biogenesis | *Aspergillus terreus* NIH2624 | XP_001215759.1 |
| KF25_0140 | KOG1315 | Predicted DHHC-type Zn-finger protein | General function prediction only | *Aspergillus terreus* NIH2624 | XP_001215771.1 |
| KF25_0242 | KOG1924 | RhoA GTPase effector DIA/Diaphanous | Signal transduction mechanisms | *Aspergillus kawachii* IFO 4308 | GAA89886.1 |
| KF25_0246 | KOG1724 | SCF ubiquitin ligase, Skp1 component | Posttranslational modification, protein turnover, chaperones | *Arthroderma otae* CBS 113480 | XP_002848814.1 |
| KF25_0500 | KOG2518 | 5'-3' exonuclease | Replication, recombination and repair | *Aspergillus oryzae* RIB40 | XP_001818292.1 |
| KF25_0541 | KOG4062 | 6-O-methylguanine-DNA methyltransferase MGMT/MGT1, involved in DNA repair | Replication, recombination and repair | *Aspergillus fumigatus* A1163 | XP_749344.1 |
| KF25_0587 | KOG4177 | Ankyrin | Cell wall/membrane/envelope biogenesis | *Uncinocarpus reesii* 1704 | XP_002541759.1 |
| KF25_0609 | KOG3770 | Acid sphingomyelinase and PHM5 phosphate metabolism protein | Lipid transport and metabolism | *Neosartorya fischeri* NRRL 181 | XP_001265721.1 |
| KF25_0754 | KOG2689 | Predicted ubiquitin regulatory protein | Posttranslational modification, protein turnover, chaperones | *Neosartorya fischeri* NRRL 181 | XP_001263817.1 |
| KF25_0758 | KOG1337 | N-methyltransferase | General function prediction only | *Aspergillus clavatus* NRRL 1 | XP_001270658.1 |
| KF25_1190 | KOG1601 | GATA-4/5/6 transcription factors | Transcription | *Penicillium roqueforti* | O13508.2 |
| KF25_1319 | KOG1202 | Animal-type fatty acid synthase and related proteins | Lipid transport and metabolism | *Aspergillus nidulans* FGSC A4 | XP_681652.1 |
| KF25_1323 | KOG2462 | C2H2-type Zn-finger protein | Transcription | *Aspergillus clavatus* NRRL 1 | XP_001270229.1 |
| KF25_1494 | KOG1427 | Uncharacterized conserved protein, contains RCC1 domain | Function unknown | *Aspergillus fumigatus* A1163 | XP_755403.1 |
| KF25_1495 | KOG2810 | Checkpoint 9-1-1 complex, RAD9 component | Energy production and conversion | *Aspergillus flavu*s NRRL3357 | XP_002377853.1 |
| KF25_1523 | KOG1924 | RhoA GTPase effector DIA/Diaphanous | Signal transduction mechanisms | *Neosartorya fischeri* NRRL 181 | XP_001260514.1 |
| KF25_1611 | KOG1536 | Biotin holocarboxylase synthetase/biotin-protein ligase | Coenzyme transport and metabolism | *Aspergillus kawachii* IFO 4308 | GAA85156.1 |
| KF25_1687 | KOG1504 | Ornithine carbamoyltransferase OTC/ARG3 | Amino acid transport and metabolism | *Neosartorya fischeri* NRRL 181 | XP_001267307.1 |
| KF25_1763 | KOG1454 | Predicted hydrolase/acyltransferase (alpha/beta hydrolase superfamily) | General function prediction only | *Aspergillus niger* ATCC 1015 | EHA28280.1 |
| KF25_1784 | KOG2002 | TPR-containing nuclear phosphoprotein that regulates K(+) uptake | Inorganic ion transport and metabolism | *Coccidioides posadasii* C735 delta SOWgp | XP_003069619.1 |
| KF25_1817 | KOG3010 | Methyltransferase | General function prediction only | *Arthroderma otae* CBS 113480 | XP_002844738.1 |
| KF25_1829 | KOG0161 | Myosin class II heavy chain | Cytoskeleton | *Aspergillus terreus* NIH2624 | XP_001218481.1 |
| KF25_1845 | KOG0950 | DNA polymerase theta/eta, DEAD-box superfamily | General function prediction only | *Aspergillus clavatus* NRRL 1 | XP_001269637.1 |
| KF25_1922 | KOG0029 | Amine oxidase | Secondary metabolites biosynthesis, transport and catabolism | *Verticillium dahliae* VdLs.17 | EGY19240.1 |
| KF25_1941 | KOG2688 | Transcription-associated recombination protein - Thp1p | Cell cycle control, cell division, chromosome partitioning | *Neosartorya fischeri* NRRL 181 | XP_001265212.1 |
| KF25_1989 | KOG1832 | HIV-1 Vpr-binding protein | Cell cycle control, cell division, chromosome partitioning | *Aspergillus niger* CBS 513.88 | XP_001393812.1 |
| KF25_2009 | KOG2378 | cAMP-regulated guanine nucleotide exchange factor | Signal transduction mechanisms | *Aspergillus kawachii* IFO 4308 | GAA88712.1 |
| KF25_2014 | KOG4679 | Uncharacterized protein PSP1 (suppressor of DNA polymerase alpha mutations in yeast) | General function prediction only | *Aspergillus oryzae* RIB40 | XP_001820016.2 |
| KF25_2077 | KOG0290 | Conserved WD40 repeat-containing protein AN11 | Function unknown | *Aspergillus clavatus* NRRL 1 | XP_001271746.1 |
| KF25_2165 | KOG1399 | Flavin-containing monooxygenase | Secondary metabolites biosynthesis, transport and catabolism | *Aspergillus clavatus* NRRL 1 | XP_001268460.1 |
| KF25_2366 | KOG4779 | Predicted membrane protein | Function unknown | *Aspergillus niger* CBS 513.88 | XP_001389269.2 |
| KF25_2416 | KOG3349 | Predicted glycosyltransferase | General function prediction only | *Aspergillus niger* CBS 513.88 | XP_001389332.2 |
| KF25_2594 | KOG1832 | HIV-1 Vpr-binding protein | Cell cycle control, cell division, chromosome partitioning | No hits |  |
| KF25_2634 | KOG1368 | Threonine aldolase | Amino acid transport and metabolism | *Aspergillus clavatus* NRRL 1 | XP_001273492.1 |
| KF25_3022 | KOG1183 | N-acetylglucosaminyltransferase complex, subunit PIG-Q/GPI1, required for phosphatidylinositol biosynthesis | Cell wall/membrane/envelope biogenesis | *Aspergillus oryzae* RIB40 | XP_001818203.2 |
| KF25_3374 | KOG1510 | RNA polymerase II holoenzyme and mediator subcomplex, subunit SURB7/SRB7 | Transcription | *Aspergillus clavatus* NRRL 1 | XP_001275609.1 |
| KF25_3440 | KOG0578 | p21-activated serine/threonine protein kinase | Signal transduction mechanisms | *Aspergillus nidulans* FGSC A4 | XP_659671.1 |
| KF25_3471 | KOG3860 | Acyltransferase required for palmitoylation of Hedgehog (Hh) family of secreted signaling proteins | Signal transduction mechanisms | *Neosartorya fischeri* NRRL 181 | XP_001260127.1 |
| KF25_3520 | KOG3780 | Thioredoxin binding protein TBP-2/VDUP1 | General function prediction only | *Aspergillus flavu*s NRRL3357 | XP_002375173.1 |
| KF25_4070 | KOG2210 | Oxysterol-binding protein | Signal transduction mechanisms | *Aspergillus clavatus* NRRL 1 | XP_001272805.1 |
| KF25_4090 | KOG1716 | Dual specificity phosphatase | Defense mechanisms | *Aspergillus kawachii* IFO 4308 | GAA85369.1 |
| KF25_4136 | KOG0269 | WD40 repeat-containing protein | Function unknown | *Aspergillus niger* CBS 513.88 | XP_001391822.2 |
| KF25_4279 | KOG2151 | Predicted transcriptional regulator | Transcription | *Aspergillus oryzae* RIB40 | XP_001821974.2 |
| KF25_4336 | KOG1235 | Predicted unusual protein kinase | General function prediction only | *Aspergillus terreus* NIH2624 | XP_001209075.1 |
| KF25_4415 | KOG0921 | Dosage compensation complex, subunit MLE | Transcription | No hits |  |
| KF25_4420 | KOG4704 | Uncharacterized conserved protein | Function unknown | *Aspergillus oryzae* RIB40 | XP_001818169.1 |
| KF25_4546 | KOG0443 | Actin regulatory proteins (gelsolin/villin family) | Cytoskeleton | *Neosartorya fischeri* NRRL 181 | XP_001264261.1 |
| KF25_4596 | KOG0379 | Kelch repeat-containing proteins | General function prediction only | *Aspergillus kawachii* IFO 4308 | GAA86713.1 |
| KF25_4641 | KOG0255 | Synaptic vesicle transporter SVOP and related transporters (major facilitator superfamily) | General function prediction only | *Aspergillus terreus* NIH2624 | XP_001215372.1 |
| KF25_4656 | KOG1087 | Cytosolic sorting protein GGA2/TOM1 | Intracellular trafficking, secretion, and vesicular transport | *Aspergillus oryzae* RIB40 | XP_001826013.2 |
| KF25_4666 | KOG0921 | Dosage compensation complex, subunit MLE | Transcription | No hits |  |
| KF25_4667 | KOG1363 | Predicted regulator of the ubiquitin pathway (contains UAS and UBX domains) | Signal transduction mechanisms | *Aspergillus clavatus* NRRL 1 | XP_001268827.1 |
| KF25_4732 | KOG4775 | Uncharacterized protein SFI1 involved in G(2)-M transition | Cell cycle control, cell division, chromosome partitioning | *Aspergillus terreus* NIH2624 | XP_001210664.1 |
| KF25_4733 | KOG1064 | RAVE (regulator of V-ATPase assembly) complex subunit RAV1/DMX protein, WD repeat superfamily | General function prediction only | *Aspergillus oryzae* RIB40 | BAE60674.1 |
| KF25_4985 | KOG1144 | Translation initiation factor 5B (eIF-5B) | Translation, ribosomal structure and biogenesis | *Neosartorya fischeri* NRRL 181 | XP_001264767.1 |
| KF25_5021 | KOG0953 | Mitochondrial RNA helicase SUV3, DEAD-box superfamily | RNA processing and modification | *Neosartorya fischeri* NRRL 181 | XP_001259608.1 |
| KF25_5041 | KOG0475 | Cl- channel CLC-3 and related proteins (CLC superfamily) | Inorganic ion transport and metabolism | *Aspergillus clavatus* NRRL 1 | XP_001274353.1 |
| KF25_5093 | KOG0496 | Beta-galactosidase | Carbohydrate transport and metabolism | *Neosartorya fischeri* NRRL 181 | XP_001258644.1 |
| KF25_5146 | KOG2831 | ATP phosphoribosyltransferase | Amino acid transport and metabolism | *Aspergillus oryzae* RIB40 | XP_001817248.2 |
| KF25_5154 | KOG1005 | Telomerase catalytic subunit/reverse transcriptase TERT | Replication, recombination and repair | *Aspergillus fumigatus* A1163 | XP_749051.2 |
| KF25_5163 | KOG2426 | Dihydroxyacetone kinase/glycerone kinase | Carbohydrate transport and metabolism | *Neosartorya fischeri* NRRL 181 | XP_001259429.1 |
| KF25_5320 | KOG1716 | Dual specificity phosphatase | Defense mechanisms | *Aspergillus kawachii* IFO 4308 | GAA83325.1 |
| KF25_5423 | KOG3042 | Panthothenate synthetase | Coenzyme transport and metabolism | *Aspergillus oryzae* RIB40 | BAE60150.1 |
| KF25_5480 | KOG1369 | Hexokinase | Carbohydrate transport and metabolism | *Aspergillus clavatus* NRRL 1 | XP_001273243.1 |
| KF25_5587 | KOG2822 | Sphingoid base-phosphate phosphatase | Lipid transport and metabolism | *Aspergillus kawachii* IFO 4308 | GAA90686.1 |
| KF25_5623 | KOG1840 | Kinesin light chain | Cytoskeleton | *Arthroderma gypseum* CBS 118893 | XP_003174889.1 |
| KF25_5657 | KOG2366 | Alpha-D-galactosidase (melibiase) | Carbohydrate transport and metabolism | *Aspergillus fumigatus* A1163 | XP_753271.1 |
| KF25_5754 | KOG1924 | RhoA GTPase effector DIA/Diaphanous | Signal transduction mechanisms | No hits |  |
| KF25_5813 | KOG1803 | DNA helicase | Replication, recombination and repair | *Aspergillus niger* | CAK96441.1 |
| KF25_6094 | KOG0055 | Multidrug/pheromone exporter, ABC superfamily | Secondary metabolites biosynthesis, transport and catabolism | *Chitinophaga pinensis* DSM 2588 | YP_003125917.1 |
| KF25_6095 | KOG0054 | Multidrug resistance-associated protein/mitoxantrone resistance protein, ABC superfamily | Secondary metabolites biosynthesis, transport and catabolism | *Bradyrhizobium japonicum* USDA 6 | YP_005608020.1 |
| KF25_6192 | KOG3720 | Lysosomal & prostatic acid phosphatases | Lipid transport and metabolism | *Aspergillus oryzae* RIB40 | XP_001818042.2 |
| KF25_6369 | KOG1238 | Glucose dehydrogenase/choline dehydrogenase/mandelonitrile lyase (GMC oxidoreductase family) | General function prediction only | *Penicillum digitatum* Pd1 | EKV11133 |
| KF25_6432 | KOG2571 | Chitin synthase/hyaluronan synthase (glycosyltransferases) | Cell wall/membrane/envelope biogenesis | *Aspergillus niger* CBS 513.88 | XP_001395099.2 |
| KF25_6502 | KOG2511 | Nicotinic acid phosphoribosyltransferase | Coenzyme transport and metabolism | *Neosartorya fischeri* NRRL 181 | XP_001262825.1 |
| KF25_6695 | KOG1502 | Flavonol reductase/cinnamoyl-CoA reductase | Defense mechanisms | *Aspergillus niger* | CAK46198.1 |
| KF25_6837 | KOG2154 | Predicted nucleolar protein involved in ribosome biogenesis | Translation, ribosomal structure and biogenesis | *Aspergillus clavatus* NRRL 1 | XP_001276649.1 |
| KF25_6867 | KOG2262 | Sexual differentiation process protein ISP4 | Signal transduction mechanisms | *Aspergillus kawachii* IFO 4308 | GAA85903.1 |
| KF25_6900 | KOG2071 | mRNA cleavage and polyadenylation factor I/II complex, subunit Pcf11 | RNA processing and modification | *Aspergillus niger* | CAK45137.1 |
| KF25_6902 | KOG0161 | Myosin class II heavy chain | Cytoskeleton | *Aspergillus oryzae* RIB40 | XP_001727931.2 |
| KF25_7122 | KOG4139 | Protein kinase essential for the initiation of DNA replication | Replication, recombination and repair | *Neosartorya fischeri* NRRL 181 | XP_001258036.1 |
| KF25_7265 | KOG1270 | Methyltransferases | Coenzyme transport and metabolism | *Aspergillus oryzae* RIB40 | XP_001816548.2 |
| KF25_7698 | KOG1616 | Protein involved in Snf1 protein kinase complex assembly | Carbohydrate transport and metabolism | *Neosartorya fischeri* NRRL 181 | XP_001257655.1 |
| KF25_7734 | KOG0162 | Myosin class I heavy chain | Cytoskeleton | No hits |  |
| KF25_7750 | KOG3968 | Atrazine chlorohydrolase/guanine deaminase | Nucleotide transport and metabolism | *Aspergillus niger* CBS 513.88 | XP_001397402.1 |
| KF25_7790 | KOG1176 | Acyl-CoA synthetase | Lipid transport and metabolism | *Aspergillus flavu*s NRRL3357 | XP_002378810.1 |
| KF25_7821 | KOG1817 | Ribonuclease | RNA processing and modification | *Aspergillus kawachii* IFO 4308 | GAA88143.1 |
| KF25_7834 | KOG0084 | GTPase Rab1/YPT1, small G protein superfamily, and related GTP-binding proteins | Signal transduction mechanisms | *Aspergillus niger* CBS 513.88 | XP_001393994.1 |
| KF25_7835 | KOG0769 | Predicted mitochondrial carrier protein | Energy production and conversion | *Aspergillus niger* CBS 513.88 | XP_001393993.1 |
| KF25_7843 | KOG3385 | V-SNARE | Intracellular trafficking, secretion, and vesicular transport | *Aspergillus terreus* NIH2624 | XP_001209478.1 |
| KF25_7911 | KOG0809 | SNARE protein TLG2/Syntaxin 16 | Intracellular trafficking, secretion, and vesicular transport | *Aspergillus niger* CBS 513.88 | XP_001402063.2 |
| KF25_8097 | KOG1663 | O-methyltransferase | Secondary metabolites biosynthesis, transport and catabolism | *Aspergillus clavatus* NRRL 1 | XP_001274019.1 |
| KF25_8189 | KOG1807 | Helicases | Replication, recombination and repair | *Thielavia terrestris* NRRL 8126 | XP_003654692.1 |
| KF25_8230 | KOG0232 | Vacuolar H+-ATPase V0 sector, subunits c/c' | Energy production and conversion | *Aspergillus clavatus* NRRL 1 | XP_001274195.1 |
| KF25_8362 | KOG2058 | Ypt/Rab GTPase activating protein | Intracellular trafficking, secretion, and vesicular transport | *Aspergillus clavatus* NRRL 1 | XP_001271281.1 |
| KF25_8447 | KOG2614 | Kynurenine 3-monooxygenase and related flavoprotein monooxygenases | Energy production and conversion | *Fusarium oxysporum* Fo5176 | EGU88059.1 |
| KF25_8575 | KOG0460 | Mitochondrial translation elongation factor Tu | Translation, ribosomal structure and biogenesis | *Azoarcus sp.* KH32C | BAL26285.1 |
| KF25_8695 | KOG4122 | Mitochondrial/chloroplast ribosomal protein L36 | Translation, ribosomal structure and biogenesis | *Aspergillus niger* CBS 513.88 | XP_003188658.1 |
| KF25_8791 | KOG1806 | DEAD box containing helicases | Replication, recombination and repair | *Aspergillus oryzae* RIB40 | XP_001826562.2 |
| KF25_8943 | KOG4701 | Chitinase | Cell wall/membrane/envelope biogenesis | *Aspergillus kawachii* IFO 4308 | GAA88232.1 |
| KF25_8973 | KOG1035 | eIF-2alpha kinase GCN2 | Translation, ribosomal structure and biogenesis | *Trichoderma virens* Gv29-8 | EHK25102.1 |
| KF25_8992 | KOG0293 | WD40 repeat-containing protein | Function unknown | *Aspergillus oryzae* RIB40 | XP_001822546.2 |
| KF25_9015 | KOG0183 | 20S proteasome, regulatory subunit alpha type PSMA7/PRE6 | Posttranslational modification, protein turnover, chaperones | *Aspergillus terreus* NIH2624 | XP_001217792.1 |
| KF25_9058 | KOG0725 | Reductases with broad range of substrate specificities | General function prediction only | *Trichoderma virens* Gv29-8 | EHK23150.1 |
| KF25_9173 | KOG0224 | Aquaporin (major intrinsic protein family) | Carbohydrate transport and metabolism | *Aspergillus oryzae* RIB40 | XP_001825721.2 |
| KF25_9213 | KOG0475 | Cl- channel CLC-3 and related proteins (CLC superfamily) | Inorganic ion transport and metabolism | *Aspergillus flavu*s NRRL3357 | XP_002379511.1 |
| KF25_9322 | KOG0157 | Cytochrome P450 CYP4/CYP19/CYP26 subfamilies | Secondary metabolites biosynthesis, transport and catabolism | *Grosmannia clavigera* kw1407 | EFX00361.1 |
| KF25_9323 | KOG1285 | Beta, beta-carotene 15,15 '-dioxygenase and related enzymes | Secondary metabolites biosynthesis, transport and catabolism | *Podospora anserina* S mat+ | XP_001906512.1 |
| KF25_9325 | KOG0255 | Synaptic vesicle transporter SVOP and related transporters (major facilitator superfamily) | General function prediction only | *Aspergillus niger* CBS 513.88 | XP_001398571.2 |
| KF25_9435 | KOG0254 | Predicted transporter (major facilitator superfamily) | General function prediction only | *Neosartorya fischeri* NRRL 181 | XP_001261621.1 |
| KF25_9525 | KOG1202 | Animal-type fatty acid synthase and related proteins | Lipid transport and metabolism | *Pyrenophora tritici-repentis* Pt-1C-BFP | XP_001934016.1 |
| KF25_9553 | KOG2806 | Chitinase | Carbohydrate transport and metabolism | *Aspergillus nidulans* FGSC A4 | XP_658121.1 |
| KF25_9727 | KOG1212 | Amidases | Translation, ribosomal structure and biogenesis | *Neosartorya fischeri* NRRL 181 | XP_001258157.1 |
| KF25_9742 | KOG2806 | Chitinase | Carbohydrate transport and metabolism | *Aspergillus niger* ATCC 1015 | EHA24975.1 |
| KF25_9764 | KOG0156 | Cytochrome P450 CYP2 subfamily | Secondary metabolites biosynthesis, transport and catabolism | *Aspergillus oryzae* RIB40 | XP_001824588.1 |
| KF25_9774 | KOG1985 | Vesicle coat complex COPII, subunit SEC24/subunit SFB2 | Intracellular trafficking, secretion, and vesicular transport | *Aspergillus fumigatus* A1163 | EDP49856.1 |
| KF25_9775 | KOG1163 | Casein kinase (serine/threonine/tyrosine protein kinase) | Signal transduction mechanisms | *Aspergillus fumigatus* A1163 | XP_747429.1 |
| KF25_9779 | KOG0663 | Protein kinase PITSLRE and related kinases | General function prediction only | *Ajellomyces capsulatus* H88 | EGC41939.1 |
| KF25_9783 | KOG2029 | Uncharacterized conserved protein | Function unknown | *Arthroderma otae* CBS 113480 | XP_002849133.1 |
| KF25_9798 | KOG0510 | Ankyrin repeat protein | General function prediction only | *Talaromyces stipitatus* ATCC 10500 | XP_002488826.1 |





**Supplemental Figure 4. Classifications of the origin of the most similar genes in GenBank of the 355 KF-25 specific genes.**





**Supplemental Figure 5.** **Neighor-Joining phylogenetic tree of *P. chrysogenum* KF-25 and other species of the genus of penicillium based on the *benA* gene.** The phylogenetic tree was constructed by using the Mega 5.05 [91] with neighbor-joining method and bootstrap analysis (1,000 replicates) of a Muscle alignment of the *benA* genes [97] nucleotide sequence. All genes with the GenBank numbers indicated in the brackets and the strains *P. chrysogenum* KF-25 was shown in red.

**Supplemental Table 7. Detail information of the predicted secondary metabolism gene clusters.** The gene clusters were predicted by using antiSMASH and all the functions of each proteins were indicated. The type of the putative secondary metabolism product and the corresponding ORFs in *P. chrysogenum* Wisconsin 54-1255 were indicated. The letters above the number were corresponded with the structure in Supplemental Figure 5.

| Number | Type | Compound produced with similarity gene cluster | ORFs | Putative protein function | Corresponding ORFs in *Pencillium chrysogenum* Wisconsisn 54-1255 |
| --- | --- | --- | --- | --- | --- |
| 1 | t1PKS | stigmatellin | KF25_0124 | polyketide synthase, similarity with protein in *Neosartorya fischeri* NRRL 181(gb\|EAW16387.1, 55% identify) | Pc21g00960 |
|  |  |  | KF25_0125 | hypothetical protein | Pc21g00950 |
|  |  |  | KF25_0126 | aminoadipate-semialdehyde dehydrogenase, weak similarity with protein in *Talaromyces stipitatus* ATCC 10500 (gb\|EED14394.1, 28% identify) | Pc21g00940 |
|  |  |  | KF25_0127 | protein kinase, strong similarity with protein in *Aspergillus clavatus* NRRL 1 (gb\|EAW11545.1, 83% identify) | Pc21g00930 |
| 2 | other |  | KF25_0364 | MFS transporter, strong similarity with protein in *Penicillium digitatum* ([gb\|ADN97076.1](http://www.ncbi.nlm.nih.gov/protein/307950782?report=genbank&log$=protalign&blast_rank=2&RID=BAYSHYFP01R), 87% identify) | Pc20g02530 |
|  |  |  | KF25_0365 | transmembrane protein, similarity with protein in *Paracoccidioides brasiliensis* Pb18 (gb\|EEH47240.1, 58% identify) | Pc20g02560 |
|  |  |  | KF25_0368 | nonribosomal peptide synthase, smilarity with protein in *Neosartorya fischeri* NRRL 181 (gb\|EAW17166.1, 47% identify) | Pc20g02590 |
|  |  |  | KF25_0369 | cytochrome c oxidase, cbb3-type, subunit I, similarity with protein in *Campylobacter concisus* 13826 (gb\|ABW74731.1, 23% identify) | Pc20g02600 |
|  |  |  | KF25_372 | vacuolar carboxypeptidase, similarity with protein in *Aspergillus clavatus*  NRRL 1 (gb\|EAW09297.1, 60% identify) | Pc20g02630 |
|  |  |  | KF25_373 | glutaminase, similarity with protein in *Aspergillus fumigatus* Af293 (gb\|EAL92864.1, 69% identify) | Pc20g02640 |
| 3 | NRPS |  | KF25_913 | L-ornithine N5-oxygenase, similarity with protein in *Neosartorya fischeri* NRRL 181 (gb\|EAW18390.1, 72% identify) | Pc13g05260 |
|  |  |  | KF25_914 | nonribosomal siderophore peptide synthase, similarity with protein in *Aspergillus clavatus* NRRL 1 (gb\|EAW06285.1, 54% identify) | Pc13g05250 |
|  |  |  | KF25_915 | protein kinase, similarity with protein in *Neosartorya fischeri* NRRL 181 (gb\|EAW18391.1, 69% identify) | Pc13g05240 |
| 4 | t1PKS | chalcomycin | KF25_1802 | MFS aflatoxin efflux pump, similarity with protein in *Aspergillus clavatus* NRRL 1 (gb\|EAW12031.1, 73% identify) | Pc16g10850 |
|  |  |  | KF25_1803 | monoxygenase, similarity with protein in *Talaromyces stipitatus* ATCC 10500 (gb\|EED18967.1, 65% identify) | Pc16g10840 |
|  |  |  | KF25_1806 | NADP(+)-dependent dehydrogenase, similarity with protein in *Neosartorya fischeri* NRRL 181 (gb\|EAW24669.1, 68% identify) | Pc16g10810 |
|  |  |  | KF25_1808 | NAD-dependent epimerase/dehydratase, weak similarity with protein in *Macrophomina phaseolina* MS6 (gb\|EKG11407.1, 32% identify) | Pc16g10790 |
|  |  |  | KF25_1811 | scytalone dehydratase, similarity with protein in *Arthroderma otae* CBS 113480 (gb\|EEQ33876.1, 78% identify) | Pc16g10760 |
|  |  |  | KF25_1812 | polyketide synthase, similarity with protein in *Neosartorya fischeri* NRRL 181 (gb\|EAW24697.1, 63% identify) |  |
|  |  |  | KF25_1813 | Metallo-beta-lactamase domain protein, similarity with protein in *Talaromyces stipitatus* ATCC 10500 (gb\|EED18935.1, 70% identify) | Pc16g09730(49% identify) |
|  |  |  | KF25_1814 | 17-beta-hydroxysteroid dehydrogenase, similarity with protein in *Arthroderma otae* CBS 113480 (gb\|EEQ33886.1, 89% identify) |  |
|  |  |  | KF25_1815 | NAD-dependent epimerase, similarity with protein in *Grosmannia clavigera* kw1407 (gb\|EFX02673.1, 40% identify) |  |
|  |  |  | KF25_1817 | Methyltransferase, weak similarity with protein in *Cordyceps militaris* CM01 (gb\|EGX94264.1, 27% identify) | Pc13g06310(23% identify) |
|  |  |  | KF25_1818 | aflatoxin biosynthesis regulatory protein, similarity with protein in *Arthroderma otae* CBS 113480 (gb\|EEQ33882.1, 55% identify) | Pc16g09740 (40% identify) |
|  |  |  | KF25_1819 | sterol glucosyltransferase, similarity with protein in *Aspergillus niger* CBS 513.88 (XP_001394072.2, 52% identify) | Pc12g02420 (51% identify) |
| 5 | other |  | KF25_1922 | FAD dependent oxidoreductase, similarity with protein in *Metarhizium anisopliae* ARSEF 23 (gb\|EFY98028.1, 65% identify) |  |
|  |  |  | KF25_1923 | 6-phosphogluconolactonase, similarity with protein in *Aspergillus kawachii* IFO 4308 (dbj\|GAA83587.1, 60% identify) | Pc22g09680 (50% identify) |
|  |  |  | KF25_1927 | FAD binding domain protein, similarity with protein in *Neosartorya fischeri* NRRL 181 (gb\|EAW19902.1, 53% identify) | Pc16g09430 |
|  |  |  | KF25_1928 | non-ribosomal peptide synthetase, similarity with protein in *Aspergillus niger* ATCC 1015 (gb\|EHA20666.1, 53% identify) | Pc16g09420 |
|  |  |  | KF25_1929 | phosphatase regulatory subunit, similarity with protein in *Aspergillus niger* CBS 513.88 (emb\|CAK36910.1, 79% identify) | Pc16g09410 |
|  |  |  | KF25_1930 | general stress response phosphoprotein phosphatase, similarity with protein in *Aspergillus fumigatus* Af293 (gb\|EAL88190.1, 52% similarity) | Pc16g09400 |
|  |  |  | KF25_1931 | peroxisomal ABC transporter, similarity with protein in *Aspergillus oryzae* RIB40 (dbj\|BAE57989.1, 87% identify) | Pc16g09390 |
| 6 | other |  | KF25_2638 | AAA family ATPase, similarity with protein in *Neosartorya fischeri* NRRL 181 (gb\|EAW19721.1, 57% identify) | Pc22g06290 |
|  |  |  | KF25_2639 | L-aminoadipate-semialdehyde dehydrogenase large subunit, similarity with protein in *Penicillium chrysogenum* Wisconsin 54-1255 (emb\|CAP97919.1, 99% identify) | Pc22g06310 |
|  |  |  | KF25_2640 | Nep1 ribosome biogenesis protein, similarity with protein in *Candidatus Nitrosopumilus* salaria BD31 (gb\|EIJ65317.1, 27% identify) | Pc22g06320 |
|  |  |  | KF25_2641 | xanthine dehydrogenase, similarity with protein in *Aspergillus clavatus* NRRL 1(gb\|EAW10559.1, 84% identify) | Pc22g06330 |
|  |  |  | KF25_2644 | kynurenine aminotransferase, similarity with protein in *Aspergillus kawachii* IFO 4308 (dbj\|GAA82949.1, 91% identify) | Pc22g06360 |
|  |  |  | KF25_2645 | AAA family ATPase, similarity with protein in *Aspergillus fumigatus* Af293 (gb\|EAL89673.1, 81% identify) | Pc22g06370 |
|  |  |  | KF25_2648 | ADAM family of metalloprotease, similarity with protein in *Aspergillus flavus* NRRL3357 (gb\|EED55442.1, 59% identify) | Pc22g06400 |
| 7 | other |  | KF25_2915 | hexose carrier protein, similarity with protein in *Aspergillus oryzae* RIB40 (gb\|EIT76663.1, 68% identify) | Pc18g00390 |
|  |  |  | KF25_2916 | hybrid NRPS/PKS enzyme, similarity with protein in *Aspergillus fumigatus* A1163 (gb\|EDP51754.1, 75% identify) | Pc18g00380 |
|  |  |  | KF25_2918 | transglycosylase/penicillin-binding protein, similarity with protein in *Streptococcus intermedius* SK54 (gb\|EID83714.1, 30 identify) | Pc18g00350 |
| 8 | other |  | KF25_3504 | acyl-CoA synthetases /AMP-acid ligases, similaritye with protein in *Arthroderma otae* CBS 113480 (gb\|EEQ29767.1, 50% identify) | Pc12g09980 |
|  |  |  | KF25_3505 | tetratricopeptide repeat domain-containing protein, similarity with protein in *Neosartorya fischeri* NRRL 181 (gb\|EAW20855.1, 29% identify) | Pc12g09960 |
|  |  |  | KF25_3506 | alcohol oxidase, strong similarity with protein in *Penicillium chrysogenum* Wisconsin 54-1255 (gb\|AAL56054.1\|AF329938_1, 100% identify) | Pc12g09950 |
|  |  |  | KF25_3507 | amidotransferase, similarity with protein in *Agrobacterium tumefaciens* 5A (gb\|EHJ95523.1, 33% identify) | Pc12g09930 |
|  |  |  | KF25_3510 | ABC transporter, similarity with protein in *Aspergillus clavatus* NRRL 1 (gb\|EAW06570.1, 73% identify) | Pc12g09900 |
| 9 | t1PKS |  | KF25_3640 | methionine aminopeptidase, similarity with protein in *Aspergillus oryzae* RIB40 (dbj\|BAE62843.1, 74% identify) | Pc14g00010 |
|  |  |  | KF25_3641 | putative Zn(II)2Cys6 transcription factor, similarity with protein in *Aspergillus nidulans* FGSC A4 (tpe\|CBF87498.1, 55% identify) | Pc14g00020 |
|  |  |  | KF25_3642 | taurine catabolism dioxygenase, similarity with protein in *Magnaporthe oryzae* 70-15 (gb\|EHA51734.1, 65% identify) | Pc14g00030 |
|  |  |  | KF25_3643 | MFS transporter, similarity with protein in *Aspergillus oryzae* RIB40 (dbj\|BAE65569.1, 74% identify) | Pc14g00060 |
|  |  |  | KF25_3644 | polyketide synthase/peptide synthetase/ Beta-ketoacyl synthase, similarity with protein in *Magnaporthe oryzae* 70-15 (gb\|EHA55860.1, 44% identify) | Pc14g00080 |
|  |  |  | KF25_3645 | Alcohol dehydrogenase/zinc-binding dehydrogenase family oxidoreductase, similarity with protein in *Aspergillus clavatus* NRRL 1 (gb\|EAW09121.1, 48% identify) | Pc14g00090 |
|  |  |  | KF25_3648 | cytochrome P450, similarity with protein in *Aspergillus oryzae* RIB40 (GENE ID: 5990501 AOR_1_132174, 54% identify) | Pc14g00120 |
|  |  |  | KF25_3649 | acyl-CoA dehydrogenase, similarity with protein in *Aspergillus flavus* NRRL3357 (gb\|EED53053.1, 79% identify) | Pc14g00140 |
|  |  |  | KF25_3650 | arginine permease, similarity with protein in *Aspergillus kawachii* IFO 4308 (dbj\|GAA92580.1, 53% identify) | Pc14g00150 |
|  |  |  | KF25_3651 | MFS monocarboxylate transporter, similarity with protein in *Aspergillus clavatus* NRRL 1 (gb\|EAW12319.1, 57% identify) | Pc14g00160 |
|  |  |  | KF25_3652 | phosphatidylglycerol specific phospholipase, similarity with protein in *Aspergillus clavatus* NRRL 1 (gb\|EAW12320.1, 69% identify) | Pc14g00170 |
|  |  |  | KF25_3653 | dihydroxyacetone synthase, similarity with protein in *Aspergillus terreus* NIH2624 (gb\|EAU30218.1, 82% identify) | Pc14g00180 |
|  |  |  | KF25_3654 | alcohol dehydrogenase, similarity with protein in *Penicillium marneffei* ATCC 18224 (gb\|EEA23486.1, 76% identify) | Pc14g00190 |
| 10 | other |  | KF25_3786 | enolase, similarity with protein in Penicillium chrysogenum (emb\|CAP74315.1, 99% identify) | Pc14g01740 |
|  |  |  | KF25_3787 | mannosyl-oligosaccharide alpha-1,2-mannosidase, similarity with protein in *Aspergillus fumigatus* Af293 (gb\|EAL88534.1, 62% identify) | Pc14g01760 |
|  |  |  | KF25_3788 | peptidase, similarity with protein in *Aspergillus flavus* NRRL3357 (gb\|EED56471.1, 64% identify) | Pc14g01770 |
|  |  |  | KF25_3789 | NRPS-like enzyme, similarity with protein in *Aspergillus oryzae* RIB40 (GENE ID: 5999494 AOR_1_580024, 53% identify) | Pc14g01790 |
|  |  |  | KF25_3790 | beta-galactosidase, similarity with protein in *Sporisorium reilianum* SRZ2 (emb\|CBQ72762.1, 48% identify) | Pc14g01800 |
| 11 | other |  | KF25_4465 | MFS maltose transporter, similarity with protein in *Talaromyces stipitatus* ATCC 10500 (gb\|EED16366.1, 89% identify) | Pc06g01480 |
|  |  |  | KF25_4466 | MFS amine transporter, similarity with protein in Ajellomyces capsulatus H143 (gb\|EER42931.1, 34 identify) | Pc06g01490 |
|  |  |  | KF25_4467 | putative methyltransferase, weak similarity with protein in *Stigmatella aurantiaca* DW4/3-1 (gb\|EAU68891.1, 29% identify) | Pc06g01500 |
|  |  |  | KF25_4468 | NAD dependent epimerase/dehydratase, similarity with protein in *Aspergillus oryzae* RIB40 (GENE ID: 5987842 AOR_1_564194, 35% identify) | Pc06g01510 |
|  |  |  | KF25_4469 | benzoate 4-monooxygenase cytochrome P450, similarity with protein in *Trichophyton equinum* CBS 127.97 (gb\|EGE02686.1, 63% identify) | Pc06g01520 |
|  |  |  | KF25_4470 | NmrA-like family protein, similarity with protein in *Aspergillus oryzae* RIB40 (ref\|XP_001824589.2, 66% identify) | Pc06g01530 |
|  |  |  | KF25_4471 | non-ribosomal peptide synthetase, similarity with protein in *Aspergillus oryzae* 3.042 (gb\|EIT72370.1,65% identify) | Pc06g01540 |
|  |  |  | KF25_4472 | Beta-lactamase-related protein, similarity with protein in *Macrophomina phaseolina* MS6 (gb\|EKG15302.1, 56% identify) |  |
|  |  |  | KF25_4473 | thiopurine S-methyltransferase family protein, similarity with protein in *Aspergillus oryzae* RIB40 (ref\|XP_003190605.1, 63% identify) | Pc06g01550 |
| 12**^a^** | NRPS |  | KF25_4492 | geranylgeranyl pyrophosphate synthase, similarity with protein in *Neosartorya fischeri* NRRL 181 (gb\|EAW24472.1, 43% identify) | Pc20g10860 (40% identify) |
|  |  |  | KF25_4493 | nonribosomal peptide synthase/HC-toxin synthase, similarity with protein in *Aspergillus oryzae* RIB40 (dbj\|BAE64605.1, 64% identify) | Pc21g10790 |
|  |  |  | KF25_4494 | aristolochene synthase, similarity with protein in *Colletotrichum higginsianum* (emb\|CCF37943.1, 39% identify) | Pc21g10770/ Pc21g10780 |
|  |  |  | KF25_4495 | ATP binding protein, similarity with protein in *Macrophomina phaseolina* MS6 (gb\|EKG16571.1, 40% identify) | Pc21g10750 |
| 13**^b^** | t1PKS |  | KF25_5457 | MFS transporter, similarity with protein in *Aspergillus clavatus* NRRL 1 (gb\|EAW06503.1, 76% identify) | Pc21g05100 |
|  |  |  | KF25_5459 | polyketide synthase, similarity with protein in *Coccidioides posadasii* C735 delta SOWgp (gb\|EER25595.1, 49% identify) | Pc21g05080 |
|  |  |  | KF25_5460 | polyketide synthase, similarity with protein in *Trichoderma reesei* QM6a (gb\|EGR52182.1, 65% identify) | Pc21g05070 |
|  |  |  | KF25_5461 | monooxygenase FAD-binding protein, similarity with protein in *Macrophomina phaseolina* MS6 (gb\|EKG11421.1, 47% identify) | Pc21g05060 |
|  |  |  | KF25_5462 | citrinin biosynthesis transcriptional activator CtnR, similarity with protein in *Monascus purpureus* (dbj\|BAE95337.1, 34% identify) | Pc21g05050 |
| 14 | t1PKS | epothilone | KF25_5476 | Uridylate kinase, similarity with protein in *Penicillium digitatum* Pd1 (gb\|EKV05524.1, 97% identify) | Pc21g04870 |
|  |  |  | KF25_5477 | PII uridylyl-transferase, similarity with protein in *Candidatus Accumulibacter phosphatis* clade IIA str. UW-1 (gb\|ACV36158.1, 35% identify) | Pc21g04860 |
|  |  |  | KF25_5478 | DUF341 family oxidoreductase, similarity with protein in *Penicillium marneffei* ATCC 18224 (gb\|EEA20044.1, 61% identify) | Pc21g04850 |
|  |  |  | KF25_5479 | polyketide synthase, similarity with protein in *Penicillium marneffei* ATCC 18224 (gb\|EEA20045.1, 63% identify) | Pc21g04840 |
|  |  |  | KF25_5480 | Hexokinase-1, similarity with protein in *Penicillium digitatum* Pd1 (gb\|EKV05518.1, 90% identify) |  |
|  |  |  | KF25_5481 | transport-related membrane protein, similarity with protein in Bacteroides fragilis 638R (emb\|CBW24284.1, 31% identify) | Pc21g04810 |
|  |  |  | KF25_5482 | arylsulfotransferase, similarity with protein in *Aspergillus kawachii* IFO 4308 (dbj\|GAA84268.1, 59% identify) | Pc21g04800 |
|  |  |  | KF25_5483 | phosphotransferase enzyme family protein, similarity with protein in *Aspergillus oryzae* RIB40 (ref\|XP_003190096.1, 58% identify) | Pc21g04790 |
|  |  |  | KF25_5484 | NADH-cytochrome B5 reductase, similarity with protein in *Aspergillus oryzae* RIB40 (dbj\|BAE64697.1, 69% identify) | Pc21g04770 |
|  |  |  | KF25_5485 | cysteine dioxygenase, similarity with protein in *Aspergillus terreus* NIH2624 (gb\|EAU33889.1, 73% identify) | Pc21g04760 |
| 15**^c^** | NRPS |  | KF25_5701 | quinate permease, similarity with protein in *Pyrenophora tritici-repentis* Pt-1C-BFP (gb\|EDU51417.1, 63% identify) | Pc21g01730 |
|  |  |  | KF25_5702 | amino acid transporter, similarity with protein in *Macrophomina phaseolina* MS6 (gb\|EKG21088.1, 54% identify) | Pc21g01720 |
|  |  |  | KF25_5703 | nonribosomal peptide synthase, similarity with protein in *Aspergillus clavatus* NRRL 1 (gb\|EAW12158.1, 65% identify) | Pc21g01710 |
|  |  |  | KF25_5704 | enoyl reductase, similarity with protein in *Aspergillus clavatus*  NRRL 1 (gb\|EAW12157.1, 66% identify) | Pc21g01700 |
|  |  |  | KF25_5705 | MFS monocarboxylate transporter, similarity with protein in *Aspergillus clavatus* NRRL 1 (gb\|EAW12156.1, 75% identify) | Pc21g01690 |
|  |  |  | KF25_5708 | thioesterase, similarity with protein in *Penicillium digitatum* Pd1 (gb\|EKV06284.1, 92% identify) | Pc21g01650 |
| 16 | other |  | KF25_6009 | aminotransferase, similarity with protein in *Aspergillus flavus* NRRL3357 (gb\|EED50269.1, 57% identify) | Pc13g12580 |
|  |  |  | KF25_6010 | non-ribosomal peptide synthetase, similarity with protein in *Aspergillus flavus* NRRL3357 (gb\|EED50270.1, 59% identify) | Pc13g12570 |
|  |  |  | KF25_6012 | GTP-binding protein, similarity with protein in Penicillium digitatum PHI26 (gb\|EKV06266.1, 97% identify) | Pc13g12540 |
|  |  |  | KF25_6014 | SET domain protein/ histone-lysine N-methyltransferase setd3, similarity with protein in *Aspergillus clavatus* NRRL 1 (gb\|EAW07477.1, 56% identify) | Pc13g12520 |
| 17 | siderophore |  | KF25_6114 | short-chain dehydrogenase, similarity with protein in *Neosartorya fischeri* NRRL 181 (gb\|EAW19764.1, 71% identify) | Pc18g00360 |
|  |  |  | KF25_6115 | amidase, similarity with protein in *Cordyceps militaris* CM01 (gb\|EGX95643.1, 36% identify) | Pc13g11520 |
|  |  |  | KF25_6117 | alpha/beta hydrolase fold domain containing protein, similarity with protein in *Marssonina brunnea* f. sp. 'multigermtubi' MB_m1(gb\|EKD12922.1, 52% identify) | Pc13g11500 |
|  |  |  | KF25_6118 | integral membrane protein, similarity with protein in *Colletotrichum higginsianum* (mb\|CCF33567.1, 52% identify) | Pc13g11490 |
|  |  |  | KF25_6120 | nitrate transporter, similarity with protein in *Neosartorya fischeri* NRRL 181 (gb\|EAW22582.1, 74% identify) | Pc13g11470 |
|  |  |  | KF25_6122 | NAD [80] H-nitrite reductase, similarity with protein in *Penicillium digitatum* PHI26 (gb\|EKV18111.1, 86% identify) | Pc13g11420 |
|  |  |  | KF25_6123 | nitrate reductase, similarity with protein in *Penicillium chrysogenum* Wisconsin 54-1255 (emb\|CAP92210.1, 99% identify) | Pc13g11410 |
| 18 | NRPS |  | KF25_6152 | Homogentisate 1,2-dioxygenase, similarity with protein in *Penicillium digitatum* Pd1 (gb\|EKV15835.1, 88% identify) | Pc21g22490/ Pc21g22500 |
|  |  |  | KF25_6153 | delta-aminolevulinic acid dehydratase, similarity with protein in *Arthroderma gypseum* CBS 118893 (gb\|EFR02786.1, 79% identify) | Pc16g01700 (61% identify) |
|  |  |  | KF25_6154 | aminotransferase class-2 pyridoxal-phosphate binding site, similarity with protein in *Macrophomina phaseolina* MS6 (gb\|EKG16198.1, 73% identify) | Pc22g13500 (72% identify) |
|  |  |  | KF25_6155 | nonribosomal peptide synthetase, similarity with protein in *Arthroderma gypseum* CBS 118893 (gb\|EFR02791.1, 53% identify) | Pc21g22530 |
|  |  |  | KF25_6157 | porphobilinogen deaminase, similarity with protein in *Neosartorya fischeri* NRRL 181 (gb\|EAW18425.1, 89% identify) | Pc21g06870 (39% identify) |
|  |  |  | KF25_6158 | cytochrome P450 phenylacetate 2-hydroxylase, similarity with protein in *Neosartorya fischeri* NRRL 181 (gb\|EAW24462.1, 42% identify) | Pc21g22560 |
| 19 | other |  | KF25_6161 | folylpolyglutamate synthase, similarity with protein in *Ajellomyces dermatitidis* ER-3 (gb\|EEQ92185.1, 48% identify) |  |
|  |  |  | KF25_6164 | NRPS-like enzyme, similarity with protein in *Aspergillus kawachii* IFO 4308 (dbj\|GAA89741.1, 69% identify) | Pc21g22650 |
|  |  |  | KF25_6165 | Zn(II)2Cys6 transcription factor, similarity with protein in *Aspergillus niger* CBS 513.88 (ref\|XP_001389879.2, 57% identify) | Pc21g22670 |
|  |  |  | KF25_6166 | MFS transporter, similarity with protein in *Aspergillus oryzae* RIB40 (ref\|XP_001821703.2, 76% identify) | Pc21g22680 |
|  |  |  | KF25_6167 | amino acid transporter, similarity with protein in *Aspergillus oryzae* 3.042 (gb\|EIT75763.1, 70% identify) | Pc21g22710 |
| 20 | t1PKS |  | KF25_6328 | cytochrome P450, similarity with protein in *Aspergillus niger* CBS 513.88 (emb\|CAK49102.1, 81% identify) | Pc16g00400 |
|  |  |  | KF25_6329 | amidohydrolase, similarity with protein in *Aspergillus niger* ATCC 1015 (gb\|EHA22194.1, 80% identify) | Pc16g00390 |
|  |  |  | KF25_6330 | UbiA-like prenyltransferase, similarity with protein in *Penicillium digitatum* PHI26 (gb\|EKV11530.1, 89% identify) | Pc16g00380 |
|  |  |  | KF25_6331 | polyketide synthase, similarity with protein in *Penicillium digitatum* PHI26 (gb\|EKV11531.1, 86% identify) | Pc16g00370 |
|  |  |  | KF25_6332 | cytochrome P450, similarity with protein in *Aspergillus niger* CBS 513.88 (ref\|XP_001402409.2, 90% identify) | Pc16g00360 |
|  |  |  | KF25_6333 | toxin biosynthesis protein, similarity with protein in *Coprinopsis cinerea* okayama7#130 (gb\|EFI28251.1, 28% identify) | Pc16g00350 |
|  |  |  | KF25_6334 | short-chain dehydrogenase/reductase family protein, similarity with protein in *Aspergillus niger* CBS 513.88 (emb\|CAK49108.1, 76% identify) | Pc16g00340 |
| 21**^d^** | NRPS |  | KF25_6505 | FAD binding domain-containing protein, similarity with protein in *Arthroderma otae* CBS 113480 (gb\|EEQ35787.1, 73% identify) | Pc21g12590 |
|  |  |  | KF25_6506 | short chain dehydrogenase/reductase family oxidoreductase, similarity with protein in *Neosartorya fischeri* NRRL 181 (gb\|EAW23748.1, 84% identify) | Pc21g12600 |
|  |  |  | KF25_6507 | GA4 desaturase, similarity with protein in *Arthroderma otae* CBS 113480 (gb\|EEQ35785.1, 75% identify) | Pc21g12610 |
|  |  |  | KF25_6508 | asparagine synthase, similarity with protein in *Arthroderma otae* CBS 113480 ([gb\|EEQ35784.1](http://www.ncbi.nlm.nih.gov/protein/238846122?report=genbank&log$=protalign&blast_rank=2&RID=BYSSN7VT014), 82% identify) | Pc21g12620 |
|  |  |  | KF25_6509 | nonribosomal peptide synthetase, similarity with protein in *Arthroderma otae* CBS 113480 ([gb\|EEQ35783.1](http://www.ncbi.nlm.nih.gov/protein/238846121?report=genbank&log$=protalign&blast_rank=2&RID=BYSSN7VT014), 70% identify) | Pc21g12630 |
|  |  |  | KF25_6510 | C6 transcription factor, similarity with protein in *Penicillium digitatum* Pd1 ( [gb\|EKV16897.1](http://www.ncbi.nlm.nih.gov/protein/425778792?report=genbank&log$=protalign&blast_rank=2&RID=BYSSN7VT014), 86% identify) | Pc21g12640 |
| 22**^e^** | t1PKS-NRPS |  | KF25_6609 | rho-type GTPase-activating protein, similarity with protein in *Aspergillus oryzae* RIB40 ([dbj\|BAE60603.1](http://www.ncbi.nlm.nih.gov/protein/83770470?report=genbank&log$=protalign&blast_rank=2&RID=BZY7RH0R014), 76% identify) | Pc12g00340 |
|  |  |  | KF25_6610 | MFS multidrug transporter, similarity with protein in *Aspergillus niger* CBS 513.88 (emb\|CAL00457.1, 90% identify) | Pc12g00350 |
|  |  |  | KF25_6611 | polyketide synthase, similarity with protein in *Metarhizium acridum* CQMa 102 (gb\|EFY84397.1, 54% identify) |  |
|  |  |  | KF25_6612 | alcohol dehydrogenase GroES-like domain-containing protein, similarity with protein in *Aspergillus kawachii* IFO 4308 (dbj\|GAA83966.1,94% identify) | Pc18g00530 |
|  |  |  | KF25_6613 | amino acid transporter, similarity with protein in *Trichoderma reesei* QM6a (gb\|EGR49472.1, 47% identify) | Pc12g00380 |
| 23 | t1PKS |  | KF25_7184 | C6 transporter, similarity with protein in *Penicillium griseofulvum* (gb\|ACR02672.1, 78% identify) | Pc22g08140 |
|  |  |  | KF25_7185 | cytochrome P450 oxidoreductase, similarity with protein in *Penicillium griseofulvum* (gb\|ADF47134.1, 92% identify) | Pc22g08150/Pc22g08160 |
|  |  |  | KF25_7186 | 6-methylsalicylic acid synthase, similarity with protein in *Penicillium griseofulvum* (emb\|CAA39295.1, 89% identify) | Pc22g08170 |
|  |  |  | KF25_7187 | diacylglycerol kinase, similarity with protein in *Oxytricha trifallax* (EJY86314.1, 33% identify) | Pc12g03460 |
| 24 | t3PKS |  | KF25_7299 | 3-hydroxyacyl-CoA dehydrogenase, similarity with protein in *Aspergillus flavus*  NRRL3357 (gb\|EED48713.1, 54% identify) | Pc22g09670 |
|  |  |  | KF25_7300 | 6-phosphogluconolactonase, similarity with protein in *Aspergillus kawachii* IFO 4308 (dbj\|GAA83587.1, 54% identify) | Pc22g09680 |
|  |  |  | KF25_7302 | amidotransferase, similarity with protein in *Schizosaccharomyces pombe* 972h- (ref\|NP_592932.1, 30% identify) | Pc22g09700, |
|  |  |  | KF25_7303 | taurine catabolism dioxygenase, similarity with protein in *Aspergillus*  *oryzae* RIB40 (dbj\|BAE57522.1, 82% identify) | Pc22g09710 |
|  |  |  | KF25_7304 | anaphase-promoting complex subunit ApcB, similarity with protein in *Penicillium digitatum* Pd1 (gb\|EKV04122.1, 89% identify) | Pc22g09720 |
| 25 | other |  | KF25_7637 | Mannosyl-oligosaccharide glucosidase, similarity with protein in *Penicillium digitatum* PHI26 (gb\|EKV12214.1, 92% identi | Pc20g12700 |
|  |  |  | KF25_7638 | fad NAD binding oxidoreductase, similarity with protein in *Aspergillus flavus* NRRL3357 (gb\|EED51334.1, 72% identify) | Pc20g12690 |
|  |  |  | KF25_7639 | phenyloxazoline synthase, similarity with protein in *Penicillium digitatum* PHI26 (gb\|EKV19586.1, 67% identify) | Pc20g12670 |
|  |  |  | KF25_7640 | alpha-glucosidase, similarity with protein in *Aspergillus oryzae* RIB40 (dbj\|BAE65599.1, 87% identify) | Pc20g12660/ Pc20g12650 |
|  |  |  | KF25_7641 | MFS maltose permease, similarity with protein in *Aspergillus clavatus* NRRL 1 (gb\|EAW15332.1, 84% identify) | Pc20g12640 |
|  |  |  | KF25_7642 | Killer toxin sensitivity protein, similarity with protein in *Penicillium*  *digitatum* PHI26 (gb\|EKV12217.1, 94% identify) | Pc20g12630 |
|  |  |  | KF25_7643 | Protein transport protein sec22, similarity with protein in *Penicillium digitatum* PHI26 (gb\|EKV12218.1, 97% identify) | Pc20g12620 |
| 26**^f^** | NRPS |  | KF25_8397 | ABC multidrug transporter, similarity with protein in *Aspergillus clavatus* NRRL 1 (gb\|EAW15287.1, 65% identify) | Pc18g02530 |
|  |  |  | KF25_8398 | nonribosomal peptide synthase, similarity with protein in *Aspergillus clavatus* NRRL 1 (gb\|EAW15286.1, 48% identify) | Pc13g14330 |
|  |  |  | KF25_8399 | NACHT domain protein, similarity with protein in *Aspergillus kawachii* IFO 4308 (dbj\|GAA89050.1, 49% identify) | Pc13g14340 |
|  |  |  | KF25_8400 | cytosine deaminase, similarity with protein in *Aspergillus oryzae* 3.042 (gb\|EIT72932.1, 75% identify) | Pc13g14360 |
|  |  |  | KF25_8401 | delta-9 fatty acid desaturase, similarity with protein in *Leptosphaeria maculans* JN3 (emb\|CBX90850.1, 74% identify) | Pc13g14370 |
|  |  |  | KF25_8402 | monooxygenase, similarity with protein in *Aspergillus niger* ATCC 1015 (gb\|EHA19535.1, 42% identify) | Pc13g14380 |
|  |  |  | KF25_8403 | aldose 1-epimerase, similarity with protein in *Penicillium digitatum* PHI26 (gb\|EKV16245.1, 91% identify) | Pc13g14400 |
| 27 | t1PKS |  | KF25_8922 | 3-dehydroshikimate dehydratase, similarity with protein in *Marssonina brunnea f. sp. 'multigermtubi'* MB_m1 (gb\|EKD13365.1, 38% identify) | Pc21g16040 |
|  |  |  | KF25_8923 | Flavin dependent monooxygenase, similarity with protein in *Penicillium digitatum* PHI26 (gb\|EKV10887.1, 85% identify) | Pc21g16030 |
|  |  |  | KF25_8924 | HDA1 complex subunit, similarity with protein in *Aspergillus flavus* NRRL3357 (gb\|EED47974.1, 57% identify) | Pc21g16020 |
|  |  |  | KF25_8925 | Conidial pigment polyketide synthase, similarity with protein in *Penicillium digitatum* PHI26 (gb\|EKV10885.1, 92% identify) | Pc21g16000 |
|  |  |  | KF25_8927 | exopolyphospha tase, similarity with protein in *Penicillium digitatum* PHI26 (gb\|EKV10882.1, 88% identify) | Pc21g15970 |
|  |  |  | KF25_8928 | 1-aminocyclopropane-1-carboxylate deaminase, similarity with protein in *Penicillium digitatum* PHI26 (gb\|EKV10881.1, 86% identify) | Pc21g15960 |
| 28**^g^** | NRPS-Terpene | fumitremorgin | KF25_8965 | protein phosphatase regulatory subunit, similarity with protein in *Penicillium digitatum* PHI26 (gb\|EKV10847.1, 95% identify) | Pc21g15490 |
|  |  |  | KF25_8966 | nonribosomal peptide synthase, similarity with protein in *Neosartorya fischeri* NRRL 181 (gb\|EAW17510.1, 60% identify) | Pc21g15480 |
|  |  |  | KF25_8967 | Cytochrome P450 oxidoreductase, similarity with protein in *Neosartorya fischeri* NRRL 181 (gb\|EAW17509.1, 72% identify) | Pc21g15470 |
|  |  |  | KF25_8968 | MAK1-like monooxygenase, similarity with protein in Neosartorya fischeri NRRL 181 (gb\|EAW16447.1, 48% identify) | Pc21g15460 |
|  |  |  | KF25_8969 | cytochrome P450 monooxygenase, similarity with protein in *Neosartorya fischeri* NRRL 181 (gb\|EAW19755.1, 66% identify) | Pc21g15450 |
| 29 | Terpene |  | KF25_9093 | farnesyl-diphosphate farnesyltransferase, similarity with protein in *Aspergillus nidulans* FGSC A4 (tpe\|CBF82833.1, 78% identify) | Pc21g13930 |
|  |  |  | KF25_9100 | Anthranilate synthase, aminase component, similarity with protein in *Campylobacter showae* CSUNSWCD (gb\|EKU11059.1, 26% identify) | Pc21g13850 |
|  |  |  | KF25_9101 | Mannosyltransferase (PIG-M), similarity with protein in *Penicillium digitatum* Pd1 (gb\|EKV11696.1, 90% identify) | Pc21g13840 |
|  |  |  | KF25_9102 | secreted protein, similarity with protein in *Verticillium dahliae* VdLs.17 (gb\|EGY13653.1, 63% identify) | Pc13g08250 |
|  |  |  | KF25_9103 | Adenylyl-sulfate kinase, similarity with protein in *Penicillium digitatum* Pd1 (gb\|EKV17582.1, 90% identify) | Pc13g08250 |
| 30**^h^** | NRPS | Penicillin | KF25_9291 | Peptidase S41 family protein, similarity with protein in *Aspergillus flavus* NRRL3357 (gb\|EED45472.1, 44% identify) | Pc21g21410/Pc21g21420 |
|  |  |  | KF25_9292 | alpha-aminoadypil-cysteinyl-valine synthetase, similarity with protein in *Penicillium chrysogenum* Wisconsin 54-1255 (gb\|ABA70582.1, 98% identify) |  |
|  |  |  | KF25_9293 | isopenicillin N synthase ips/PcbC, similarity with protein in *Penicillium chrysogenum* Wisconsin 54-1255 (emb\|CAP97035.1, 99% identify) | Pc21g21380 |
|  |  |  | KF25_9294 | acyl-coenzyme A:isopenicillin N acyltransferase (acyltransferase)  AAT/PenDE, similarity with protein in *Penicillium chrysogenum* Wisconsin 54-1255 (XP_002569112, 99% identify) | Pc21g21370 |
|  |  |  | KF25_9295 | FAD dependent oxidoreductase, similarity with protein in *Penicillium chrysogenum* Wisconsin 54-1255 (ABR12618, 99% identify) | Pc21g21350 |
|  |  |  | KF25_9296 | saccharopine dehydrogenase, similarity with protein in *Acinetobacter sp*. NCTC 10304 (ref\|ZP_10937437.1, 45% identify) | Pc21g21340 |
| 31**^i^** | NRPS |  | KF25_9346 | ABC multidrug transporter, similarity with protein in *Penicillium digitatum* PHI26 (gb\|EKV12763.1, 89% identify) | Pc16g04670 |
|  |  |  | KF25_9347 | Nonribosomal peptide synthase Pes1, similarity with protein in *Penicillium digitatum* Pd1 (gb\|EKV21467.1, 89% identify) | Pc16g04690 |
|  |  |  | KF25_9348 | MFS multidrug transporter, similarity with protein in *Penicillium digitatum* PHI26 (gb\|EKV12761.1, 92% identify) | Pc16g04700 |
|  |  |  | KF25_9351 | phosphoglycerate kinase pgkA, similarity with protein in *Penicillium*  *chrysogenum* Wisconsin 54-1255 (emb\|CAP93143.1, 99% identify) | Pc16g04730 |
| 32 | t1PKS |  | KF25_9358 | Pyridoxal reductase (AKR8), similarity with protein in *Penicillium digitatum* PHI26 (gb\|EKV12751.1, 90% identify) | Pc16g04830 |
|  |  |  | KF25_9359 | phytanoyl-CoA hydroxylase, similarity with protein in *Exophiala dermatitidis* NIH/UT8656 (gb\|EHY58852.1, 61% identify) | Pc16g04850 |
|  |  |  | KF25_9360 | cytochrome P450, similarity with protein in *Glomerella graminicola* M1.001 (gb\|EFQ34281.1, 39% identify) | Pc16g04880/ Pc16g04870 |
|  |  |  | KF25_9361 | polyketide synthase, similarity with protein in *Aspergillus niger* CBS 513.88 (ref\|XP_001397040.2, 68% identify) | Pc16g04890 |
|  |  |  | KF25_9363 | MFS transporter, similarity with protein in *Aspergillus niger* CBS 513.88 (ref\|XP_001397037.1, 77% identify) | Pc16g04910 |
| 33 | t1PKS-NRPS |  | KF25_9452 | polyketide synthase, similarity with protein in *Aspergillus kawachii* IFO 4308 (dbj\|GAA83831.1, 55% identify) | Pc16g03800 |
|  |  |  | KF25_9453 | integral membrane protein, similarity with protein in *Aspergillus kawachii* IFO 4308 (dbj\|GAA91403.1, 56% identify) | Pc16g03810 |
|  |  |  | KF25_9456 | peptide synthetase, similarity with protein in *Ajellomyces dermatitidis* ATCC 18188 (gb\|EGE78437.1, 56% identify) | Pc16g03850 |
|  |  |  | KF25_9457 | aerobactin siderophore biosynthesis protein, similarity with protein in *Arthroderma gypseum* CBS 118893 (gb\|EFR01377.1, 58% identify) | Pc16g03860 |
|  |  |  | KF25_9458 | multidrug resistance protein/ABC multidrug transporter, similarity with protein in *Aspergillus oryzae* RIB40 (XP_001823188.2, 77% identify) | Pc16g03870 |





**Supplemental Figure 6. Putative structures of the predicted secondary metabolism gene clusters products.** The letters for each structure were corresponded with the letter in Supplemental Table 7. All structures were predicted by using antiSMASH.


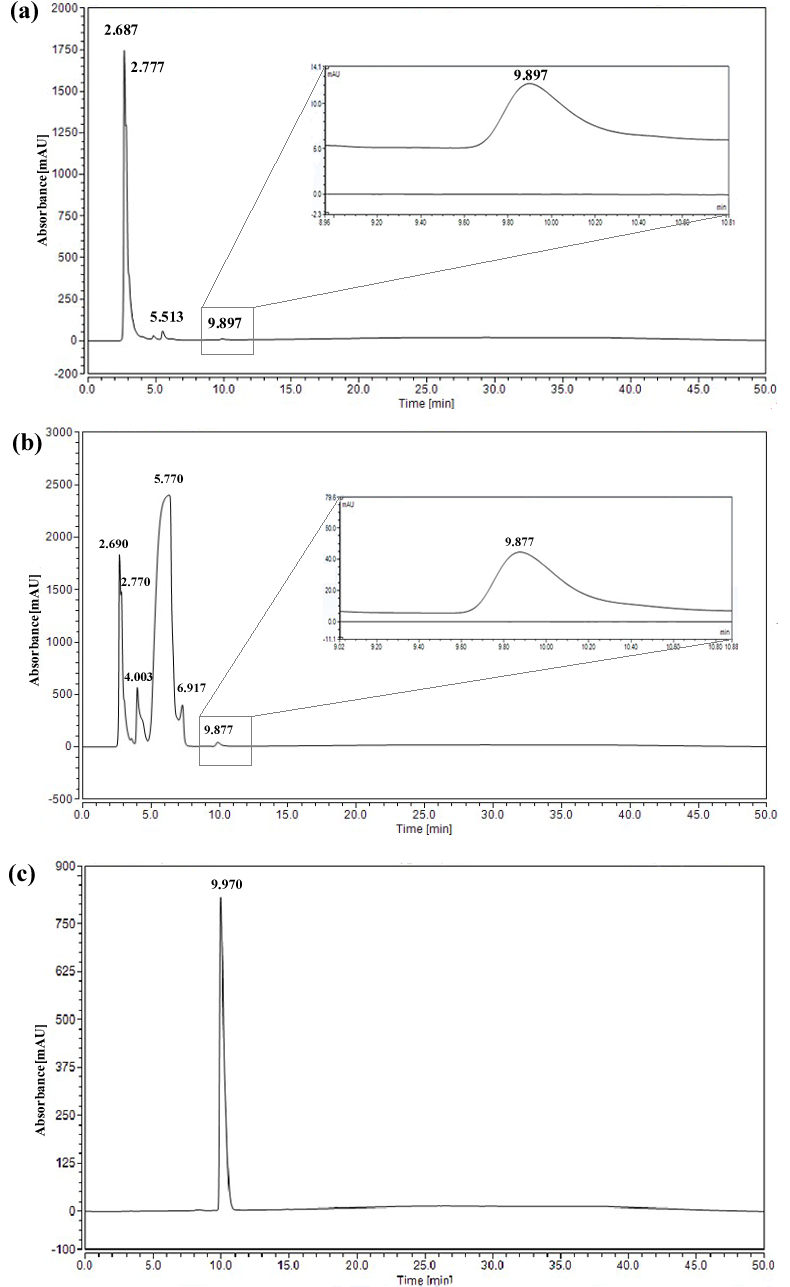


**Supplemental Figure 7. Detection of penicillin G by HPLC-DAD.** The formation of penicillin G by *P. chrysogenum* KF-25 (A) and *P. chrysogenum* Wisconsin 54-1255 (B) were detected by HPLC-DAD at wavelength of 210 nm. The peak of penicillin G was indicated. Penicillin G (0.5mg/ml) was used as positive control (C).


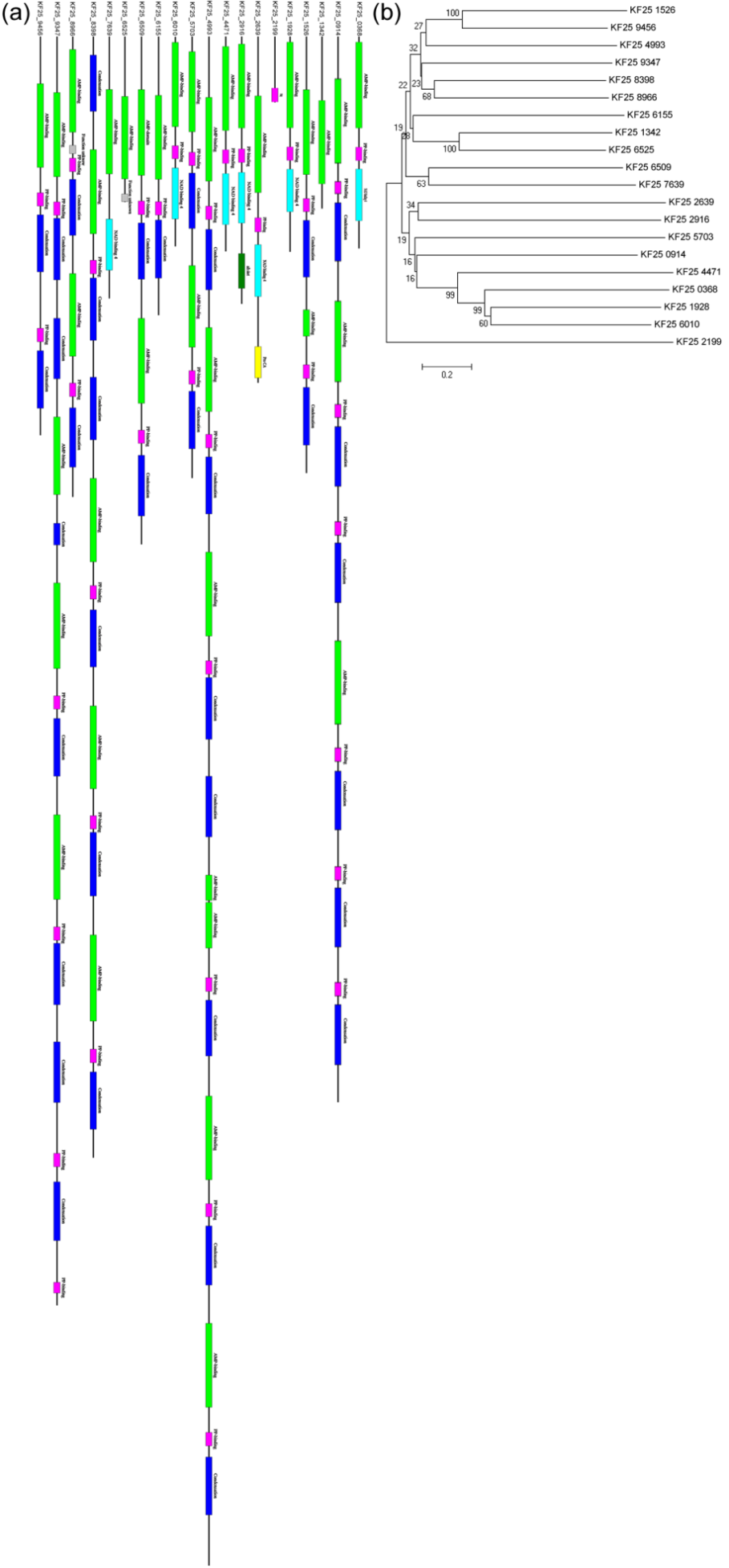


**Supplemental Figure 8. The domain compositions and the** **phylogenetic tree of the non-ribosomal synthetases from KF-25 genome. (a)** Totally 20 non-ribosomal synthetases were found in KF-25 genome and the domain compositions were identified by using Pfam [90]. Green rectangle indicated the AMP-binding enzyme, blue rectangle indicated condensation domain, wathet blue rectangle indicated NAD binding 4 domain (described as male sterility protein), purple rectangle indicated PP-binding domain (described as phosphopantetheine attachment site), yellow rectangle indicated Pro CA domain (described as carbonic anhydrase) and gray rectangle indicated domains with function unknown. (b) The Neighor-Joining phylogenetic tree of the non-ribosomal synthetase from KF-25 was constructed by using MEGA 5.05 [91] with neighbor-joining method and bootstrap analysis (1,000 replicates) of a Muscle alignment of the non-ribosomal synthetase amino acid sequences.


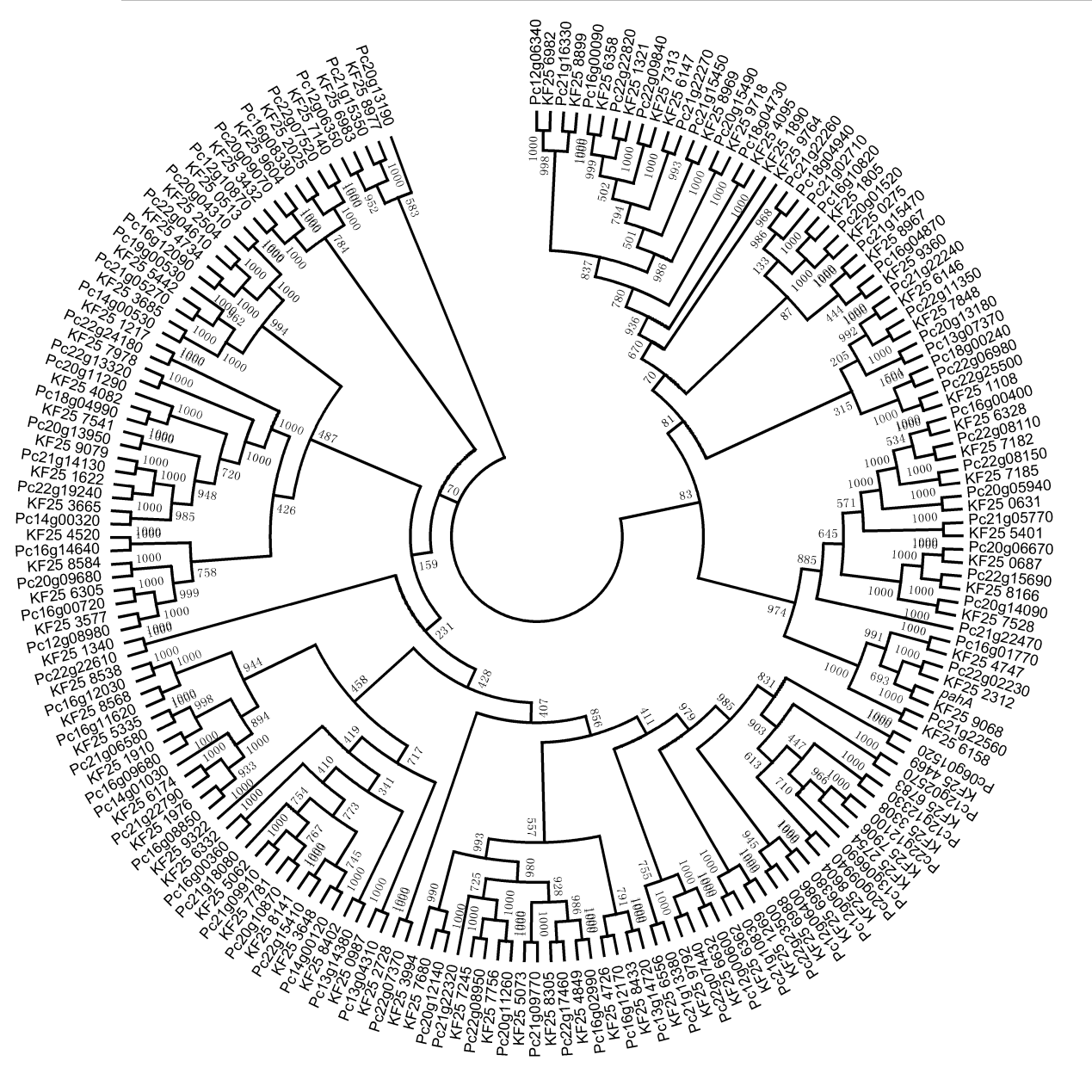


**Supplemental Figure 9. Neighor-Joining (NJ) phylogenetic tree of the cytochrome P450 (CYPs) from the genomes of *P. chrysogenum* KF-25 and *P. chrysogenum* Wisconsin 54-1255.** The CYP protein amino acid sequences from the genomes of KF-25 and Wisconsin 54-1255 were aligned by using ClustalX2.1 and the NJ tree were also constructed by using Clustal X2.1 [86]. As shown in the phylogenetic tree, the classifications of the CYPs from both the two genomes were almost the same.

**Supplemental Table 8. Primers used to amplify the *P. chrysogenum* Wisconsin 54-1255 specific genes from both the genomes of *P. chrysogenum* Wisconsin 54-1255 and *P. chrysogenum* KF-25.**

| Primers | Sequence | PCR product (length, positions in Wisconsin 54-1255 genome) | Function of the fragemnts or the encoding proteins |
| --- | --- | --- | --- |
| 03g00290_For | CTTTGTTGTAGTTCAGAGGGTC | Pc03g00290 (794bp, 70185..70978) | hypothetical protein |
| 03g00290_Rev | CGCACTGGGCAGGAGTAA |  |  |
| 12g02270_For | GACCCAATCTCCACCACT | Pc12g02270 (1443bp, 1332318..1333760) | hypothetical protein |
| 12g02270_Rev | ATAGCCTCAACCTCCATC |  |  |
| 21g20980_For | ATGGGAAATGTGAAAGGT | Pc21g20980 (884bp, 24157350..24158232) | hypothetical protein |
| 21g20980_Rev | TAGGTGCTGTTGGTGTTG |  |  |
| Pc00c02_For | CTGCGATTACTAGCGACTCCGACTT | Pc00c02 (1208bp, 9093..10300) | 16s rDNA |
| Pc00c02_Rev | AACCTGTCCTTGGCTTTGG |  |  |

**Supplemental Table 9. Orthologous genes used in phylogenetic analysis of various filamentous fungi.**

| *P. chrysogenum*  *Wisconsin 54-1255* | *P. marneffei* | *T. stipitatus* | *N. fischeri* | *A. clavatus* | *A. terreus* | *A. nidulans* | *A. oryzae* | Protein name |
| --- | --- | --- | --- | --- | --- | --- | --- | --- |
| Pc22g25570 | PMAA_051060 | EED17124.1 | NFIA_021750 | ACLA_031360 | ATEG_02520 | AN0272.2 | AO090005000779 | Phosphotransferase enzyme family domain protein |
| Pc22g24340 | PMAA_013140 | EED19372.1 | NFIA_048320 | ACLA_098430 | ATEG_07240 | AN8676.2 | AO090120000095 | SRF-type transcription factor (Umc1), putative |
| Pc22g23790 | PMAA_018420 | EED19334.1 | NFIA_048700 | ACLA_098030 | ATEG_07208 | AN8704.2 | AO090120000131 | 60S ribosomal protein L24a |
| Pc22g20960 | PMAA_047670 | EED20525.1 | NFIA_085890 | ACLA_069530 | ATEG_01520 | AN9470.2 | AO090011000588 | urate oxydase UaZ |
| Pc22g20850 | PMAA_048090 | EED20583.1 | NFIA_085960 | ACLA_069600 | ATEG_01513 | AN5971.2 | AO090011000578 | NADH-ubiquinone oxidoreductase 299 kDa subunit, putative |
| Pc22g19950 | PMAA_087050 | EED18506.1 | NFIA_085350 | ACLA_068990 | ATEG_04429 | AN6014.2 | AO090011000642 | fatty acid activator Faa4, putative |
| Pc22g18450 | PMAA_027020 | EED23791.1 | NFIA_108970 | ACLA_046570 | ATEG_05486 | AN4434.2 | AO090023000850 | 37S ribosomal protein Rsm25 |
| Pc22g17740 | PMAA_027900 | EED23680.1 | NFIA_109510 | ACLA_045950 | ATEG_05558 | AN4382.2 | AO090023000914 | ATP binding protein |
| Pc22g15750 | PMAA_084050 | EED18148.1 | NFIA_079330 | ACLA_012550 | ATEG_08330 | AN7736.2 | AO090701000709 | C2H2 finger and ankyrin domain protein, putative |
| Pc22g13980 | PMAA_083490 | EED18067.1 | NFIA_038120 | ACLA_001180 | ATEG_08011 | AN8253.2 | AO090102000602 | proteasome maturation ans ribosome synthesis protein Nop10, putative |
| Pc22g13280 | PMAA_073000 | EED22096.1 | NFIA_054060 | ACLA_082260 | ATEG_05813 | AN3923.2 | AO090001000497 | ubiquitin-protein ligase E3 component (UBR1), putative |
| Pc22g12080 | PMAA_097010 | EED14938.1 | NFIA_106090 | ACLA_049180 | ATEG_00747 | AN2055.2 | AO090003001238 | conserved hypothetical protein |
| Pc22g12070 | PMAA_097100 | EED14927.1 | NFIA_106100 | ACLA_049170 | ATEG_00748 | AN2056.2 | AO090003001239 | conserved hypothetical protein |
| Pc22g05310 | PMAA_042140 | EED24271.1 | NFIA_104090 | ACLA_051170 | ATEG_03851 | AN3649.2 | AO090003000992 | 50S ribosomal protein L2 |
| Pc22g05070 | PMAA_041960 | EED24250.1 | NFIA_104300 | ACLA_050970 | ATEG_03872 | AN3629.2 | AO090003001022 | formamidopyrimidine-DNA glycosylase, putative |
| Pc22g04060 | PMAA_080100 | EED17655.1 | NFIA_058290 | ACLA_085580 | ATEG_03285 | AN3737.2 | AO090009000186 | WD domain protein |
| Pc22g02350 | PMAA_077930 | EED17400.1 | NFIA_059110 | ACLA_086430 | ATEG_03352 | AN5493.2 | AO090003000443 | NupC family nucleoside cotransporter |
| Pc22g00860 | PMAA_062850 | EED21006.1 | NFIA_059530 | ACLA_086870 | ATEG_03406 | AN5440.2 | AO090103000329 | cytochrome c peroxidase, putative |
| Pc21g23610 | PMAA_075850 | EED22377.1 | NFIA_008390 | ACLA_018520 | ATEG_05095 | AN0632.2 | AO090005000976 | mRNA splicing factor (Prp18), putative |
| Pc21g22200 | PMAA_066740 | EED21448.1 | NFIA_008900 | ACLA_019050 | ATEG_05152 | AN0956.2 | AO090005001057 | dihydrouridine synthase family protein, putative |
| Pc21g20430 | PMAA_035610 | EED13156.1 | NFIA_056370 | ACLA_084330 | ATEG_02371 | AN2441.2 | AO090023000210 | ubiquitin-like activating enzyme (UlaA), putative |
| Pc21g18850 | PMAA_080910 | EED17750.1 | NFIA_039360 | ACLA_002420 | ATEG_09241 | AN8119.2 | AO090102000391 | alpha-1,2-mannosyltransferase (Ktr4), putative |
| Pc21g18830 | PMAA_080890 | EED17748.1 | NFIA_039340 | ACLA_002400 | ATEG_09244 | AN8117.2 | AO090102000393 | fatty acid elongase (Gns1), putative |
| Pc21g18000 | PMAA_084730 | EED18216.1 | NFIA_079930 | ACLA_010590 | ATEG_09344 | AN2238.2 | AO090701000219 | DnaJ domain protein Psi, putative |
| Pc21g15910 | PMAA_082190 | EED17897.1 | NFIA_038640 | ACLA_001700 | ATEG_08067 | AN8215.2 | AO090102000557 | methylenetetrahydrofolate reductase |
| Pc21g14680 | PMAA_081450 | EED17799.1 | NFIA_040040 | ACLA_003190 | ATEG_09826 | AN8049.2 | AO090003001313 | NADH-ubiquinone oxidoreductase subunit, putative |
| Pc21g12230 | PMAA_010420 | EED12653.1 | NFIA_114740 | ACLA_065510 | ATEG_01829 | AN9108.2 | AO090038000578 | indoleamine 2,3-dioxygenase family protein |
| Pc21g11500 | PMAA_011110 | EED12577.1 | NFIA_115310 | ACLA_064960 | ATEG_01886 | AN9057.2 | AO090311000001 | conserved hypothetical protein |
| Pc21g11120 | PMAA_060240 | EED16018.1 | NFIA_096990 | ACLA_057610 | ATEG_00039 | AN1442.2 | AO090103000044 | translocation protein (Sec66), putative |
| Pc21g10360 | PMAA_073380 | EED22129.1 | NFIA_076460 | ACLA_013770 | ATEG_07632 | AN2751.2 | AO090010000464 | topisomerase II associated protein (Pat1), putative |
| Pc21g08790 | PMAA_088450 | EED18644.1 | NFIA_025500 | ACLA_006230 | ATEG_04342 | AN6817.2 | AO090005000137 | alcohol dehydrogenase, zinc-containing |
| Pc21g07860 | PMAA_024930 | EED20119.1 | NFIA_019390 | ACLA_029290 | ATEG_03094 | AN3895.2 | AO090009000410 | CAIB/BAIF family enzyme |
| Pc21g07430 | PMAA_072190 | EED22025.1 | NFIA_074990 | ACLA_015280 | ATEG_02048 | AN0075.2 | AO090120000344 | disulfide isomerase (TigA), putative |
| Pc21g07110 | PMAA_031190 | EED23254.1 | NFIA_075300 | ACLA_015010 | ATEG_02076 | AN0105.2 | AO090120000318 | eukaryotic translation initation factor eIF1a-like protein, putative |
| Pc21g06870 | PMAA_030980 | EED23277.1 | NFIA_075520 | ACLA_014820 | ATEG_02094 | AN0121.2 | AO090120000294 | hydroxymethylbilane synthase, putative |
| ﻿Pc21g06450 | PMAA_030790 | EED23301.1 | NFIA_075690 | ACLA_014650 | ATEG_02113 | AN0138.2 | AO090026000670 | transcription factor TFIIH subunit Tfb4, putative |
| Pc21g06050 | PMAA_033110 | EED22978.1 | NFIA_076130 | ACLA_014180 | ATEG_07684 | AN0191.2 | AO090026000732 | PAXNEB protein superfamily |
| Pc21g04880 | PMAA_069350 | EED21715.1 | NFIA_024940 | ACLA_005680 | ATEG_04279 | AN4259.2 | AO090026000816 | PCI domain protein |
| Pc21g04340 | PMAA_069220 | EED21702.1 | NFIA_025290 | ACLA_006020 | ATEG_04313 | AN5675.2 | AO090005000117 | UBX domain protein (Ubx5), putative |
| Pc21g04300 | PMAA_071930 | EED22004.1 | NFIA_074670 | ACLA_015590 | ATEG_02015 | AN0056.2 | AO090120000378 | arrestin (or S-antigen), N-terminal domain protein |
| Pc21g03150 | PMAA_036900 | EED13017.1 | NFIA_073060 | ACLA_016910 | ATEG_06135 | AN6920.2 | AO090113000079 | SNF7 family protein |
| Pc20g14430 | PMAA_092220 | EED15515.1 | NFIA_087310 | ACLA_071010 | ATEG_01149 | AN6312.2 | AO090026000433 | conserved hypothetical protein |
| Pc20g14410 | PMAA_092200 | EED15517.1 | NFIA_087290 | ACLA_070990 | ATEG_01147 | AN10806.4 | AO090026000435 | YagE family protein |
| Pc20g13910 | PMAA_091950 | EED15540.1 | NFIA_087050 | ACLA_070760 | ATEG_01121 | AN6200.2 | AO090026000462 | pre-rRNA processing protein Rrp12, putative |
| Pc20g13010 | PMAA_017930 | EED19281.1 | NFIA_050480 | ACLA_096610 | ATEG_07054 | AN6614.2 | AO090701000148 | phospholipid-translocating P-type ATPase, putative |
| Pc20g12570 | PMAA_018090 | EED19301.1 | NFIA_050630 | ACLA_096430 | ATEG_07037 | AN6599.2 | AO090701000133 | DUF28 domain protein |
| Pc20g09960 | PMAA_059530 | EED16108.1 | NFIA_097700 | ACLA_058300 | ATEG_00107 | AN1491.2 | AO090005000655 | COP9 signalosome subunit 1 (CsnA), putative |
| Pc20g08410 | PMAA_004640 | EED14083.1 | NFIA_071470 | ACLA_033730 | ATEG_08962 | AN3432.2 | AO090020000042 | aldose 1-epimerase, putative |
| Pc20g05910 | PMAA_021960 | EED19756.1 | NFIA_033510 | ACLA_094520 | ATEG_08153 | AN7659.2 | AO090701000397 | ATP dependent RNA helicase (Dbp5), putative |
| Pc20g04400 | PMAA_087640 | EED18572.1 | NFIA_034500 | ACLA_092690 | ATEG_05703 | AN4592.2 | AO090011000488 | fatty acid desaturase, putative |
| Pc20g03160 | PMAA_003380 | EED13864.1 | NFIA_070650 | ACLA_034560 | ATEG_08758 | AN4774.2 | AO090020000339 | siroheme synthase, putative |
| Pc20g02750 | PMAA_002730 | EED13766.1 | NFIA_070300 | ACLA_034910 | ATEG_08726 | AN4802.2 | AO090020000298 | 60S ribosomal protein L21, putative |
| Pc20g00270 | PMAA_028250 | EED23642.1 | NFIA_110210 | ACLA_045220 | ATEG_06826 | AN4303.2 | AO090023001003 | choline phosphate cytidylyltransferase Muq1, putative |
| Pc18g06270 | PMAA_057530 | EED16351.1 | NFIA_080890 | ACLA_090430 | ATEG_03666 | AN5226.2 | AO090005001538 | GPR/FUN34 family protein |
| Pc18g04290 | PMAA_055910 | EED16568.1 | NFIA_035530 | ACLA_091380 | ATEG_09575 | AN4510.2 | AO090120000268 | cyclic nucleotide-binding domain protein |
| Pc18g04190 | PMAA_055800 | EED16579.1 | NFIA_035460 | ACLA_091730 | ATEG_09581 | AN10563.4 | AO090120000280 | alkaline phosphatase Pho8 |
| Pc18g03540 | PMAA_056420 | EED16500.1 | NFIA_107080 | ACLA_048460 | ATEG_05305 | AN1639.2 | AO090023000652 | thioredoxin, putative |
| Pc18g03350 | PMAA_026280 | EED23865.1 | NFIA_017990 | ACLA_027870 | ATEG_02957 | AN5711.2 | AO090001000465 | RLI and DUF367 domain protein |
| Pc18g02590 | PMAA_055440 | EED16670.1 | NFIA_021070 | ACLA_031030 | ATEG_03104 | AN4053.2 | AO090009000419 | importin 11, putative |
| Pc18g01280 | PMAA_021140 | EED19650.1 | NFIA_065580 | ACLA_039630 | ATEG_04715 | AN2877.2 | AO090003000735 | oxysterol binding protein (Osh5), putative |
| Pc18g00590 | PMAA_023790 | EED19963.1 | NFIA_066580 | ACLA_038600 | ATEG_04600 | AN4918.2 | AO090003000616 | conserved hypothetical protein |
| Pc16g14390 | PMAA_074200 | EED22226.1 | NFIA_010480 | ACLA_020420 | ATEG_00856 | AN0834.2 | AO090005001238 | protein translocation complex componenet (Npl1), putative |
| Pc16g09250 | PMAA_054250 | EED16802.1 | NFIA_020210 | ACLA_030130 | ATEG_03746 | AN0432.2 | AO090003000873 | NADH-cytochrome b5 reductase, putative |
| Pc16g01080 | PMAA_060720 | EED15941.1 | NFIA_096460 | ACLA_042950 | ATEG_10389 | AN8881.2 | AO090010000768 | mitochondrial ornithine carrier protein AmcA/Ort1, putative |
| Pc15g00450 | PMAA_048560 | EED20654.1 | NFIA_086590 | ACLA_070280 | ATEG_01445 | AN5881.2 | AO090026000518 | conserved hypothetical protein |
| Pc15g00160 | PMAA_048980 | EED20687.1 | NFIA_086780 | ACLA_070510 | ATEG_01423 | AN5861.2 | AO090026000492 | ketoreductase, putative |
| Pc14g00060 | PMAA_098980 | EED14727.1 | NFIA_002920 | ACLA_062780 | ATEG_09071 | AN3776.2 | AO090103000095 | MFS transporter, putative |
| Pc13g14200 | PMAA_019530 | EED19458.1 | NFIA_064180 | ACLA_040880 | ATEG_04092 | AN3178.2 | AO090012000818 | deacetylase complex subunit Sds3, putative |
| Pc13g12860 | PMAA_098130 | EED14829.1 | NFIA_013260 | ACLA_023210 | ATEG_00423 | AN1066.2 | AO090001000304 | mitochondrial hypoxia responsive domain protein |
| Pc13g11860 | PMAA_067370 | EED21513.1 | NFIA_012140 | ACLA_022060 | ATEG_00539 | AN0675.2 | AO090012000545 | aflatoxin B1-aldehyde reductase GliO-like, putative |
| Pc13g11340 | PMAA_068180 | EED21607.1 | NFIA_012800 | ACLA_022710 | ATEG_00465 | AN0999.2 | AO090012000638 | adenylyl cyclase-associated protein (cap) |
| Pc13g09990 | PMAA_015820 | EED19040.1 | NFIA_064820 | ACLA_040320 | ATEG_04144 | AN3095.2 | AO090005000720 | conidiophore development protein HymA |
| Pc13g08120 | PMAA_058170 | EED16262.1 | NFIA_107810 | ACLA_047720 | ATEG_05361 | AN1721.2 | AO090023000724 | monosaccharide-P-dolichol utilization protein, putative |
| Pc13g04590 | PMAA_070480 | EED21845.1 | NFIA_083870 | ACLA_080050 | ATEG_09660 | AN6176.2 | AO090011000883 | tubulin-specific chaperone Rbl2, putative |
| Pc13g04560 | PMAA_070630 | EED21850.1 | NFIA_083890 | ACLA_080070 | ATEG_09663 | AN4180.2 | AO090011000881 | MFS transporter, putative |
| Pc13g04480 | PMAA_070660 | EED21854.1 | NFIA_083950 | ACLA_080130 | ATEG_09667 | AN6170.2 | AO090011000874 | DnaJ domain protein, putative |
| Pc13g04040 | PMAA_070920 | EED21891.1 | NFIA_084220 | ACLA_080430 | ATEG_09696 | AN6139.2 | AO090011000843 | 1-acylglycerol-3-phosphate acyltransferase (AtaAp), putative |
| Pc13g03600 | PMAA_071270 | EED21935.1 | NFIA_084530 | ACLA_080810 | ATEG_09879 | AN10472.4 | AO090011000799 | thiamine biosynthetic bifunctional enzyme, putative |
| Pc13g03220 | PMAA_053380 | EED16889.1 | NFIA_021230 | ACLA_031190 | ATEG_02545 | AN0259.2 | AO090005000758 | adenylate kinase Adk2, putative |
| Pc13g02760 | PMAA_019940 | EED19503.1 | NFIA_018480 | ACLA_028290 | ATEG_02990 | AN4232.2 | AO090001000427 | ribosome biogenesis protein Ssf2, putative |
| Pc12g15750 | PMAA_025610 | EED23941.1 | NFIA_067520 | ACLA_037580 | ATEG_04499 | AN5008.2 | AO090005001272 | conserved hypothetical protein |
| Pc12g15510 | PMAA_025870 | EED23917.1 | NFIA_067320 | ACLA_037810 | ATEG_04520 | AN4990.2 | AO090003000529 | vacuolar iron transporter Ccc1, putative |
| Pc12g15080 | PMAA_097710 | EED14854.1 | NFIA_013440 | ACLA_023490 | ATEG_00405 | AN1074.2 | AO090001000337 | glycine cleavage system H protein |
| Pc12g13220 | PMAA_022640 | EED19852.1 | NFIA_089540 | ACLA_073120 | ATEG_01049 | AN2406.2 | AO090023000078 | conserved hypothetical protein |
| Pc12g11370 | PMAA_029770 | EED23438.1 | NFIA_054280 | ACLA_083780 | ATEG_06604 | AN9512.2 | AO090001000524 | mitochondrial protein, putative |
| Pc12g07590 | PMAA_092820 | EED15435.1 | NFIA_088070 | ACLA_071710 | ATEG_01247 | AN6265.2 | AO090026000345 | small nucleolar ribonucleoprotein complex subunit, putative |
| Pc12g07260 | PMAA_076140 | EED22400.1 | NFIA_088310 | ACLA_071950 | ATEG_01283 | AN6244.2 | AO090026000270 | 3' exoribonuclease family protein (Rrp42), putative |
| Pc12g06170 | PMAA_091140 | EED15640.1 | NFIA_026670 | ACLA_007150 | ATEG_06387 | AN6698.2 | AO090005000411 | conserved hypothetical protein |
| Pc12g05480 | PMAA_095290 | EED15130.1 | NFIA_037890 | ACLA_000950 | ATEG_07992 | AN8273.2 | AO090102000625 | ubiquinol-cytochrome C reductase complex core protein 2, putative |
| Pc12g04760 | PMAA_011490 | EED12531.1 | NFIA_051780 | ACLA_095330 | ATEG_05654 | AN6500.2 | AO090701000014 | 60S ribosomal protein L28 |

Refer to **Supplemental Table 9** (continued).

| *A. niger* | *G. zeae* | *A. fumigatus* | *P. digitatum* | *A. flavus* | *A. kawachii* | *P. chrysogenum* KF-25 | Protein name |
| --- | --- | --- | --- | --- | --- | --- | --- |
| An01g05030 | XP_380532.1 | AFUA_1G02880 | EKV13527.1 | XP_002372945.1 | GAA83455.1 | KF25_1101 | Phosphotransferase enzyme family domain protein |
| An12g08730 | XP_388872.1 | AFUA_6G02110 | EKV15272.1 | XP_002380911.1 | GAA87728.1 | KF25_1204 | SRF-type transcription factor (Umc1), putative |
| An12g07830 | XP_388852.1 | AFUA_6G02440 | EKV15308.1 | XP_002380945.1 | GAA87672.1 | KF25_1247 | 60S ribosomal protein L24a |
| An02g06030 | XP_384302.1 | AFUA_2G10520 | EKV14875.1 | XP_002377871.1 | GAA82145.1 | KF25_1477 | urate oxydase UaZ |
| An02g05880 | XP_382117.1 | AFUA_2G10600 | EKV14884.1 | XP_002377863.1 | GAA82153.1 | KF25_1485 | NADH-ubiquinone oxidoreductase 299 kDa subunit, putative |
| An16g05150 | XP_388719.1 | AFUA_2G09910 | EKV14958.1 | XP_002377926.1 | GAA85077.1 | KF25_1563 | fatty acid activator Faa4, putative |
| An04g01650 | XP_381829.1 | AFUA_4G07250 | EKV19697.1 | XP_002377129.1 | GAA82645.1 | KF25_1681 | 37S ribosomal protein Rsm25 |
| An04g01100 | XP_390498.1 | AFUA_4G06710 | EKV19608.1 | XP_002377192.1 | GAA82593.1 | KF25_8323 | ATP binding protein |
| An03g04080 | XP_389021.1 | AFUA_5G07960 | EKV14451.1 | XP_002378918.1 | GAA91314.1 | KF25_8169 | C2H2 finger and ankyrin domain protein, putative |
| An09g06180 | XP_389213.1 | AFUA_5G04000 | EKV14245.1 | XP_002382875.1 | GAA88208.1 | KF25_8029 | proteasome maturation ans ribosome synthesis protein Nop10, putative |
| An11g01610 | XP_381370.1 | AFUA_6G08420 | EKV13305.1 | XP_002381990.1 | GAA84056.1 | KF25_7974 | ubiquitin-protein ligase E3 component (UBR1), putative |
| An04g07090 | XP_386912.1 | AFUA_4G09960 | EKV13112.1 | XP_002374102.1 | GAA91722.1 | KF25_7904 | conserved hypothetical protein |
| An04g07080 | XP_386925.1 | AFUA_4G09950 | EKV13113.1 | XP_002374101.1 | GAA91723.1 | KF25_7903 | conserved hypothetical protein |
| An01g07430 | XP_381466.1 | AFUA_4G12170 | EKV12835.1 | XP_002374334.1 | GAA83646.1 | KF25_2561 | 50S ribosomal protein L2 |
| An01g07220 | XP_388627.1 | AFUA_4G11930 | EKV07371.1 | XP_002374305.1 | GAA83627.1 | KF25_2542 | formamidopyrimidine-DNA glycosylase, putative |
| An06g01480 | XP_388025.1 | AFUA_6G12330 | EKV07453.1 | XP_002383396.1 | GAA87991.1 | KF25_2460 | WD domain protein |
| An08g10300 | XP_390707.1 | AFUA_6G13190 | EKV07181.1 | XP_002374885.1 | GAA87915.1 | KF25_2324 | NupC family nucleoside cotransporter |
| An08g08720 | XP_390782.1 | AFUA_6G13570 | EKV07049.1 | XP_002385147.1 | GAA83119.1 | KF25_2208 | cytochrome c peroxidase, putative |
| An01g09860 | XP_382709.1 | AFUA_1G16990 | EKV18842.1 | XP_002373145.1 | GAA82047.1 | KF25_6240 | mRNA splicing factor (Prp18), putative |
| An01g10700 | XP_387393.1 | AFUA_1G16550 | EKV11997.1 | XP_002373218.1 | GAA87387.1 | KF25_9232 | dihydrouridine synthase family protein, putative |
| An11g00460 | XP_384444.1 | AFUA_6G10600 | EKV17877.1 | XP_002376511.1 | GAA84142.1 | KF25_5867 | ubiquitin-like activating enzyme (UlaA), putative |
| An02g09940 | XP_387546.1 | AFUA_5G02740 | EKV14705.1 | XP_002382695.1 | GAA84561.1 | KF25_0090 | alpha-1,2-mannosyltransferase (Ktr4), putative |
| An02g09910 | XP_387523.1 | AFUA_5G02760 | EKV14703.1 | XP_002382698.1 | GAA84563.1 | KF25_0088 | fatty acid elongase (Gns1), putative |
| An17g00770 | XP_385309.1 | AFUA_5G07340 | EKV14648.1 | XP_002378424.1 | GAA89796.1 | KF25_0023 | DnaJ domain protein Psi, putative |
| An09g05860 | XP_387303.1 | AFUA_5G03480 | EKV10875.1 | XP_002382828.1 | GAA88238.1 | KF25_8933 | methylenetetrahydrofolate reductase |
| An02g11200 | XP_387085.1 | AFUA_5G02080 | EKV11019.1 | XP_002374032.1 | GAA84468.1 | KF25_9032 | NADH-ubiquinone oxidoreductase subunit, putative |
| An12g00660 | XP_389216.1 | AFUA_7G02010 | EKV16913.1 | XP_002380677.1 | GAA91145.1 | KF25_6480 | indoleamine 2,3-dioxygenase family protein |
| An09g03040 | XP_380881.1 | AFUA_7G02600 | EKV16784.1 | XP_002372796.1 | GAA91616.1 | KF25_6421 | conserved hypothetical protein |
| An16g08830 | XP_381391.1 | AFUA_8G04260 | EKV11880.1 | XP_002385427.1 | GAA89371.1 | KF25_6387 | translocation protein (Sec66), putative |
| An14g03890 | XP_389839.1 | AFUA_5G10770 | EKV11796.1 | XP_002384654.1 | GAA90799.1 | KF25_5026 | topisomerase II associated protein (Pat1), putative |
| An13g01120 | XP_391096.1 | AFUA_7G04530 | EKV11676.1 | XP_002372297.1 | GAA89677.1 | KF25_5149 | alcohol dehydrogenase, zinc-containing |
| An18g04200 | XP_383842.1 | AFUA_1G05360 | EKV15674.1 | XP_002383194.1 | GAA83423.1 | KF25_5230 | CAIB/BAIF family enzyme |
| An18g02020 | XP_387356.1 | AFUA_5G12260 | EKV15607.1 | XP_002381148.1 | GAA83239.1 | KF25_5263 | disulfide isomerase (TigA), putative |
| An18g02370 | XP_389830.1 | AFUA_5G11985 | EKV15528.1 | XP_002381121.1 | GAA83265.1 | KF25_5289 | eukaryotic translation initation factor eIF1a-like protein, putative |
| An18g02970 | XP_389153.1 | AFUA_5G11760 | EKV15438.1 | XP_002381096.1 | GAA83318.1 | KF25_5312 | hydroxymethylbilane synthase, putative |
| An18g03180 | XP_391038.1 | AFUA_5G11580 | EKV15458.1 | XP_002379300.1 | GAA83336.1 | KF25_5347 | transcription factor TFIIH subunit Tfb4, putative |
| An01g02210 | XP_384450.1 | AFUA_5G11130 | EKV15495.1 | XP_002379244.1 | GAA91752.1 | KF25_5381 | PAXNEB protein superfamily |
| An13g00430 | XP_387298.1 | AFUA_7G03980 | EKV18181.1 | XP_002379155.1 | GAA89620.1 | KF25_5475 | PCI domain protein |
| An13g00870 | XP_385911.1 | AFUA_7G04320 | EKV18225.1 | XP_002372277.1 | GAA89657.1 | KF25_5519 | UBX domain protein (Ubx5), putative |
| An14g06670 | XP_382665.1 | AFUA_5G12530 | EKV18222.1 | XP_002381179.1 | GAA91404.1 | KF25_5524 | arrestin (or S-antigen), N-terminal domain protein |
| An14g05310 | XP_391008.1 | AFUA_5G13890 | EKV18757.1 | XP_002381378.1 | GAA90705.1 | KF25_5579 | SNF7 family protein |
| An02g04200 | XP_387432.1 | AFUA_2G12130 | EKV05419.1 | XP_002379510.1 | GAA82274.1 | KF25_9214 | conserved hypothetical protein |
| An02g04180 | XP_386935.1 | AFUA_2G12110 | EKV05421.1 | XP_002379508.1 | GAA82276.1 | KF25_9216 | YagE family protein |
| An02g03860 | XP_381729.1 | AFUA_2G11810 | EKV12332.1 | XP_002379484.1 | GAA82300.1 | KF25_7545 | pre-rRNA processing protein Rrp12, putative |
| An15g01510 | XP_385325.1 | AFUA_6G03950 | EKV12204.1 | XP_002378352.1 | GAA90629.1 | KF25_7617 | phospholipid-translocating P-type ATPase, putative |
| An15g01330 | XP_387080.1 | AFUA_6G04090 | EKV12222.1 | XP_002378333.1 | GAA90647.1 | KF25_7647 | DUF28 domain protein |
| An16g07940 | XP_382708.1 | AFUA_8G04880 | EKV19903.1 | XP_002372821.1 | GAA89295.1 | KF25_8606 | COP9 signalosome subunit 1 (CsnA), putative |
| An11g10890 | XP_386235.1 | AFUA_3G05740 | EKV16731.1 | XP_002384235.1 | GAA85668.1 | KF25_0830 | aldose 1-epimerase, putative |
| An10g00360 | XP_390263.1 | AFUA_2G01210 | EKV04255.1 | XP_002378601.1 | GAA91855.1 | KF25_0628 | ATP dependent RNA helicase (Dbp5), putative |
| An07g06770 | XP_390021.1 | AFUA_2G02130 | EKV19313.1 | XP_002377775.1 | GAA85341.1 | KF25_0521 | fatty acid desaturase, putative |
| An11g09700 | XP_389624.1 | AFUA_3G06600 | EKV19155.1 | XP_002383950.1 | GAA85770.1 | KF25_0418 | siroheme synthase, putative |
| An02g13850 | XP_389071.1 | AFUA_3G06960 | EKV18980.1 | XP_002383987.1 | GAA84260.1 | KF25_0382 | 60S ribosomal protein L21, putative |
| An04g00140 | XP_384747.1 | AFUA_4G05940 | EKV05287.1 | XP_002377274.1 | GAA82527.1 | KF25_0170 | choline phosphate cytidylyltransferase Muq1, putative |
| An07g08810 | XP_389550.1 | AFUA_2G04080 | EKV09612.1 | XP_002373684.1 | GAA85489.1 | KF25_3973 | GPR/FUN34 family protein |
| An07g07610 | XP_380992.1 | AFUA_2G03170 | EKV09298.1 | XP_002381074.1 | GAA85407.1 | KF25_4126 | cyclic nucleotide-binding domain protein |
| An07g07520 | XP_384328.1 | AFUA_2G03110 | EKV09304.1 | XP_002381084.1 | GAA85400.1 | KF25_4133 | alkaline phosphatase Pho8 |
| An04g04040 | XP_381261.1 | AFUA_4G09090 | EKV09362.1 | XP_002376936.1 | GAA82851.1 | KF25_4185 | thioredoxin, putative |
| An18g05750 | XP_386525.1 | AFUA_1G06690 | EKV09192.1 | XP_002381962.1 | GAA90034.1 | KF25_4201 | RLI and DUF367 domain protein |
| An18g04110 | XP_380924.1 | AFUA_1G03590 | EKV08986.1 | XP_002383185.1 | GAA83414.1 | KF25_2731 | importin 11, putative |
| An02g07570 | XP_382947.1 | AFUA_3G11750 | EKV08637.1 | XP_002374593.1 | GAA88896.1 | KF25_2836 | oxysterol binding protein (Osh5), putative |
| An02g06370 | XP_380999.1 | AFUA_3G10710 | EKV08694.1 | XP_002374708.1 | GAA82122.1 | KF25_2895 | conserved hypothetical protein |
| An01g13070 | XP_386820.1 | AFUA_1G14940 | EKV10467.1 | XP_002373387.1 | GAA86733.1 | KF25_4540 | protein translocation complex componenet (Npl1), putative |
| An01g03570 | XP_381102.1 | AFUA_1G04540 | EKV11299.1 | XP_002374454.1 | GAA88697.1 | KF25_1943 | NADH-cytochrome b5 reductase, putative |
| An03g06860 | XP_381208.1 | AFUA_8G02760 | EKV11571.1 | XP_002384968.1 | GAA90833.1 | KF25_6275 | mitochondrial ornithine carrier protein AmcA/Ort1, putative |
| An02g03290 | XP_389764.1 | AFUA_2G11320 | EKV19116.1 | XP_002379429.1 | GAA82344.1 | KF25_4260 | conserved hypothetical protein |
| An02g03570 | XP_385846.1 | AFUA_2G11540 | EKV11962.1 | XP_002379456.1 | GAA82326.1 | KF25_9534 | ketoreductase, putative |
| An13g03680 | XP_384132.1 | AFUA_3G01840 | EKV10562.1 | XP_002385378.1 | GAA92577.1 | KF25_3643 | MFS transporter, putative |
| An02g09020 | XP_381003.1 | AFUA_3G13150 | EKV16237.1 | XP_002376088.1 | GAA84623.1 | KF25_8389 | deacetylase complex subunit Sds3, putative |
| An08g04880 | XP_380839.1 | AFUA_1G12250 | EKV06071.1 | XP_002380269.1 | GAA88468.1 | KF25_9517 | mitochondrial hypoxia responsive domain protein |
| An08g06440 | XP_390392.1 | AFUA_1G13370 | EKV06162.1 | XP_002375825.1 | GAA88575.1 | KF25_6071 | aflatoxin B1-aldehyde reductase GliO-like, putative |
| An08g05530 | XP_382099.1 | AFUA_1G12760 | EKV06203.1 | XP_002375913.1 | GAA88511.1 | KF25_6130 | adenylyl cyclase-associated protein (cap) |
| An02g08420 | XP_390899.1 | AFUA_3G12480 | EKV05898.1 | XP_002372888.1 | GAA88974.1 | KF25_7409 | conidiophore development protein HymA |
| An04g03130 | XP_380452.1 | AFUA_4G08350 | EKV17184.1 | XP_002377000.1 | GAA82780.1 | KF25_8657 | monosaccharide-P-dolichol utilization protein, putative |
| An05g00810 | XP_380686.1 | AFUA_2G08190 | EKV13475.1 | XP_002378144.1 | GAA86387.1 | KF25_0962 | tubulin-specific chaperone Rbl2, putative |
| An05g00850 | XP_380271.1 | AFUA_2G08230 | EKV13472.1 | XP_002378141.1 | GAA86390.1 | KF25_0965 | MFS transporter, putative |
| An05g00880 | XP_390566.1 | AFUA_2G08300 | EKV13467.1 | XP_002378134.1 | GAA86391.1 | KF25_0972 | DnaJ domain protein, putative |
| An12g03960 | XP_386818.1 | AFUA_2G08600 | EKV13613.1 | XP_002378104.1 | GAA86428.1 | KF25_1013 | 1-acylglycerol-3-phosphate acyltransferase (AtaAp), putative |
| An12g04660 | XP_387440.1 | AFUA_2G08970 | EKV13574.1 | XP_002378064.1 | GAA86479.1 | KF25_1049 | thiamine biosynthetic bifunctional enzyme, putative |
| An01g04710 | XP_389338.1 | AFUA_1G03420 | EKV13548.1 | XP_002372928.1 | GAA88622.1 | KF25_1084 | adenylate kinase Adk2, putative |
| An18g05140 | XP_384203.1 | AFUA_1G06230 | EKV09839.1 | XP_002381926.1 | GAA89991.1 | KF25_3845 | ribosome biogenesis protein Ssf2, putative |
| An16g03410 | XP_390969.1 | AFUA_3G09780 | EKV08834.1 | XP_002373418.1 | GAA84942.1 | KF25_3012 | conserved hypothetical protein |
| An16g03690 | XP_387855.1 | AFUA_3G09970 | EKV08857.1 | XP_002383145.1 | GAA84962.1 | KF25_3034 | vacuolar iron transporter Ccc1, putative |
| An08g04390 | XP_388527.1 | AFUA_1G12070 | EKV08887.1 | XP_002380304.1 | GAA88424.1 | KF25_3069 | glycine cleavage system H protein |
| An03g03570 | XP_388175.1 | AFUA_2G14370 | EKV08307.1 | XP_002376385.1 | GAA92773.1 | KF25_3242 | conserved hypothetical protein |
| An11g05700 | XP_387040.1 | AFUA_6G08630 | EKV07953.1 | XP_002382018.1 | GAA83732.1 | KF25_3392 | mitochondrial protein, putative |
| An02g01600 | XP_384580.1 | AFUA_2G12890 | EKV12488.1 | XP_002379594.1 | GAA82474.1 | KF25_7082 | small nucleolar ribonucleoprotein complex subunit, putative |
| An02g01970 | XP_391055.1 | AFUA_2G13130 | EKV12452.1 | XP_002379668.1 | GAA82447.1 | KF25_7056 | 3' exoribonuclease family protein (Rrp42), putative |
| An07g02690 | XP_385155.1 | AFUA_7G05460 | EKV14014.1 | XP_002372561.1 | GAA86126.1 | KF25_6965 | conserved hypothetical protein |
| An09g06650 | XP_380820.1 | AFUA_5G04210 | EKV12434.1 | XP_002382898.1 | GAA88167.1 | KF25_6913 | ubiquinol-cytochrome C reductase complex core protein 2, putative |
| An15g00080 | XP_382679.1 | AFUA_6G05200 | EKV12091.1 | XP_002378220.1 | GAA92256.1 | KF25_9648 | 60S ribosomal protein L28 |
